# Multiparameter Optimization of Trypanocidal Cruzain Inhibitors With *In Vivo* Activity and Favorable Pharmacokinetics

**DOI:** 10.3389/fphar.2021.774069

**Published:** 2022-01-05

**Authors:** Ivani Pauli, Celso de O. Rezende Jr., Brian W. Slafer, Marco A. Dessoy, Mariana L. de Souza, Leonardo L. G. Ferreira, Abraham L. M. Adjanohun, Rafaela S. Ferreira, Luma G. Magalhães, Renata Krogh, Simone Michelan-Duarte, Ricardo Vaz Del Pintor, Fernando B. R. da Silva, Fabio C. Cruz, Luiz C. Dias, Adriano D. Andricopulo

**Affiliations:** ^1^ Laboratório de Química Medicinal e Computacional, Instituto de Física de São Carlos, Universidade de São Paulo, São Carlos, Brazil; ^2^ Instituto de Química, Universidade Estadual de Campinas, Campinas, Brazil; ^3^ Departamento de Bioquímica e Imunologia, Universidade Federal de Minas Gerais, Belo Horizonte, Brazil; ^4^ Departamento de Farmacologia, Universidade Federal de São Paulo, São Paulo, Brazil

**Keywords:** chagas disease, cruzain, medicinal chemistry, drug design, multiparameter optimization, pharmacokinetics, molecular modeling

## Abstract

Cruzain, the main cysteine protease of *Trypanosoma cruzi*, plays key roles in all stages of the parasite’s life cycle, including nutrition acquisition, differentiation, evasion of the host immune system, and invasion of host cells. Thus, inhibition of this validated target may lead to the development of novel drugs for the treatment of Chagas disease. In this study, a multiparameter optimization (MPO) approach, molecular modeling, and structure-activity relationships (SARs) were employed for the identification of new benzimidazole derivatives as potent competitive inhibitors of cruzain with trypanocidal activity and suitable pharmacokinetics. Extensive pharmacokinetic studies enabled the identification of metabolically stable and permeable compounds with high selectivity indices. CYP3A4 was found to be involved in the main metabolic pathway, and the identification of metabolic soft spots provided insights into molecular optimization. Compound **28**, which showed a promising trade-off between pharmacodynamics and pharmacokinetics, caused no acute toxicity and reduced parasite burden both *in vitro* and *in vivo*.

## Introduction

Endemic in Latin America, Chagas disease affects 6–7 million people worldwide and has become an emerging public health problem in nonendemic countries^1^. Among nonendemic nations, the greatest burden occurs in the United States, which is estimated to have approximately 300,000 cases of the disease (Pérez-Molina and Molina, 2018). Chagas disease kills ∼12,000 people annually, and 70 million people are at risk of infection in the Americas^2^. Moreover, the disease is an important cause of infectious cardiopathy worldwide, playing a key role in the global prevalence of cardiovascular disease (Bern, 2015; Cucunubá et al., 2016). Chagas disease significantly impacts the productivity of endemic countries, which are estimated to lose more than US $7.2 billion per year because of the disease (GBD DALYs and HALE Collaborators, 2016; Arnal et al., 2019). According to the World Health Organization (WHO), the development of innovative therapeutic approaches is required for this neglected tropical disease (NTD) because of the lack of efficient control measures and the insufficient research and development (R&D) funding. The need for novel therapeutic approaches has become more evident this year, as the WHO released a new roadmap for NTDs for 2021–2030, whose target is to eliminate the epidemics of these diseases by 2030. Chemotherapy for Chagas disease consists of benznidazole (BZ) and nifurtimox, two nitro compounds that have limited efficacy and produce serious adverse reactions that lead up to 40% of patients to discontinue treatment (Rodriques Coura and De Castro, 2002). Given these shortcomings, the development of novel, effective and safe drugs for the treatment of Chagas disease is critically needed.

Cruzain (EC 3.4.22.51), the main cysteine protease of *Trypanosoma cruzi*, has been broadly explored as a molecular target in Chagas disease drug discovery (Engel et al., 1998; McKerrow, 1999; Jose Cazzulo et al., 2001; Massarico Serafim et al., 2014). This enzyme plays a key role in all stages of the parasite’s life cycle, participating in processes such as nutrition, differentiation, evasion of the host immune system, and invasion of host cells (Ferreira and Andricopulo, 2017). Genetic studies of *T. cruzi* and the efficacy of cruzain inhibitors in reducing parasite load *in vivo* have validated the enzyme as a molecular target for the discovery of novel drugs for Chagas disease (Zanatta et al., 2008; Doyle et al., 2011; Ndao et al., 2014). Following these investigations, various classes of cruzain inhibitors, such as nitroalkenes, vinyl sulfones, thiosemicarbazones, and triazoles, have been described in the literature (Rogers et al., 2012; Avelar et al., 2015; Espíndola et al., 2015; Neitz et al., 2015; Latorre et al., 2016). In this work, we describe the design, synthesis, and *in vitro* and *in vivo* evaluations of novel benzimidazole derivatives. In addition to improving pharmacodynamic properties, such as binding affinity and potency, we evaluated the pharmacokinetic (PK) profile of newly synthesized and previously described benzimidazoles (Ferreira et al., 2014) by applying a multiparameter optimization (MPO) approach. MPO has increasingly been adopted in the early phases of pharma R&D to exclude pipeline compounds that feature poor PK profiles as early as possible (Eddershaw et al., 2000; Andricopulo and Montanari, 2005; Wang et al., 2007; Wang, 2009; Wang and Skolnik, 2009). This study led to the discovery of potent cruzain inhibitors with trypanocidal activity and innovatively contributed to the identification of compounds with improved safety and PK profiles to be explored for Chagas disease drug discovery.

## Materials and Methods

### Expression and Purification

Pro-cruzain truncated at the C-terminus was expressed and purified using a previously described protocol (Ferreira et al., 2019). *Escherichia coli* (strain M15) cultures were grown overnight at 37°C and 200 rpm in Luria Bertani (LB) medium supplemented with ampicillin (100 μg/ml) and kanamycin (50 μg/ml). Next, the cultures were diluted 10-fold in fresh LB medium supplemented with 0.5 M NaCl, 0.2% glucose, 1 mM betaine, 0.5 M sorbitol, 100 μg/ml ampicillin, and 50 μg/ml kanamycin and incubated at 37°C and 200 rpm. At an optical density (OD_600_) of 0.9, the cultures were incubated at 47°C for 20 min to promote the expression of chaperones. Then, the expression of cruzain was induced by adding isopropyl β-D-thiogalactopyranoside (IPTG) to a final concentration of 0.2 mM, which was followed by overnight incubation of the cultures at 20°C and 200 rpm. Next, the cultures were centrifuged (5,000 rpm, 30 min, 4°C), and the cells were suspended in 50 ml of lysis buffer (300 mM NaCl, 50 mM Tris-HCl, and 1.6 mg/ml lysozyme, pH 8.0) per liter of culture and lysed by sonication (12 cycles of 30 s). This cell lysate was centrifuged (9,000 rpm, 30 min, 4°C), and the supernatant was collected. Cruzain was precipitated by incubation with 35% ammonium sulfate (2 h), and this suspension was centrifuged at 9,000 rpm for 30 min at 4°C. The precipitated cruzain was resuspended in lysis buffer, and the sample was dialyzed to eliminate ammonium sulfate. The soluble fraction of the dialysate was loaded on a Ni−NTA column (Qiagen, Hilden, Germany), and the contaminants were washed using washing buffer (300 mM NaCl, 50 mM Tris-HCl, and 10 mM imidazole, pH 8.0). Cruzain was eluted by applying an increasing imidazole gradient: 25, 50, 75, 100, and 250 mM. The fractions containing cruzain were pooled together and dialyzed against 1.5 L of 0.1 M acetate buffer, pH 5.5, and then concentrated to 0.5 mg/ml. Pro-cruzain was activated by incubation with activation buffer (100 mM sodium acetate, pH 5.5, 10 mM EDTA, 5 mM DTT, and 1 M NaCl) at 37°C. The activation of cruzain was monitored by following the enzymatic activity at 30-min intervals, and the process was observed to stop after approximately 1 h. After activation, the enzyme was diluted 20-fold in binding buffer (20 mM sodium phosphate and 150 mM NaCl, pH 7.2) and added to thiopropyl Sepharose 6B resin (GE Healthcare Life Sciences, Pittsburgh, PA). After overnight incubation at 4°C, the resin was loaded on a column, and cruzain was eluted with binding buffer supplemented with 20 mM DTT. Fractions containing cruzain were pooled together and stored in 0.1 M sodium acetate, pH 5.5, at −80°C.

### Enzyme Kinetics Assays

Cruzain activity was followed by monitoring the cleavage of the fluorogenic substrate Z-Phe-Arg-aminomethyl coumarin (Z-FR-AMC), as previously described (Ferreira et al., 2019), using 96-well flat-bottom black plates and wavelengths of 355 nm for excitation and 460 nm for emission. All cruzain assays were performed in 0.1 M sodium acetate buffer with 5 mM dithiothreitol (DTT) and 0.01% Triton X-100, pH 5.5. The final concentration of cruzain was 1.5 nM, and the substrate concentration was 5.0 μM (K_m_ = 1.6 μM), except in the experiments for *K*_i_ determination, in which several concentrations of substrate were used. The cleavage of the substrate was monitored for 5 min, and the activity was calculated based on the initial reaction rates compared with the rate of a DMSO control at 30°C. The IC_50_ values were independently calculated by considering the rate measurements for at least six inhibitor concentrations, each evaluated in triplicate. To determine the mechanism of cruzain inhibition, eight concentrations of the substrate Z-FR-AMC and four concentrations of the inhibitor were employed, each in triplicate. Kinetic parameters were determined using the SigmaPlot (Systat Software Inc., Erkrath, Germany) enzyme kinetics module. Compounds were tested in two or three independent experiments. All enzyme assays were performed using varying Triton X-100 concentrations (0, 0.01, and 0.1%) (Ferreira et al., 2009). Compound concentrations were 100 μM in the single-dose percentage inhibition assays.

### Rhodesain Assays

Rhodesain activity was measured using a fluorescence-based assay as previously described (Fonseca et al., 2015). The cleavage rates of the fluorogenic substrate Z-Phe-Arg-aminomethyl coumarin (Z-FR-AMC) were monitored at wavelengths of 340 nm for excitation and 440 nm for emission. All assays were performed in triplicate in a 0.1 M sodium acetate buffer, pH 5.5, with 1 mM beta-mercaptoethanol and 0.01% Triton X-100. The final concentration of rhodesain was 3 nM, and the substrate concentration was 2.5 μM. The cleavage of the substrate was followed by continuous reading for 5 min, and enzyme activity in the presence of 100 μM of each potential inhibitor was calculated based on initial velocity rates compared to DMSO controls. All compounds were tested in triplicate in three independent experiments.

### Molecular Docking

The three-dimensional structures of the cruzain inhibitors were constructed using the standard geometric parameters embedded in SYBYL-X 2.1 (Certara, Princeton, NJ). Each compound was energetically minimized employing the Tripos force field (Clark et al., 1989) and Powell conjugate gradient method (Powell, 1977), with a convergence value of 0.05 kcal/mol.Å, and the Gasteiger-Hückel model was used for charge calculation (Gasteiger and Marsili, 1980). The molecules were docked using GOLD 5.3 (Cambridge Crystallographic Data Centre, Cambridge, United Kingdom) (Jones et al., 1997; Verdonk et al., 2003) against the X-ray structure of cruzain (PDB ID 3KKU, 1.28 Å) (Ferreira et al., 2010). The preparation of the cruzain structure consisted of removing all water molecules and inserting hydrogen atoms. The active site Cys25 was kept negatively charged, and His162 was kept protonated. The binding site was defined as a sphere with a 10 Å radius centered on the Cys25 sulfur atom. The default GOLD parameters were applied for the molecular docking runs, except for the search efficiency, which was changed to its maximum value of 200%. The generated poses were evaluated using the GoldScore scoring function, and the analysis of the binding conformations was visualized using PyMOL 3.1 (Schrödinger, New York, NY) (Lill and Danielson, 2011).

### Biological Assays Against *T. cruzi* Intracellular Amastigotes

Biological assays against *T. cruzi* intracellular amastigotes were performed as reported previously using the *T. cruzi* Tulahuen strain, which is genetically engineered to express the *E. coli* β-galactosidase gene *lacZ* (Buckner et al., 1996). β-Galactosidase catalyzes a colorimetric reaction with chlorophenol red β-D-galactopyranoside (CPRG, Sigma Chemical Co., St. Louis, MO) as the substrate. The assays were conducted in 96-well tissue culture plates, and the compounds to be tested were prepared in 100% DMSO. Epimastigotes were maintained in liver infusion tryptone (LIT) enriched with 10% fetal calf serum (FCS), streptomycin, and penicillin at 28°C. Epimastigotes were converted to trypomastigotes by incubation in Grace’s insect medium (Sigma–Aldrich, St. Louis, MO) enriched with 10% FCS at 28°C. Human HFF-1 fibroblasts were seeded at 2 × 10^3^/well in 80 μl of RPMI 1640 without phenol red and incubated overnight at 37°C and 5% CO_2_. Trypomastigotes were seeded at 1.0 × 10^4^/well in 20 μl of RPMI 1640, and the plates were incubated at 37°C and 5% CO_2_. The next day, the synthesized compounds were added (50 μl) in 3-fold serial dilutions at concentrations ranging from 0.4 to 300 μM, and the plates were incubated at 37°C and 5% CO_2_. Each compound concentration was assayed in triplicate. After 120 h, 50 μl of chlorophenol red β-D-galactopyranoside (CPRG, Sigma–Aldrich) and IGEPAL CA-630 (Sigma–Aldrich) at a final concentration of 0.1% were added. The absorbance was measured at a wavelength of 570 nm in an automated microplate reader. The data were transferred to SigmaPlot 10.0 (Systat Software Inc., Erkrath, Germany) to determine the IC_50_ values. Benznidazole (BZ, Sigma–Aldrich) was used as a positive control, and untreated wells (100% parasite growth) were used as negative controls in all plates. All compounds were tested in three independent assays.

### Cytotoxicity Assays in HFF-1 Fibroblasts

The synthesized compounds were evaluated for their cytotoxicity against HFF-1 cells using the MTS assay (Promega, Madison, WI) (Barltrop et al., 1991) as previously described (Ferreira et al., 2019). HFF-1 fibroblasts were plated at 2 × 10^3^/well in 96-well culture plates in RPMI 1640 without phenol red enriched with 10% FCS and incubated overnight at 37°C and 5% CO_2_. Next, 7 concentrations (0.1–100 µM) of the compounds were added in 3-fold serial dilutions, each concentration in triplicate, and the plates were incubated for 72 h at 37°C and 5% CO_2_. Next, 20 µl of MTS was added to each well, and the plates were incubated for an additional 4 h at 37°C and 5% CO_2_. The absorbance was measured at 490 nm using a spectrophotometer, and the data were transferred to SigmaPlot 10.0 (Systat Software Inc., Erkrath, Germany) to determine the IC_50_ values. Doxorubicin (Sigma–Aldrich) was used as a positive control, and untreated wells (100% growth) were used as negative controls in all plates. All compounds were tested in two independent assays.

### *In Vitro* Metabolic Stability in Liver Microsomes

Isolated mouse (BD Gentest, Bedford, MA) and human liver microsomes (XenoTech, Kansas City, KS) (Plant, 2004) were added at a final concentration of 0.25 mg/ml to a solution containing 40 mM dibasic potassium phosphate and 10 mM monobasic potassium phosphate. Stock solutions of compounds at 5 mM were prepared in 100% DMSO. A 50:50 quenching solution of acetonitrile (ACN) and methanol (MeOH) was prepared. An NADPH solution was prepared at 10 mM. The preparations containing the microsomes were added to each well of the incubation plate (450 µl), which was then heated to 37°C for 10 min. The compounds were added to the respective wells of the test plate (2 µl). Then, 300 µl of the microsome preparation was added to each well of the test plate. The test plate was heated under gentle rotation for 5 min at 37°C. Next, 90 µl of the mixture contained in the test plate was added to the incubation plate, making a final volume of 540 µl. Samples were collected at the following incubation times: 0, 5, 10, 15, 20, and 30 min. To the 0 min sample plate, quenching solution (180 µl), NADPH (6 µl), and the incubation plate mixture (54 µl) were added to each well. The sample plate was then sealed, homogenized, and stored at 4°C. Then, 54 µl of the NADPH solution was added to the incubation plate, which was homogenized. Before the collection of each sample at the established times, 45 µl of the quenching solution was added to each well of the corresponding sample plates. After reaching the incubation times, 60 µl of the mixture contained in the incubation plate was added to the corresponding sample plates. The sample plates were then sealed and stored at 4°C. After the collection of the last sample (30 min), all sample plates were centrifuged at 3,800 rpm for 30 min. The supernatant from each well was collected and transferred to clean plates for mass spectrometry. Five to 10 µl of each sample was injected into an AB Sciex Triple Quad 5500 LC-MS/MS instrument.

### *In Vitro* Metabolic Stability Using Recombinant CYP Enzymes

Stock solutions of compounds at 5 mM were prepared in 100% DMSO. An NADPH solution at 10 mM was prepared in a 50 mM potassium phosphate buffer. A solution at 100 pmol/ml of each of the recombinant CYP450 enzymes (1A2, 2C8, 2C9, 2C19, 2D6, and 3A4) was prepared in 50 mM potassium phosphate buffer (Proctor et al., 2004). A 50:50 quenching solution of ACN and MeOH was prepared. 320 µl of each CYP solution was added to the incubation plate, and 1 µl of each compound was added to the compound plate. Then, 100 µl of each CYP solution was added to the compound plate, and 10 µl from the compound plate was added to the incubation plate. The incubation plate was heated to 37°C with 600 rpm rotation for 10 min. To the 0 min plate, 30 µl of quenching solution, 1 µl of NADPH, and 9 µl of the incubation plate solution were added to each well, and the plate was sealed and stored at 4°C. Next, 10-µl samples were collected from the incubation plate at different incubation times (5, 10, 20, 30, and 60 min). For each time point, a separate plate containing 30 µl of quenching solution and incubation plate solution was used. After collecting the last sample, the plates were centrifuged for 20 min at 3,000 rpm. The supernatant from each well was collected and transferred to new plates for mass spectrometry. Five to 10 µl of each sample was injected into an AB Sciex Triple Quad 5500 LC-MS/MS instrument. The compounds were tested at a final concentration of 5 µM.

### *In Vitro* Metabolic Stability in Hepatocytes

Rat and human hepatocytes were used (McGinnity, et al., 2004). Samples were collected at six different time points during the incubation period and analyzed by LC-MS/MS to determine the T_½_ and the intrinsic clearance. Stock solutions of the CYP inhibitors azamulin (8.75 mM) and 1-ABT (350 mM) were prepared. Next, the test compounds were added to 96-well plates and incubated in Williams medium (Invitrogen, A12176-01) supplemented with 2 mM L-glutamine and 15 mM HEPES to reach a final concentration of 2 µM. Then, compounds (50 µl) were transferred to other plates, one plate for each time point (0, 15, 30, 60, 120, and 240 min), and incubated at 37°C and 5% CO_2_ for 30 min. Cryopreserved hepatocytes were heated in a wet bath at 37°C and dispensed in InVitroGRO HT medium supplemented with 10% FCS, 0.15 µM hydrocortisone, 0.2 mg/ml BSA, fructose, insulin, and amino acids. The cells were centrifuged at 500 rpm for 5 min. The supernatant was discarded, and the cells were resuspended in 1 ml of incubation medium heated to 37°C. Next, the cells were counted and diluted to 1 × 10^6^/ml in incubation medium. The cell suspension was divided into 3 samples: without CYP inhibitors; with 25 µM azamulin, a CYP3A4 inhibitor; and with 1 mM 1-ABT, an inhibitor of all CYPs. Each hepatocyte sample with 12,500 cells/well was incubated with either azamulin or 1-ABT for 30 min (except the group without CYP inhibitor). To the plates with the test compounds, 12,500 cells/well were added. The plates were incubated in a shaker at 37°C, 5% CO_2_, and 300 rpm. At the specified time points, the hepatocyte enzymatic activity was interrupted by the addition of 75 µl of cold ACN, and the samples were read by LC-MS/MS.

### Parallel Artificial Membrane Permeability Assay

The permeability of the compounds was assessed using the PAMPA method (Yu et al., 2015). The test compounds were dissolved in DMSO to a concentration of 5 mM. Next, the compounds were diluted in a stock plate in saline phosphate buffer (PBS), pH 6.5, containing 1% DMSO to a concentration of 1 µM. Then, 300 µl of each test compound was added to the donor plate. Afterward, 200 µl of PBS buffer, pH 7.4, was added to the acceptor plate. The donor plate was attached to the acceptor plate. The assembled acceptor-donor plate was then incubated at 37°C for 5 h under gentle agitation. To analyze the concentration of the compounds by mass spectrometry, two analysis plates (one for the donor and another for the acceptor plate) containing 300 μl of MEOH:ACN 50:50 were prepared. To the donor analysis plate, 90 µl of PBS, pH 6.5, and 10 µl of the content of the donor plate were added. For the acceptor analysis plate, 100 µl of the content of the acceptor plate was added. To monitor any potential decomposition/intrinsic instability of the test compounds in solution, samples from the stock plate were subjected to LC-MS/MS. The compounds that presented P_e_ ≥ 1.5 × 10^−6^ cm/s were classified as permeable, while compounds that presented P_e_ < 1.5 × 10^−6^ cm/s were considered poorly permeable or nonpermeable.

### Experimental Distribution Coefficient

For the eLog*D* assays (Waring, 2010), each compound (5 μl) from a 5 mM stock solution was diluted in 245 μl of a solution containing buffers A (5% MeOH, 10 mM ammonium acetate, pH 7.4) and B (100% MeOH, pH 7.4) (50:50). Nine control compounds with known eLog*D* and column retention times (acyclovir, atenolol, antipyrine, fluconazole, metoprolol, carbamazepine, ketoconazole, tolnaftate, and amiodarone; eLog*D* values ranging from −1.86 to 6.1) were subjected to LC-MS/MS in triplicate before and after the analysis of the test compounds. Retention times were recorded for each control and test compound in a C18 column. The retention time of each control compound was plotted against the respective eLog*D* values described in the literature (Lombardo et al., 2002; Lombardo et al., 2004; Alelyunas et al., 2010). The resulting linear equation (y = mx + b) was used to calculate the eLog*D* values of the test compounds, in which *x* is the retention time in minutes and *y* is the eLog*D* value.

### Fraction Unbound

The fraction unbound (*fu*) (Masimirembwa et al., 2003) of the test compounds was determined after incubation with different media, namely, plasma, microsomes, and buffer with 10% FCS. Equilibrium dialysis was performed in a 96-well plate (HT-Dialysis, Gales Ferry, CT) in which each well was divided by a semipermeable membrane (12–14 kDa cutoff). The test compounds diluted in either plasma, microsome suspension, or buffer were added to one side of the membrane. Potassium phosphate buffer (50 mM, pH 7.4) was added to the other side of the membrane. A standard curve was used to calculate the compound concentration (*fu*) on each side of the membrane. Stock solutions of each compound were prepared in 100% DMSO to obtain a final concentration of 1 mM. The following compound concentrations were used: 1, 2, 20, 200, 2,000 and 5,000 nM. To 500 μl of medium, 0.5 μl of the compound stock solution was added, and this was applied to one side of the well. Each compound was evaluated in triplicate. Next, the plate was assembled, sealed, and incubated at 37°C under rotation (150 rpm) for 4 h. After the incubation period, the plates were subjected to LC-MS/MS.

### Biotransformation and Analysis of Metabolites

Stock solutions of compounds at 5 mM were prepared in 100% DMSO for the biotransformation analyses (Obach, 1999). Test solutions of compounds at 1 mM were prepared in H_2_O/MeOH: 2/1 (v/v). A solution of 10 mM NADPH, a 50:50 quenching solution of ACN and MeOH, and 100 mM phosphate buffer was prepared. Isolated mouse (BD Gentest, Bedford, MA) and human liver microsomes (XenoTech, Kansas City, KS) were dissolved to 2 mg/ml in phosphate buffer. One plate for the 0 min time point and another for the 60 min time point were prepared. To these plates, 178 μl of the microsome solution and 2 μl of the compound stock solution (final test concentration of 5 μM) were added. The plates were incubated at 37°C for 5 min under gentle agitation. Next, 400 μl of the quenching solution was added to the 0 min plate, which was followed by the addition of 20 μl of NADPH. The plate was sealed and kept under refrigeration (4°C). To the 60 min plate, 20 μl of NADPH was added, and the plate was sealed and incubated at 37°C for 60 min under gentle agitation. Next, 400 μl of quenching solution was added to the 60 min plate. The two plates were centrifuged (4°C, 3300 rpm, 30 min), and the supernatant was collected for mass spectrometry.

### *In Vivo* Pharmacokinetics

The *in vivo* pharmacokinetic profiles of the compounds were determined using male CD1 mice weighing 50 g (Davies and Morris, 1993; Liu and Jia, 2007). The compounds were administered in a single dose orally (0.5 mg/kg) and intravenously (0.5 mg/kg). Stock solutions of the compounds in DMSO were diluted in Tween 80, PEG-400, and D5W (5% dextrose in water) at a ratio of 2:5:20:73 (v/v). The injection volume was 10 ml/kg. The remaining plasma concentration was monitored over time by LC-MS/MS by collecting blood samples (40 μl) at 10, 25, and 50 min and 1, 3, 6, 9, 12, and 24 h after administration of the compound.

### Pharmacokinetics Analysis by LC-MS/MS

For the biotransformation experiments and analysis of metabolites in microsomes, an Ultra-High-Pressure Liquid Chromatography instrument (UHPLC, Thermo Accela, Waltham, MA) (Spaggiari et al., 2014) connected to an automatic sample injector and a 1250 series pump was used. The UHPLC system was connected to a Thermo Fisher (Waltham, MA) LTQ Orbitrap mass spectrometer. For all other analyses, an AB Sciex Triple Quad 5500 coupled to a UHPLC equipped with a UV 1290 diode detector (Agilent Technologies, Santa Clara, CA) and a CTC PAL self-collecting system (LEAP Technologies, Carrboro, NC) was used. Q1 MS positive ion mode (300–500 Da) was used to detect the ions of the parent compounds. The UV detector was operated in spectral mode (250–280 nm). A Hypersil Gold C18 (2.1 mm × 100 mm, 1.9 µm, Thermo Fisher) HPLC column was used. The mobile phases were solvent A (0.1% formic acid in water) and solvent B (0.1% formic acid in ACN). The flow was adjusted to 0.55 ml/min, and the injection volume was adjusted to 20 ml. The gradient started with 1% solvent B for 0.4 min, reached 40% (solvent B) in 2.3 min and 95% (solvent B) in 0.67 min, was maintained for 0.5 min, and returned to the initial condition of 1%. This condition was maintained for 1 min before injection of the next sample. The peak area ratio (peak area of the test compounds/peak area of the control compounds) was converted to the percentage of remaining compound, with the 0 min time point ratio set to 100%. T_½_ and CL_int_ were calculated from the percentage of remaining compound versus the incubation time. From the resulting function, the slope (*k*) was determined. The equations T_1/2 (min)_ = ln(2)/*k* and CL_int_ _*in vitro*_ _(μL/min/mg)_ = k*1000/0.25 were used to determine T_½_ and CL_int_.

### Animals for the *In Vivo* Assays

Thirty-day-old female Swiss mice weighing 20–25 g and procured from the Center for the Development of Experimental Models for Medicine and Biology (CEDEME/UNIFESP) served as the subjects for these experiments. Animals were housed (5–6 per cage) in polypropylene cages and kept under controlled temperature (22–23°C) and humidity on a 12-h light/dark cycle (12 h light, 12 h dark; lights on at 6:30 am). Rodent chow and water were available ad libitum throughout the experiments. The Committee of Ethics in Research of the Universidade Federal de São Paulo approved all the experiments (CEUA n° 5301080816).

### Chemistry

Unless stated otherwise, all reactions were performed under an atmosphere of argon with dry solvents and magnetic stirring (detailed organic synthesis methods are in the Supplementary Material). Dichloromethane (DCM) and triethylamine (Et_3_N) were distilled from CaH_2_. Tetrahydrofuran (THF) was distilled from sodium/benzophenone. Dimethyl formamide (DMF) was purchased from Aldrich (anhydrous) and used without further purification. Yields refer to homogeneous materials obtained after purification of reaction products by flash column chromatography using silica gel (200–400 mesh) or recrystallization. Analytical thin-layer chromatography was performed on silica gel 60 and GF (5–40 μm thickness) plates, and the plates were treated with a basic potassium permanganate stain or ninhydrin solution, heated and visualized under UV light. Melting points were measured with a Buchi M-565 instrument and are uncorrected. ^1^H and proton-decoupled ^13^C NMR spectra were acquired in CDCl_3_, CD_3_OD or *d*_6_-DMSO at 250 MHz (^1^H) and 62.5 MHz (^13^C) (Bruker DPX250), 400 MHz (^1^H) and 100 MHz (^13^C) (Bruker AVANCE 400), 500 MHz (^1^H) and 125 MHz (^13^C) (Varian Inova 500), or 600 MHz (^1^H) and 150 MHz (^13^C) (Bruker AVANCE 600). Chemical shifts (δ) are reported in ppm using residual undeuterated solvent as an internal standard (CDCl_3_ at 7.26 ppm, CD_3_OD at 3.31 ppm, *d*_6_-DMSO at 2.50 ppm, and TMS at 0.00 ppm for ^1^H NMR spectra and CDCl_3_ at 77.16 ppm, CD_3_OD at 49.0 ppm, *d*_6_-DMSO at 39.52 ppm for ^13^C NMR spectra). Multiplicity data are reported as follows: s = singlet, d = doublet, t = triplet, q = quartet, br s = broad singlet, dd = doublet of doublets, dt = doublet of triplets, app d = apparent doublet, app t = apparent triplet, m = multiplet, and br m = broad multiplet. The multiplicity is followed by the coupling constant(s) in Hz and integration. High-resolution mass spectrometry (HRMS) was measured using electrospray ionization (ESI) (Waters xevo Q-tof, Thermo LTQ-FT ultra, or Thermo Q Exactive) or using electron ionization (EI) (GCT Premier Waters). The synthesis and characterization of compounds 1, 17, 18, 31–63 were previously reported (Ferreira et al., 2014).

## Results and Discussion

### Synthesis of Novel Benzimidazole Derivatives

**Scheme 1:**
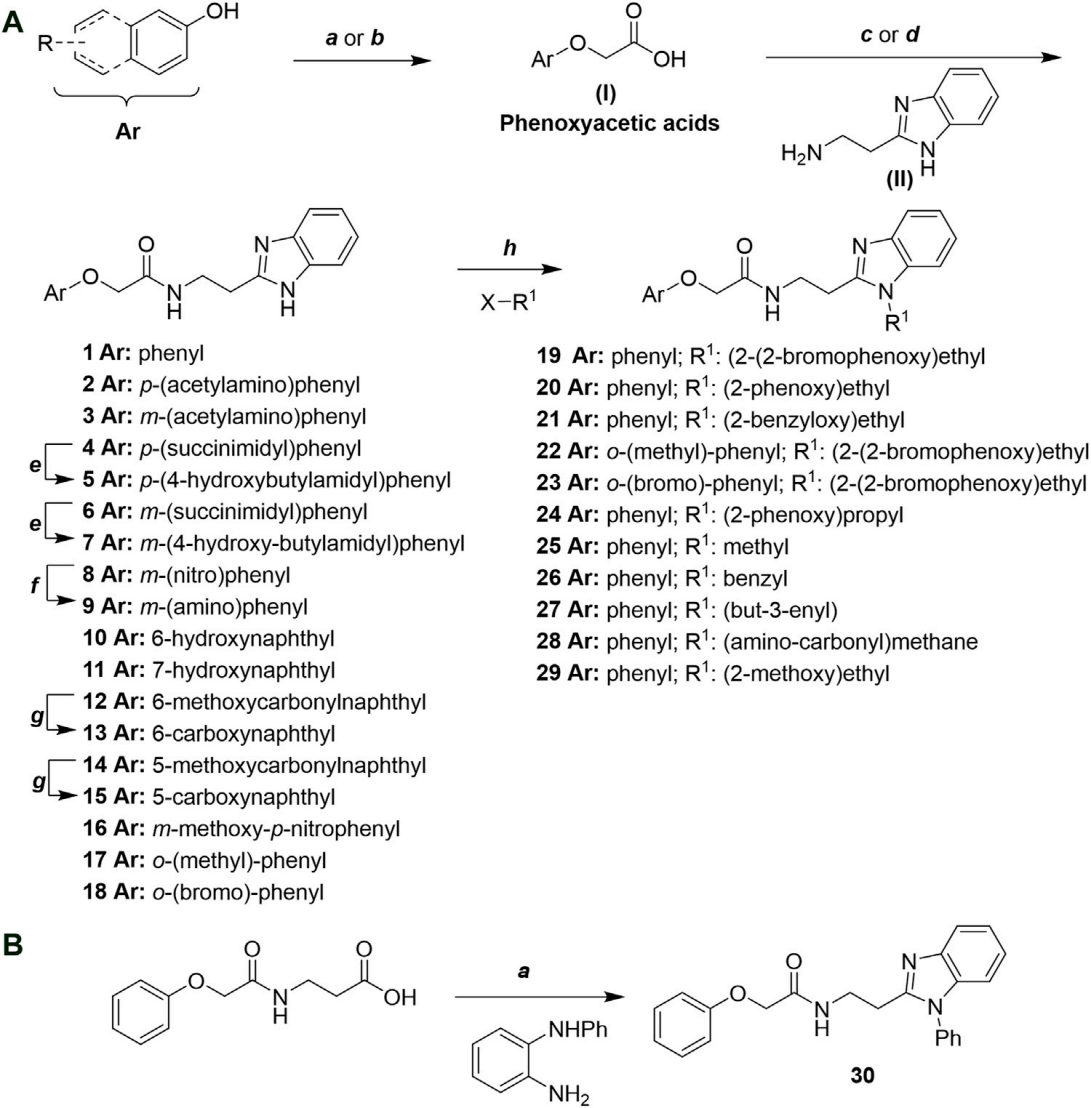
**(A)** *Reagents and conditions:* (a) i) ethyl 2-bromoacetate, K_2_CO_3_, DMF, r.t., 4–6 h; ii) NaOH (6 mol. L^−1^), MeOH, r.t., 30 min; iii) HCl (6 mol. L^−1^), 0 C, 10 min; (b) i) benzyl 2-bromoacetate, K_2_CO_3_, DMF, r.t., 4–6 h; ii) Pd/C (20%), H_2(g)_, EtOAc, MeOH, r.t., 1–2 h; (c) i) oxalyl chloride, DMF, DCM, r.t., 1 h; ii) *N*-Hydroxysuccinimide, DCM, triethylamine, 0°C, 30 min; iii) II, sodium carbonate, EtOAc, r.t., 1 h; (d) II, EDC, HOBt, trimethylamine, DMF, r.t., 8–15 h; (e) sodium borohydride, MeOH, THF, r.t., 5 h; (f) Pd/C (20%), H_2(g)_, MeOH, r.t., 2 h; (g) i) NaOH (6 mol. L^−1^), MeOH, r.t., 20 min; ii) HCl (6 mol. L^−1^), 0°C, 10 min; (h) haloalkyls, 18-crown-6, potassium *tert*-butoxide, THF, r.t. or 45°C, 13–48 h. **(B)** *Reagents and conditions:* (a) i) oxalyl chloride, DMF, DCM, r.t., 30 min, ii) *N* ^1^-phenylbenzene-1,2-diamine, *n*-butanol, 110°C, 18 h.

Phenoxyacetic acids of type I were prepared from the corresponding substituted phenols by nucleophilic substitution with 2-bromoacetic acid or nucleophilic substitution with alkyl 2-bromoacetic ester, followed by ester hydrolysis (Scheme 1A). A subsequent reaction of activated carboxylic acid I with amine II led to the formation of amides **1–4**, **6**, **8**, **10–12**, **14**, and **16–18**. Alcohols **5** and **7** and aniline **9** were prepared by reduction reactions of imides **4** and **6** with sodium borohydride and nitrobenzene derivative **8** using hydrogenation under Pd/C catalysis. Carboxylic acid derivatives **13** and **15** were synthesized by hydrolysis under basic conditions of methyl esters **12** and **14**, respectively. *N*-alkylated compounds **19–29** were synthesized by *N*-alkylation of the benzimidazole moiety of compounds **1**, **17**, and **18** with different electrophiles. *N*-Phenyl derivative **30** was prepared as described in Scheme 1B by an amidation reaction followed by cyclization and dehydration.

### Design of Novel Cruzain Inhibitors

**FIGURE 1:**
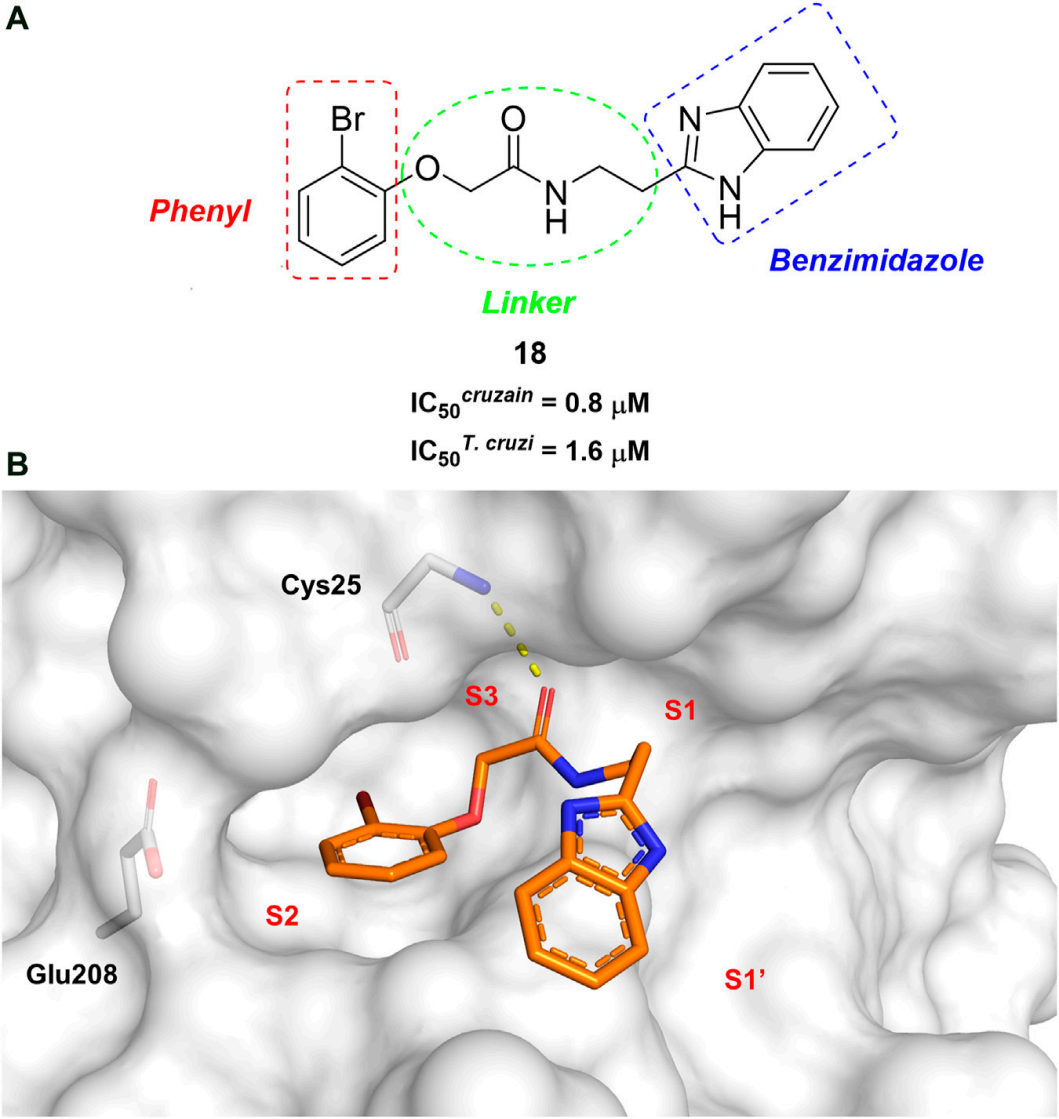
**(A)** Cruzain inhibitor **18** was used as the lead compound for the design of novel benzimidazole derivatives. **(B)** X-ray structure of compound **18** in complex with cruzain (PDB 3KKU, 1.28 Å). Binding site residues (carbon in gray) and compound **18** (carbon in orange) are shown as sticks. A hydrogen bond is shown as a dashed line. Cruzain subsites are labeled as S1, S1′, S2, and S3.

In this work, we designed a series of cruzain inhibitors based on a previously identified benzimidazole derivative (**18**, Figure 1A) (Ferreira et al., 2010; Ferreira et al., 2014). Considering the lead-like profile of compound **18** and its activity against cruzain and *T. cruzi*, we selected this compound for a lead optimization program and, for the first time, the pharmacokinetics and the *in vivo* trypanocidal ability of this molecule and its analogs were investigated. We explored compound **18** by appending diverse substituents at the phenyl and benzimidazole rings to improve both the interaction with cruzain and the PK profile. By adding substituents at the phenyl ring, we aimed to enhance the selectivity for cruzain over other proteases by promoting hydrogen bonding with Glu208, a critical residue located in the S2 subsite of the active site (Figure 1B). Glu208 is absent in most other proteases, including human cathepsins. We additionally focused on increasing the affinity and potency of the compounds by exploring *N*-substitutions at the benzimidazole and enabling additional interactions with the S1 and S1’ subsites.

### Exploring the Benzimidazole and Phenyl Rings

The structure and activity against cruzain of *N*-substituted benzimidazoles are summarized in Table 1. Three out of the derivatives that were initially evaluated showed IC_50_ values below 3 μM. Only compounds lacking the *o-*bromine at the substituent appended to the benzimidazole core were active against cruzain. No significant variation in the percent inhibition values was observed for different Triton X-100 concentrations (0, 0.01, and 0.1%), demonstrating that the inhibitors do not act as aggregators (Supplementary Table S1).

**TABLE 1:** Structure and activity against cruzain of new *N*-substituted benzimidazole derivatives.^a^

Compound	Structure	% Cruzain inhibition (100 µM)^a^	IC_50_ (µM)^b^
**1**	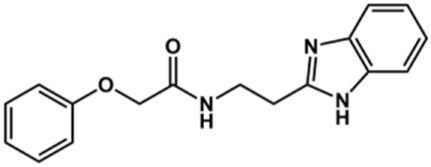	90	10.9 ± 1.0
**19**	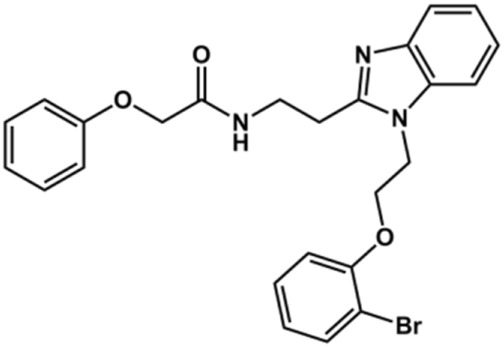	72	ND
**20**	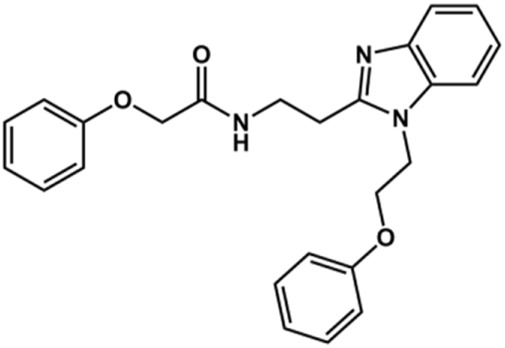	92	1.04 ± 0.7
**21**	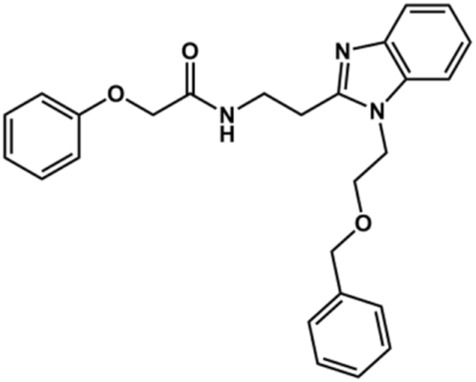	76	1.69 ± 0.4
**22**	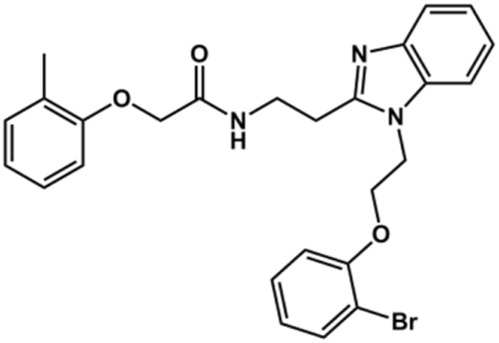	36	ND
**23**	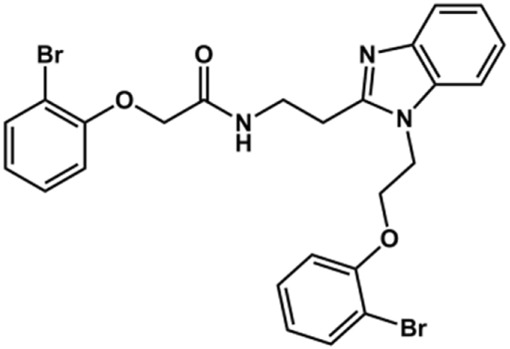	49	ND
**24**	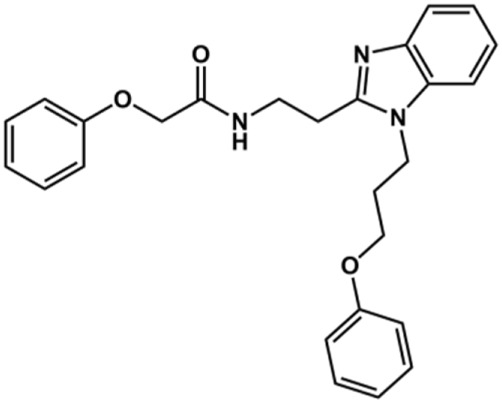	81	2.2 ± 1.2
**25**	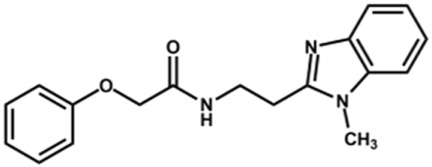	87	8.6 ± 1.7
**26**	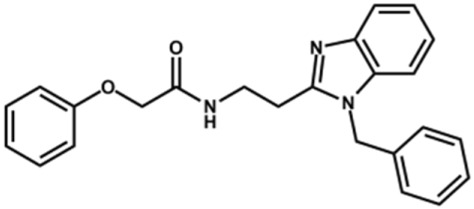	77	1.1 ± 0.2
**27**	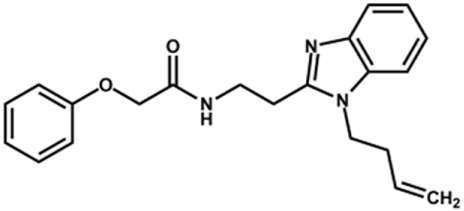	79	13.7 ± 1.4
**28**	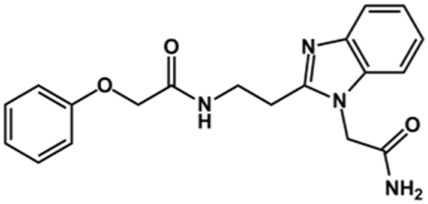	81	12.1 ± 2.4
**29**	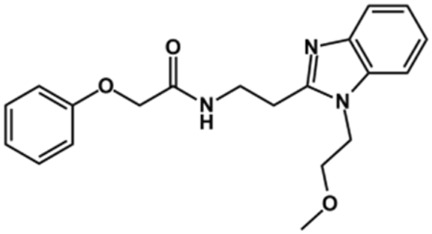	79	8.8 ± 1.8
**30**	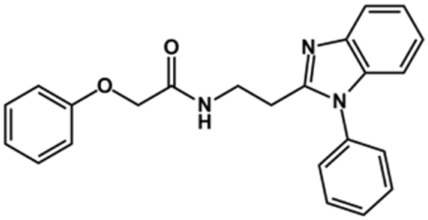	79	8.6 ± 2.6

a The percentage of inhibition refers to the mean of three experimental measures.

b IC_50_ values were determined independently in triplicate using at least six distinct inhibitor concentrations, and the values represent the mean ± SD of 2–3 independent assays.

**FIGURE 2:**
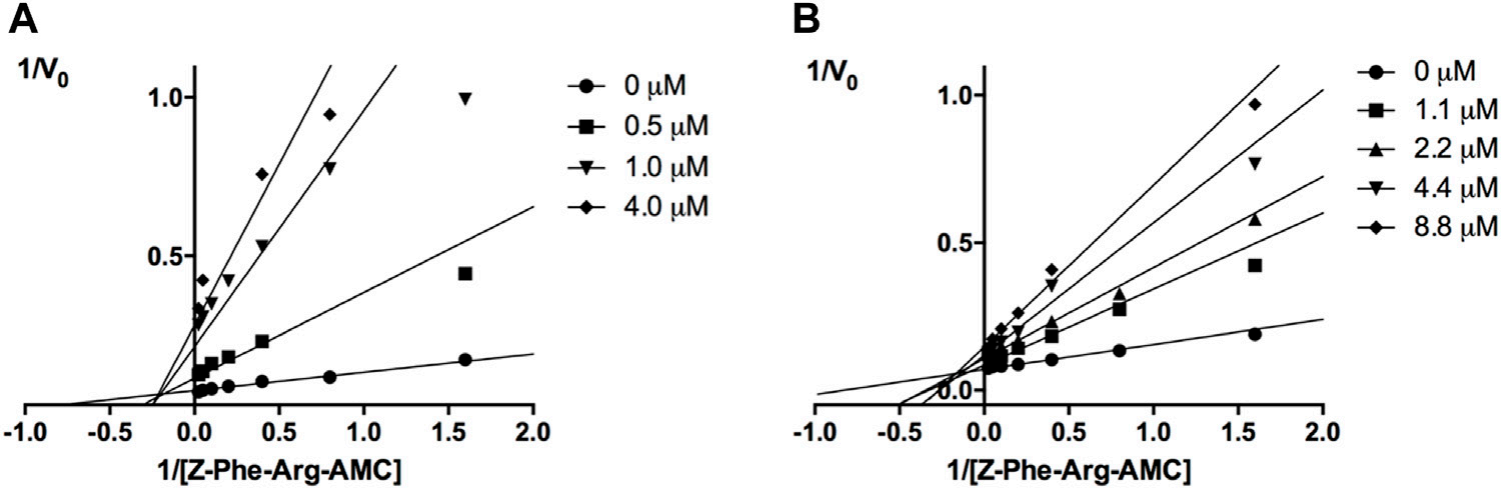
Lineweaver-Burk plots for compounds **20 (A)** and **24 (B)**. Each curve represents a different inhibitor concentration.

The mechanisms of action of compounds **20** and **24** were determined by measuring their remaining enzymatic activity in the presence of distinct concentrations of the substrate and inhibitors. Double reciprocal Lineweaver-Burk plots (Figure 2) showed that unlike the benzimidazole analogs previously described (Ferreira et al., 2014), compounds **20** and **24** act as noncompetitive cruzain inhibitors with a higher affinity for the free enzyme than for the corresponding enzyme-substrate complex. The typical behavior of noncompetitive inhibitors was additionally confirmed in another experiment, in which no significant variation in IC_50_ values was observed with increasing substrate concentrations at a constant protein concentration (Supplementary Table S2).

**FIGURE 3:**
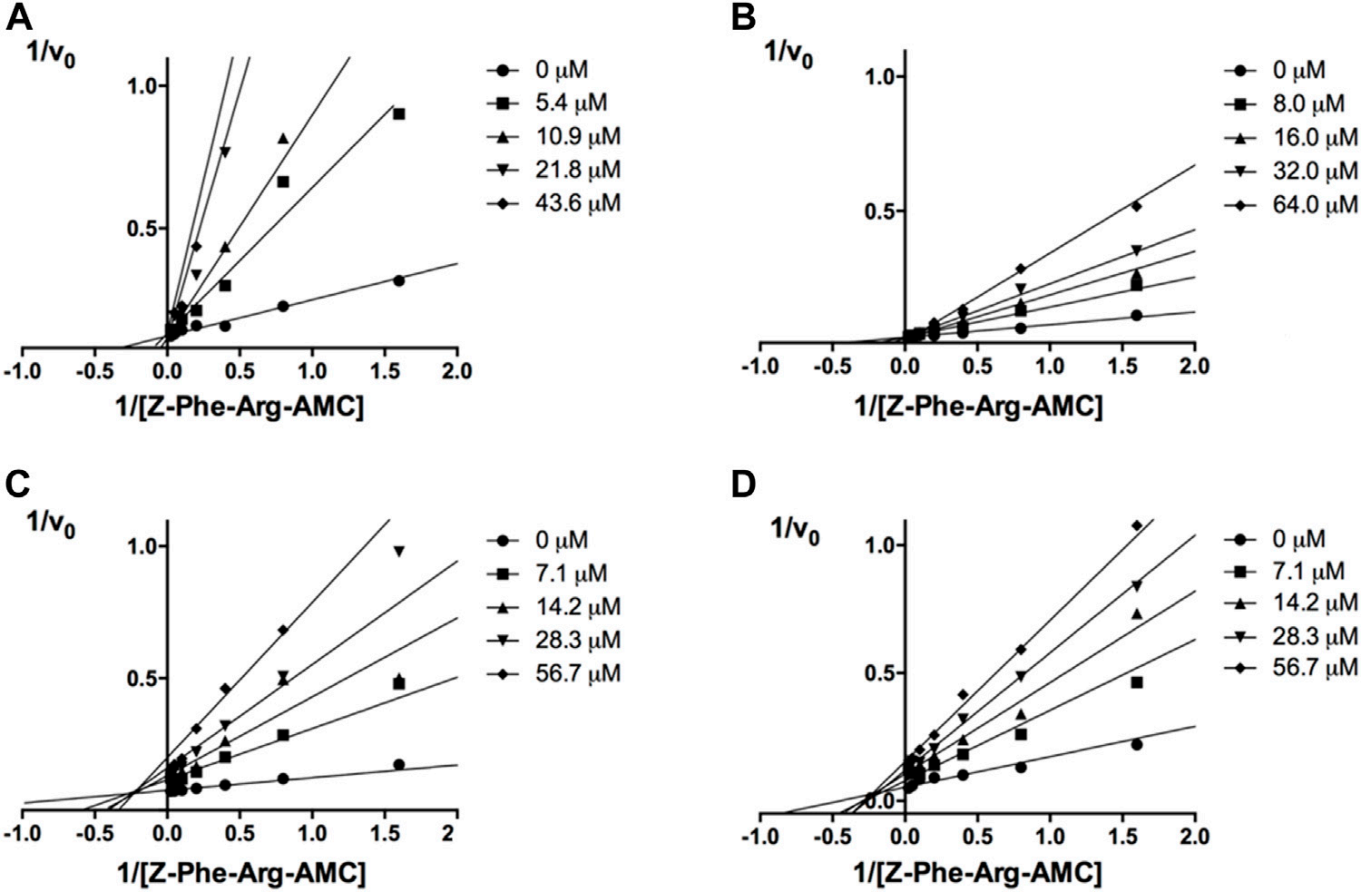
Lineweaver-Burk plots for compounds **1 (A)**; **25 (B)**; **27 (C)**; and **26 (D)**. Each curve represents a different inhibitor concentration.

Next, novel compounds were synthesized to evaluate the effect of growing the *N*-substituent on the mechanism of action against cruzain. Compound **1** (IC_50_ = 10.9 μM, Table 1), which, in contrast with lead compound **18**, lacks the *o*-bromine at the phenyl ring, is more than 10-fold less potent than **18** (IC_50_ = 0.8 μM). Installing a methyl group as the *N*-substituent also resulted in a decrease in activity (**25**, IC_50_ = 8.6 μM). As shown in Figure 3, compounds **1** and **25** act as competitive inhibitors. Growing the *N*-substituent to a benzyl (**26**, IC_50_ = 1.1 μM) enhanced the activity; however, expanding to but-3-enyl (**27**, IC_50_ = 13.7 μM) significantly reduced the activity. Interestingly, in contrast with compounds **1** and **25**, compounds **26** and **27** act as noncompetitive inhibitors (Figure 3). No significant variation in the IC_50_ values of **26** and **27** was observed with increasing substrate and constant protein concentrations, further corroborating the noncompetitive inhibition mechanism (Supplementary Table S2).

These results clearly highlight the role played by the *N*-substituents in the mechanism of cruzain inhibition. The importance of the amine was previously demonstrated by replacing the nitrogen with an oxygen atom, and the activity was lost (Ferreira et al., 2014). Additionally, the distinct *N*-substituents allowed us to correlate the substituent volume with the mechanism of inhibition. The lack of a substituent (**1**) or the presence of a methyl (**25**) results in competitive inhibition, while bulkier groups such as benzyl (**26**) and but-3-enyl (**27**) lead to noncompetitive inhibition.

**FIGURE 4:**
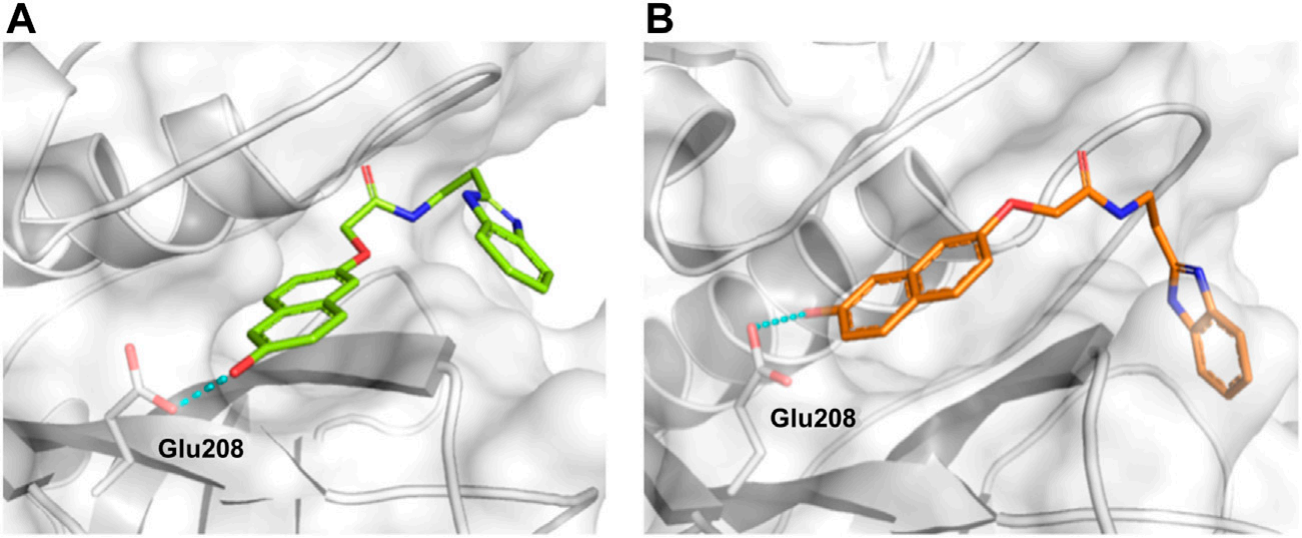
Molecular docking predicted the binding conformations of compounds **10 (A)** and **11 (B)** in complex with cruzain (PDB 3KKU, 1.28 Å), showing the formation of hydrogen bonds (dashed lines) between the hydroxyl groups and Glu208. Binding site residues (carbon in gray) and compounds **10** and **11** (carbon in green and orange, respectively) are shown as sticks.

In the next step, we explored substitutions at the phenyl ring. Given that the phenyl ring of lead compound **18** occupies the S2 pocket of the cruzain-binding site, we expanded the phenyl into a naphthyl system and appended different hydrogen bond donors and acceptors to the phenyl ring. The goal was to explore a potential interaction with Glu208. As shown in Table 2, among the 15 synthesized compounds, the three naphthyl analogs with either hydroxyl or ether at the *meta* or *para* positions were the most potent: 6-hydroxynaphthyl **10** (IC_50_ = 3.4 μM), 7-hydroxynaphthyl **11** (IC_50_ = 2.7 μM), and 6-methoxycarbonyl **12** (IC_50_ = 2.3 μM). The design concept was corroborated by molecular docking runs, which predicted the formation of a hydrogen bond between the hydroxyl groups of **10** and **11** and Glu208 (Figure 4). To further corroborate the formation of a hydrogen bond with Glu208, we evaluated the activities of six compounds against the enzyme rhodesain, a cysteine protease that has a similar active site to that of cruzain, in which Glu208 is replaced with an alanine residue (Lima et al., 2013). The activities of the compounds against cruzain were significantly more pronounced than their activities against rhodesain, indicating the importance of interactions with Glu208 for inhibition by **10** and **11** (Supplementary Table S3).

**TABLE 2:** Structure and activity against cruzain of new benzimidazoles with substituents at the phenyl ring.^a^

Compound	Structure	% Cruzain inhibition (100 μM)^a^	IC_50_ (μM)^b^
**2**	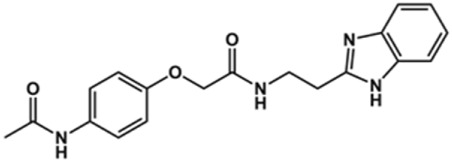	79	4.5 ± 0.5
**3**	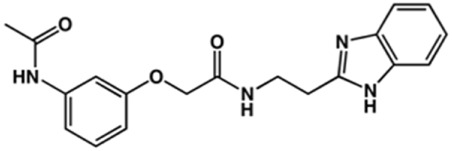	70	28.1 ± 3.1
**4**	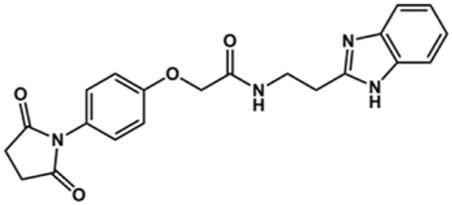	20	ND
**5**	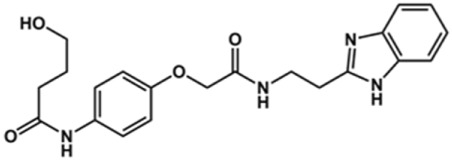	87	ND
**6**	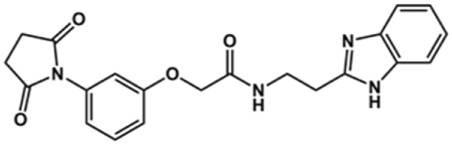	38	ND
**7**	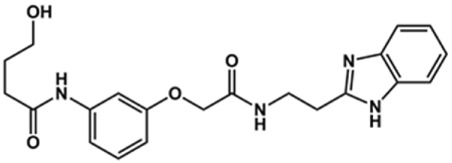	75	ND
**8**	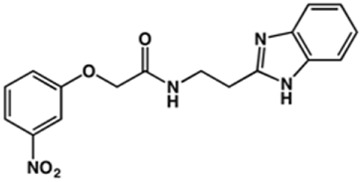	83	13.5 ± 2.6
**9**	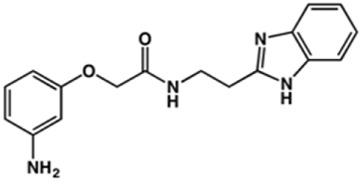	90	18.2 ± 1.8
**10**	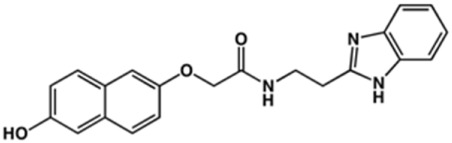	90	3.4 ± 0.9
**11**	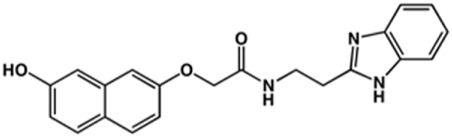	96	2.7 ± 0.7
**12**	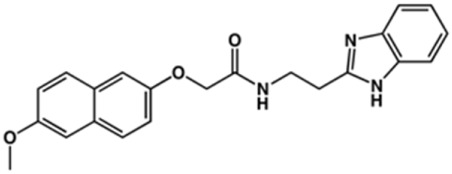	100	2.3 ± 0.6
**13**	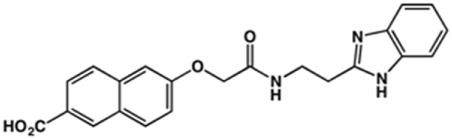	89	24.2 ± 4.5
**14**	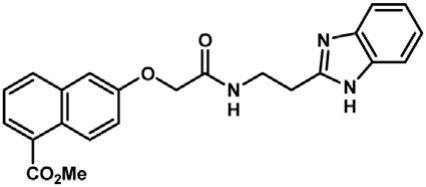	92	8.3 ± 2.1
**15**	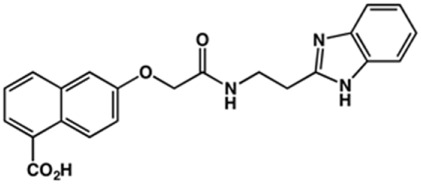	58	> 100
**16**	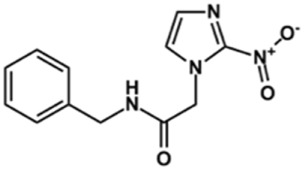	62	ND

a The percentage of inhibition refers to the mean of three experimental measures.

b IC_50_ values were determined independently in triplicate using at least six distinct inhibitor concentrations, and the values represent the mean ± SD of 2–3 independent assays.

### Trypanocidal Activity, Physicochemical Profile, and Cytotoxicity

After the enzyme inhibition studies, active compounds were evaluated for their activity against *T. cruzi* intracellular amastigotes and PK properties (Table 3). Among the *N*-substituted analogs, compounds **20** (IC_50_ = 2.04 μM) and **24** (IC_50_ = 1.43 μM) were equipotent to the reference drug BZ (IC_50_ = 1.45 μM). The only inactive compound in this series was the *N-*methyl analog **25**. In general, these compounds are more lipophilic than BZ, as shown by the Log*P* and eLog*D* values. Among the molecules in Table 3, six were classified as high-permeability compounds (PAMPA higher than 1.5 × 10^−6^ cm/s), and nine were classified as low-permeability compounds (PAMPA lower than 1.5 × 10^−6^ cm/s).

**TABLE 3:** *In vitro* activity against *T. cruzi* and physicochemical properties of a subset of the benzimidazoles.

Compound	Structure	IC_50_^*T. cruzi*^ (μM)^a^	PAMPA (×10^−6^ cm/s)	eLog*D*	Log*P*	PSA (Å^2^)
BZ^a^	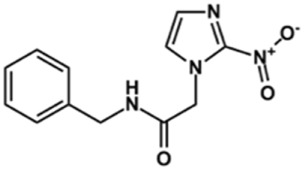	1.45 ± 0.4	3.17	0.84	1.00	92.70
N-substituted Analogs						
**1**	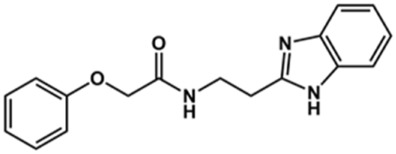	3.9 ± 0.3	4.21	2.90	2.41	67.00
**20**	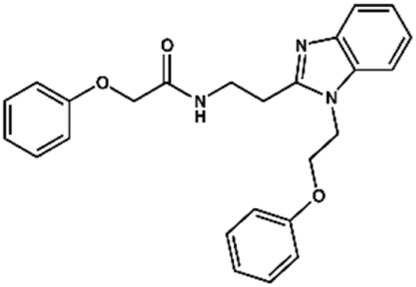	2.04 ± 0.6	4.30	4.24	1.57	104.38
**24**	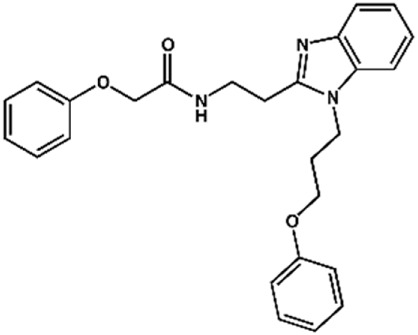	1.43 ± 0.4	2.29	4.45	4.31	65.38
**25**	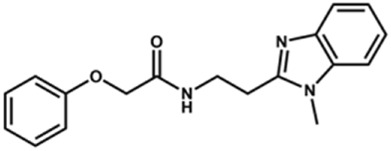	≅ 100	1.46	2.93	2.62	56.15
**26**	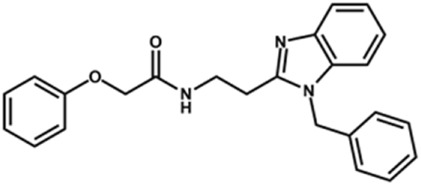	7.4 ± 2	8.72	4.06	4.20	56.15
**27**	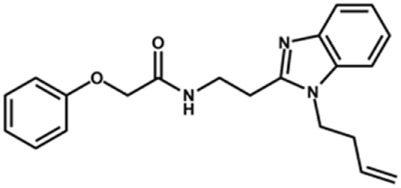	6.9 ± 2.2	9.06	3.75	3.56	56.15
**28**	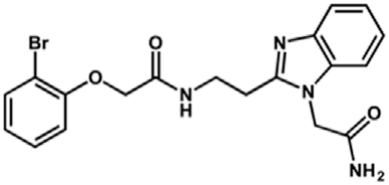	6.8 ± 0.9	0.71	2.16	1.47	99.24
Phenyl-substituted Analogs						
**2**	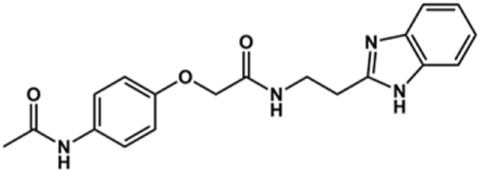	4.5 ± 0.6	0.21	1.92	1.53	96.10
**3**	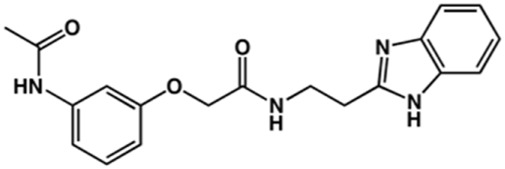	5.0 ± 1.0	0.17	2.26	1.53	96.10
**8**	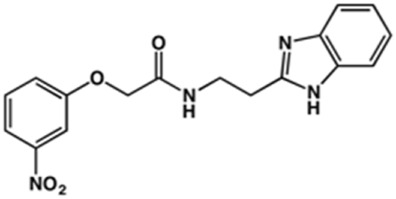	3.5 ± 0.7	2.17	2.98	2.31	112.83
**9**	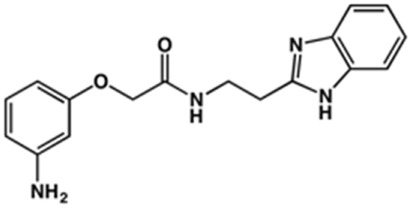	5.4 ± 0.9	0.38	1.88	1.67	93.03
**10**	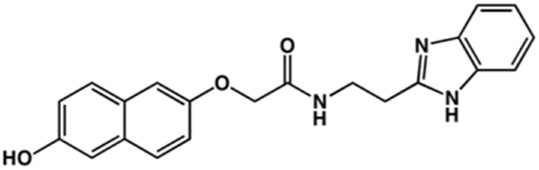	14.6 ± 0.7	0.21	3.17	3.08	87.24
**11**	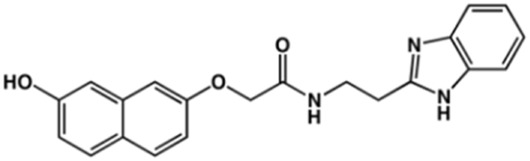	2.8 ± 0.6	0.17	2.26	3.08	87.24
**12**	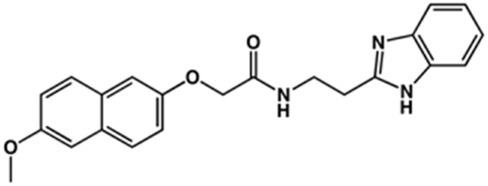	16.6 ± 2.4	0.34	3.89	3.18	93.31
**13**	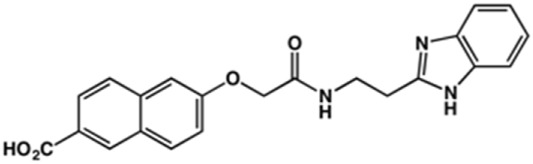	>50	0.83	ND	3.31	76.24

a IC_50_ values represent the mean ± SD of three independent assays; BZ, benznidazole. eLog*D* and PAMPA were experimentally determined. Log*P* and PSA were predicted computationally.

Most compounds with substituents on the phenyl ring were active against *T. cruzi*, with IC_50_ values in the low micromolar range (Table 3). The exception was compound **13**, which has a 6-carboxynaphthyl moiety. Compound **13** showed moderate activity against cruzain (IC_50_ = 24.2 μM) in addition to a Log*P* value higher than those of the other analogs. The combination of these two properties may be the cause of the lack of trypanocidal activity of this compound.

The benzimidazole derivatives were further evaluated regarding their cytotoxicity against human HFF-1 fibroblasts, which were used as host cells for *T. cruzi* (Table 4). Selectivity indices (SI), which express the ratio between the IC_50_ values for HFF-1 cells and *T. cruzi,* were calculated. Overall, the evaluated compounds exhibited no significant toxicity against human HFF-1 fibroblasts. Three compounds showed SI values comparable to or greater than that of the reference drug BZ (SI > 33): **18** (SI > 61), **17** (SI > 35), and **37** (SI > 34). It is worth noting that compounds **1** (SI > 26) and **8** (SI > 29) also exhibited suitable SI values.

**TABLE 4:** Biological evaluation of a subset of the benzimidazoles against *T. cruzi* and human HFF-1 fibroblasts.

Compound	IC_50_^*T. cruzi*^ (µM)^a^	IC_50_^HFF-1^ (µM)^b^	SI^c^
BZ	3.00 ± 0.60	>100	>33
Doxorubicin	—	0.26	—
**1**	3.9 ± 0.3	>100	>26
**2**	12.1 ± 1.3	>100	>8
**3**	5.0 ± 1.0	>100	>20
**5**	∼ 50.00	>100	>2
**8**	3.5 ± 0.7	>100	>29
**10**	14.6 ± 0.7	>100	>7
**17**	2.81 ± 0.75	>100	>35
**18**	1.63 ± 0.57	>100	>61
**20**	2.04 ± 0.60	>30	>14
**24**	1.43 ± 0.40	>30	>21
**26**	7.40 ± 2.00	>100	>13
**27**	6.90 ± 2.20	>100	>14
**28**	6.8 ± 0.9	>100	>15
**32**	7.90 ± 2.13	>100	>12
**33**	6.68 ± 2.35	>100	>15
**34**	16.22 ± 3.51	>30	>2
**35**	46.12 ± 6.21	>100	> 2
**37**	2.90 ± 0.66	> 100	>34
**38**	11.14 ± 3.19	>100	>9

a IC_50_ values represent the mean ± SD of three independent assays.

b IC_50_ values represent the mean ± SD of two independent assays.

c Selectivity index (SI) = IC_50_^HFF-1^/IC_50_^*T. cruzi*^.

### Determination of *In Vitro* and *In Vivo* Metabolic Stability

A series of 10 benzimidazole derivatives were selected based on their activity against cruzain and *T. cruzi* to undergo PK studies, including *in vitro* and *in vivo* metabolism. Table 5 shows the *in vitro* results for CL_int_ after incubation with human and mouse microsomes, *fu*, Log*D*, and PAMPA. Corrected clearance values (CL_int_u_) were obtained by calculating the ratio between CL_int_ and *fu*. It is important to note that only unbound drug molecules are available for clearance, interaction with metabolizing enzymes and transporters, equilibration into tissues, and pharmacological activity. Thus, PK, pharmacodynamics, and toxicity are driven by unbound drug concentrations (Zamek-Gliszczynski, et al., 2011). As such, protein binding (PPB) in plasma, microsomes, and target tissues is routinely evaluated in drug discovery to determine the respective *fu* values (Wang, et al., 2014). The drug-like space for unbound clearance lies at approximately 10 L/h/kg. As shown in Table 5, all benzimidazole derivatives have CL_int_u_ values much higher than the drug-like reference and that of the reference drug BZ. Compounds **17**, **18**, **37**, which have IC_50_^*T. cruzi*^ values comparable to that of BZ, have CL_int_u_ values ranging from ∼10 to 21 times higher than that of BZ, which could undermine the achievement of the bioavailability levels required for biological response. Most compounds listed in Table 5 had PAMPA values higher than 1.5 × 10^−6^ cm/s and were classified as having good permeability.

**TABLE 5:** *In vitro* PK profile of benzimidazole analogs.

Compound	Structure	CL_int_ (L/h/kg) human	CL_int_ (L/h/kg) mouse	*fu*	CL_int_u_ (L/h/kg) human	CL_int_u_ (L/h/kg) mouse	eLog*D*	PAMPA (×10^−6^ cm/s)
BZ	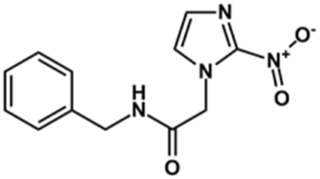	1.5	4.4	1.0	1.5	4.4	0.8	3.2
**17**	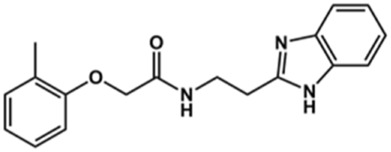	23.9	300.0	0.8	31.2	392.2	3.6	5.9
**18**	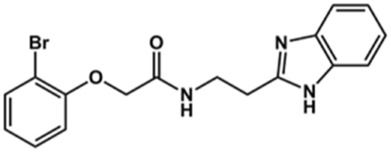	9.2	191.0	0.6	16.0	334.5	3.9	5.6
**31**	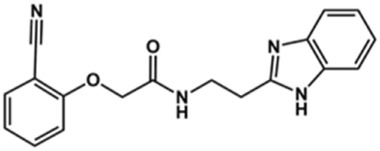	5.6	74.6	0.9	6.6	87.9	2.6	2.2
**32**	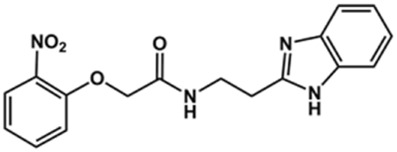	25.5	526.0	0.7	39.2	808.0	3.6	4.9
**33**	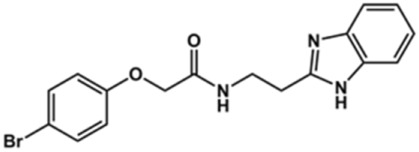	10.4	161.0	0.8	12.5	193.3	2.8	0.7
**34**	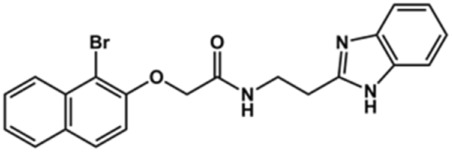	49.9	607.0	0.1	539.5	6562.2	4.4	0.3
**35**	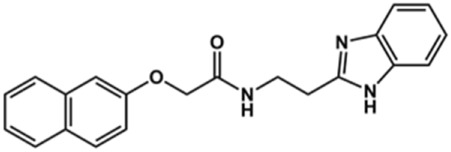	21.1	565.0	0.5	43.9	1174.6	3.9	0.7
**36**	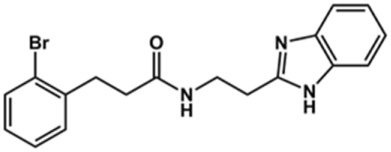	28.8	705.0	0.8	38.2	935.0	3.8	4.7
**37**	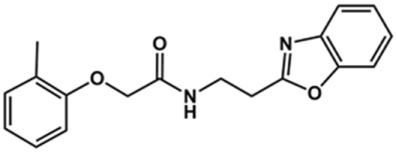	22.9	745.0	0.9	26.9	874.4	3.9	19.7
**38**	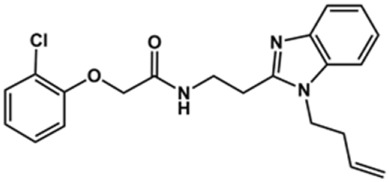	230.0	934.0	0.5	501.1	2034.9	4.1	7.7

CL_int_, intrinsic clearance after incubation with human and mouse microsomes; *fu*, fraction unbound; CL_int_u_, corrected clearance (CL_int_/fu); eLog*D*, experimentally determined distribution coefficient; PAMPA, parallel artificial membrane permeability assay.

Next, the same set of molecules was evaluated for their *in vivo* PK profile (Table 6). From this assay, information such as T_1/2_, plasma clearance (CL_p_), and bioavailability (F) were obtained. As observed for the *in vitro* assays, all benzimidazoles had high unbound clearance compared to that of BZ. The *in vivo* assays reinforced the concept that the high clearance may be the reason for the very low oral bioavailability (F) observed for the benzimidazoles (0–35%) compared to that of BZ (90%).

**TABLE 6:** *In vivo* PK profiles of selected benzimidazole derivatives.

IV								PO				
Cpd	T_1/2_(h)	C_0_ (ng/ml)	V SS (L/kg)	AUC (ng*h/ml)	CLp (L/h/kg)	*fu*	CLp_u_ (L/h/kg)	T_1/2_ (h)	C_max_ (ng/ml)	T_max_ (h)	AUC (ng*h/ml)	F (%)
BZ	0.8	961	1.1	1.020	1.0	0.99	1.0	1.5	404	0,4	1.040	90
**17**	0.5	153	4.4	83	6.3	0.06	104.3	—	0.0	—	0.0	0.0
**18**	0.2	283	1.8	84	3.2	0.03	103.7	—	—	0.3	1.0	1.2
**31**	0.2	232	1.9	67	4.6	0.15	29.9	—	12.8	0.3	—	—
**32**	0.2	220	2.5	53	5.9	0.04	157.4	—	—	0.3	1.0	1.9
**33**	0.2	658	0.6	160	9.5	0.16	60.6	0.4	71.7	0.3	56.5	35.2
**34**	0.3	255	1.9	116	7.6	0.00	2,874.7	0.6	7.6	0.3	6.5	5.6
**35**	0.2	228	2.0	86	5.8	0.02	334.6	0.2	3.7	0.3	1.7	2.0
**36**	0.5	144	7.2	55	9.5	0.04	231.5	—	0.0	—	0.0	0.0
**37**	0.2	169	2.4	53	9.5	0.05	187.1	—	14.6	0.3	—	—
**38**	0.4	126	3.3	82	6.2	0.01	430.5	—	2.5	0.3	—	—

IV, intravenous administration; PO, oral administration; T_1/2_, plasma half-life; C_0_, concentration at time = 0; VSS, steady-state volume of distribution; AUC, area under the curve; CLp, plasma clearance; *fu*, fraction unbound; CLp_u_, plasma clearance corrected for the fraction unbound; C_max_, peak plasma concentration; T_max_, time of peak plasma concentration; F, bioavailability.

**FIGURE 5:**
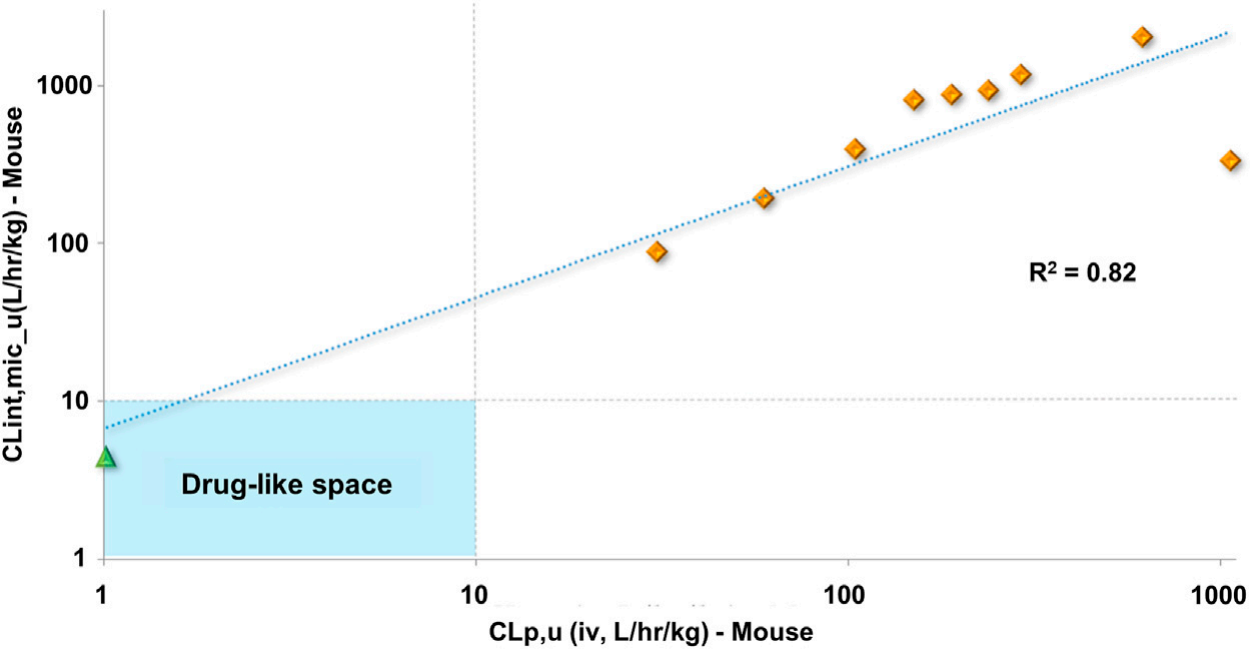
Clearance *in vitro-in vivo* correlation (IVIVC) plot showing a good correlation between the metabolic stability data. Benznidazole (green triangle); benzimidazole analogs (orange diamonds).

*In vitro* experiments are faster and less expensive than *in vivo* assays. Accessing the *in vitro-in vivo* correlation (IVIVC) for metabolic stability is important to demonstrate whether one can rely on *in vitro* studies and keep the use of animals to a minimum for a series of molecules. The lack of IVIVC is also informative, indicating that other metabolic routes are likely to be responsible for the observed *in vivo* clearance. In our experiments, a positive IVIVC was observed for *fu*-corrected clearance (Figure 5), which allowed us to rely on *in vitro* assays for the prediction of *in vivo* metabolic stability and prioritize compounds for further studies.

### Determination of Metabolic Stability in Human Hepatocytes and Identification of CYP450 Isoforms

All compounds evaluated showed high clearance values, reaching 60–180% of mouse liver blood flow (5.4 L/h/kg), which is a plausible explanation for their low bioavailability (0–35%). To better understand pathways involved in the elimination of the compounds, metabolic stability studies in human hepatocytes, which contain the whole set of human phase I and phase II hepatic metabolizing enzymes, were conducted. This experiment was performed in the absence of CYP inhibitors for the determination of the total clearance (Phase I + Phase II); in the presence of 1-ABT (a CYP450 inhibitor) for the determination of the fraction of the compounds that are metabolized by the remaining Phase I as well as the conjugating Phase II enzymes; and in the presence of azamulin (a CYP3A4 inhibitor) for the identification of the fraction metabolized by this isoform. This last assay provides critical information for prioritizing compounds since CYP3A4 plays a key role in drug-drug interactions (DDIs) and is associated with adverse effects and low efficacy when two or more drugs are taken together. Phase I metabolism, performed mainly by the CYP450 family, was responsible for 56–95% of the metabolism of the compounds (mean = 78.1 ± 12.9%). A much lower contribution to total clearance was observed for all other Phase I enzymes, which were responsible for 5–35% of the metabolism (mean = 21.9 ± 13.6%). The central role played by CYP3A4 became apparent when the CYP3A4 inhibitor azamulin was used in the assay: the resulting clearance values were approximately 40% lower, reaching a minimum value of 31.6% and a maximum value of 59.5% when compared with total clearance values (Supplementary Figure S1).

After identifying the central role of CYP3A4 in the metabolism of this series, the next step was to identify the involved isoforms using recombinant CYP enzymes. CL_int_ was determined based on the residual amount of the compound over time. Additionally, the contribution of each CYP isoform to metabolism was calculated based on their relative abundance in humans. The information generated by this experiment was essential to assess the risk of potential drug-drug interactions (DDIs) for this series of compounds. Molecules eliminated through multiple pathways have reduced DDI potential and are therefore more suitable for advancing to further steps in a drug discovery pipeline. The benzimidazole derivatives are mainly metabolized by CYP3A4 (23–90%) (Supplementary Table S4). Although modest, a contribution from isoforms CYP2D6 and CYP1A2 is observed, featuring an attractive profile from a DDI perspective despite the high clearance values.

### Identification of Sites of Metabolism

All studies on benzimidazoles showed that these molecules are metabolically unstable, and biotransformation mediated by CYP3A4 is the major metabolic route. Therefore, studies to identify the molecular sites of metabolism (SOM) were performed. These studies can enable the blockage of these sites by the inclusion of blocking groups to achieve appropriate levels of metabolic stability. Ideally, these molecular changes should not significantly affect the potency toward the molecular target. The test compounds were then incubated with human and mouse liver microsomes. Most of the metabolites were found to be oxidation products mainly of the linker and benzimidazole moieties (Supplementary Figures S2–S5). Strategies to block these SOMs could include switching the amide position and adding halogen atoms to the linker. At the benzimidazole ring, *N* substitutions and the addition of halogens could be explored (Supplementary Figure S6).

### Metabolic Stability Studies for an Additional Set of Benzimidazole Derivatives

After completing the PK profile for 10 molecules (set 1), an additional set of 55 compounds (set 2) was evaluated to provide additional information for the establishment of a SAR for metabolic stability. The results for clearance after incubation with human and mouse liver microsomes, eLog*D*, and PAMPA are summarized in Supplementary Table S5. Some set 2 compounds with lower clearance values than those of set 1 molecules were identified, some of which exhibited clearance values comparable to that of BZ (Supplementary Figure S7).

The clearance values listed in Supplementary Table S5 show that the presence of substituents on the phenyl ring might influence the metabolic stability of the compounds. Substituents at *para* and *meta*, for example, led to the most stable compounds with the lowest CL_int__u values. Among the compounds with substituents at *para*, compounds **2**, **4** and **5** are highlighted, for which clearance values are in the same range of BZ (CL_int__u = 1.50 L/h/kg). In addition, compounds **6** (CL_int__u = 3.54 L/h/kg), **7** (CL_int__u = 1.50 L/h/kg), and **3** (CL_int__u = 6.90 L/h/kg), with substituents at *meta,* showed drug-like profiles for metabolic stability. Substituents at *ortho* also led to stable compounds: **31** (CL_int__u = 6.6 L/h/kg), **42** (CL_int__u = 4.64 L/h/kg), and **43** (CL_int__u = 3.94 L/h/kg).

**FIGURE 6:**
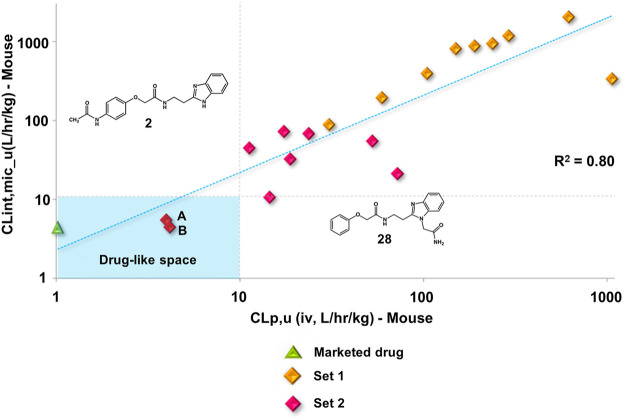
Clearance *in vitro-in vivo* correlation (IVIVC) plot showing a good correlation between the metabolic stability data. Benznidazole (green triangle); set 1 compounds (orange diamonds); set 2 compounds (red diamonds). Compounds **2 (A)** and **28 (B)**.

Set 2 compounds did not show significant structural variability in the linker region. Among the few exceptions are compounds **48**, in which sulfur replaced the linker oxygen (CL_int__u = 11.38 L/h/kg), and **58** (CL_int__u = 16.99 L/h/kg) and **49** (CL_int__u = 3.28 L/h/kg), in which the position of the phenoxy fragment was modified by the introduction of a methylene group at the linker. At the benzimidazole ring, the introduction of a hydrophilic amide led to high metabolic stability (**28**, CL_int__u = 1.53 L/h/kg). It is important to highlight the influence exerted by the physicochemical nature of the substituents at the phenyl and the benzimidazole on the stability of the compounds (Supplementary Figure S8). The introduction of hydrophobic substituents at the phenyl resulted in high clearance values, such as those observed for compounds **44** (CL_int__u = 70.62 L/h/kg), **51** (CL_int__u = 65.56 L/h/kg), **52** (CL_int__u = 2,122.45 L/h/kg), and **60** (CL_int__u = 194.48 L/h/kg). Hydrophobic substituents at the benzimidazole (**19**, **20**, **21**, **23**, **26**, **27**, and **30**) followed the same trend, with CL_int__u values ranging from 70.23 to 3,366.34 L/h/kg. Overall, the clearance values for the benzimidazole derivatives increased with increasing hydrophobicity (Supplementary Figure S9). Seven set 2 compounds (**1**, **2**, **3**, **8**, **10**, **11**, and **28**) with suitable trypanocidal activity and *in vitro* clearance underwent *in vivo* PK studies. Overall, the set 2 compounds exhibited lower CLp_u values compared to those of the set 1 analogs (Supplementary Table S6), with benzimidazoles **2** and **28** showing the most promising profiles (CLp_u of 4.16 and 3.98, respectively). Additionally, similar to the profile observed for the set 1 compounds, a good correlation between *in vitro* and *in vivo* clearance was found for the set 2 benzimidazoles (Figure 6).

### *In Vivo* Toxicity and Trypanocidal Activity

**FIGURE 7:**
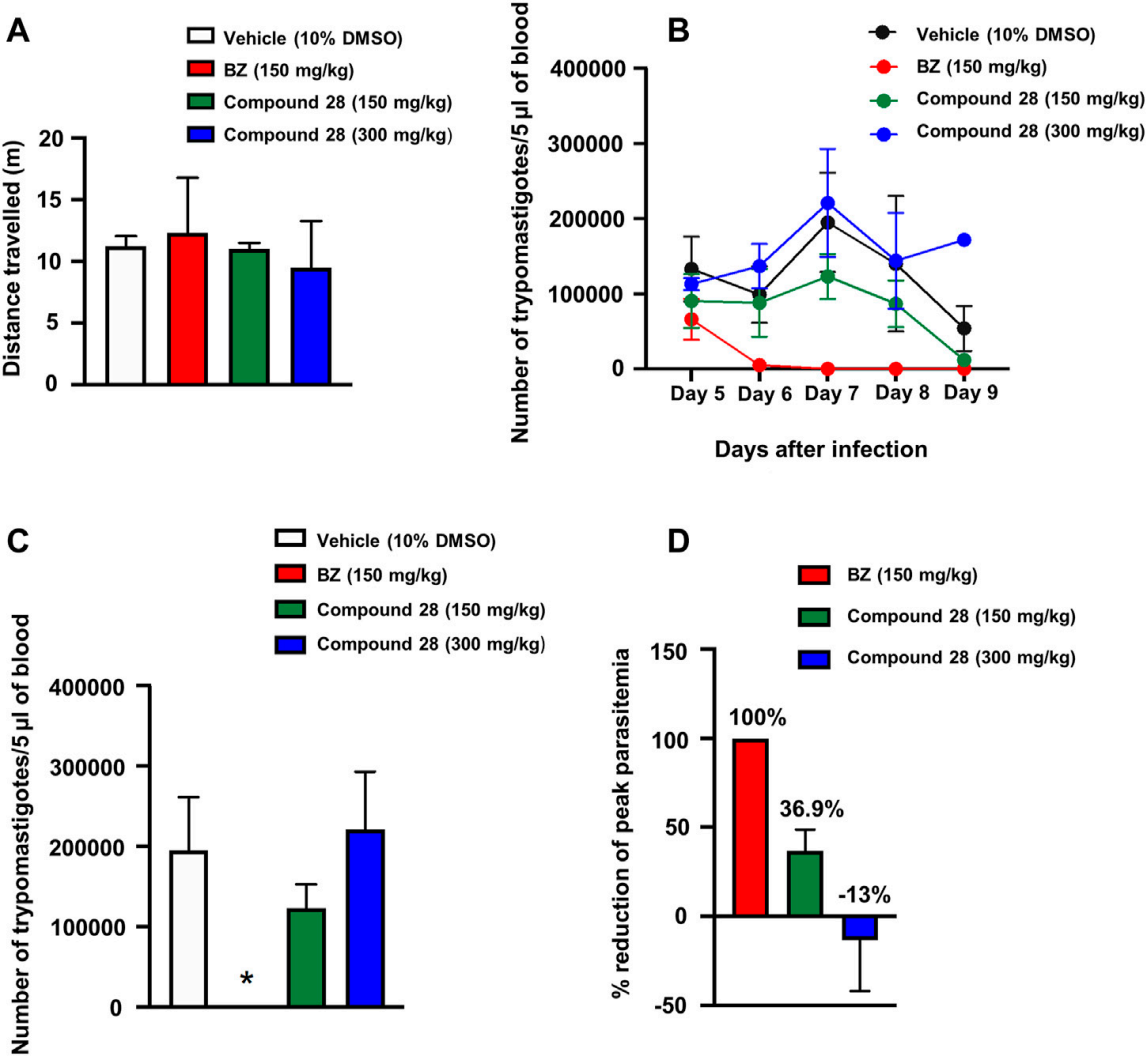
Acute toxicity and trypanocidal activity *in vivo*. **(A)** Open-field test. Mice were orally treated with vehicle (10% DMSO) or benznidazole (BZ) at doses of 150 mg/kg or **28** at doses of 150 and 300 mg/kg. **(B)** Parasitemia during *T. cruzi* infection in mice treated with vehicle, BZ or **28** (150 and 300 mg/kg) expressed as the number of trypomastigotes per 5 μl of blood. The data represent the mean parasitemia ± SEM (4–8 animals per group) for all assays. **(C)** Peak parasitemia expressed as the number of trypomastigotes per 5 μl of blood in mice treated with vehicle, BZ or **28** (150 and 300 mg/kg) (*p <0.05 when compared to vehicle and other groups). **(D)** Reduction of peak parasitemia (seventh day of infection) in mice treated with vehicle, BZ or **28** (150 and 300 mg/kg). Vehicle solution: 0.9% NaCl + 10% DMSO.

Compound **28** (IC_50_^*T. cruzi*^ = 6.8 µM) was selected for a proof-of-concept study given its suitable balance between pharmacodynamics and PK properties. Initially, we determined the doses that elicited no acute toxicity. The compound, solubilized in 10% DMSO aqueous solution, was administered orally in a single dose of 150 and 300 mg/kg of body weight in female Swiss mice. Parameters related to behavior, autonomic functions, neurological activity, and mortality were assessed as toxicity signs. Soon after the administration of the compound, mice were placed in a circular open-field arena (40 cm diameter) with 50-cm-high walls to assess motor deficits. Mortality and clinical signs associated with toxicity were recorded 0.5, 2, 4, 8, and 24 h after the single-dose administration. After this period, toxicity signs were assessed once a day for two consecutive weeks. The reference drug BZ at 150 mg/kg and vehicle (10% DMSO) were administered as controls. No toxicity signs were observed within the 2 weeks of observation for any of the tested doses. Additionally, no mortality was observed (Figure 7A). One-way ANOVA did not reveal any difference among the groups [F3,4 = 0.15; p = 0.9238], indicating that treatment with **28** at doses of 150 and 300 mg/kg did not cause locomotor deficits.

Considering the favorable acute toxicity results, the *in vivo* trypanocidal activity of **28** was determined at single doses of 150 and 300 mg/kg for 5 days. Female Swiss mice were infected with *T. cruzi* (Y strain) (Rodriguez et al., 2014) and treated via gavage with five daily doses of BZ (150 mg/kg of body weight), **28** (150 and 300 mg/kg of body weight) and vehicle (10% DMSO). Treatment started on day five after infection with *T. cruzi*. The following parameters were evaluated in these experiments: level of parasitemia after the treatment, suppression of peak parasitemia (day seven after the infection), and reduction of parasitemia on the peak day (day seven after infection). Parasitemia was expressed as the number of *T. cruzi* trypomastigotes per 5 µl of blood and was calculated using the Brener method (Brener, 1962). Repeated ANOVA measures considering the factors treatment and day (repeated measure) showed a main effect of treatment (F3,18 = 12.68; *p* < 0.05) (Figure 7B). On the seventh day of treatment, when parasitemia reached its peak, one-way ANOVA indicated a treatment effect (F3,16 = 7.47; *p* = 0.002). Post hoc analyses indicated that the treatment with BZ significantly decreased parasite burden compared with the other treatments (*p* < 0.05) (Figure 7C). BZ and **28** (150 mg/kg) reduced peak parasitemia by 100 and 36.9%, respectively, compared with the vehicle. At a dose of 300 mg/kg, benzimidazole **28** showed an increase of 13% in the peak parasitemia when compared with the vehicle (Figure 7D). This dose-response effect is likely associated with the modulation of physiological systems that increase the susceptibility of the animals to infection with *T. cruzi* at high doses of the compound*.* Further tests with lower doses (75 mg/kg and 37.5 mg/kg) showed no reduction in parasitemia levels (Supplementary Figure S10). The results of the *in vivo* studies indicate a moderate ability of **28** to suppress peak parasitemia at 150 mg/kg.

Considering that few molecular targets are validated in NTDs (De Rycker et al., 2018) and the relatively unsuitable compounds regarding toxicity and drug-likeness that have been historically explored in the area, the findings reported herein address an important gap in Chagas disease drug discovery. Regardless of the mechanism of action, it is noteworthy that in rare cases a compound succeeds in terms of efficacy in Chagas disease *in vivo* infection models. This is a major hurdle in the field that can be related to the complex life-cycle biology of *T. cruzi,* and the many poorly understood aspects of the interplay between the parasite and the host (Libisch et al., 2021). Among the compounds that reached this milestone, we can highlight vinyl sulfone K777, CYP51-inhibitor azoles (including posaconazole), and cruzain-inhibitor triazoles and carbamoyl imidazoles (Ferraz et al., 2007; McKerrow et al., 2009; Brak et al., 2010; de Souza et al., 2020). K777 was a landmark in the field as it was the first compound to show the possibility to enter clinical trials for Chagas disease. However, tolerability issues in dogs and primates during the preclinical phase hampered the progression of this compound toward clinical development. After the failure of K777, it was discussed whether the toxicity issues would be due to the irreversible mechanism of action and resulting lack of selectivity of K777 over other proteases, which could include human proteases. The case of K777 highlights the importance of designing reversible cruzain inhibitors with improved selectivity as are the benzimidazole derivatives investigated in this work. In this study, we adopted the strategy of diversifying the substitution pattern at the phenyl and benzimidazole regions. This approach led to an enhanced interaction with cruzain and, by enabling the formation of a hydrogen bond with Glu208, it improves the selectivity for cruzain over other proteases. Glu208 is part of the S2 subsite in the cruzain active site and is lacking in most other proteases such as human cathepsins. The role played by the formation of a hydrogen bond with Glu208 was investigated by evaluating a set of compounds against rhodesain, a cysteine protease that has an active site that resemble that of cruzain in which an alanine replaces Glu208. The compounds were far more active against cruzain over rhodesain, which indicates the important part played by Glu208 in selectivity toward cruzain.

Another key finding reported in Chagas disease drug design was the identification of CYP51-inhibitor antifungal azoles (Ferraz et al., 2007). These compounds, particularly Posaconazole and E1224 (the ravuconazole prodrug), showed promising suppressive effects in parasite burden in animal models of Chagas disease. However, their failure in clinical trials raised fruitful discussions regarding the mechanism of action of the compounds. Although these azoles displayed a remarkable suppressive effect, they failed in providing sustained parasite clearance when opposed to benznidazole. These studies served to establish the landmark that *T. cruzi* CYP51 is not a molecular target to be pursued in Chagas disease drug discovery. These previous findings demonstrate the critical importance of target validation and identification of compounds that act by different modes of action, for example, the modulation of cruzain. The compounds studied herein showed a moderate reduction of parasite burden and, therefore, open novel possibilities for future work on this molecular target. Moreover, the benzimidazoles did not demonstrate toxicity in animal studies, which, as seen in the K777 case, can be an issue of cysteine protease inhibitors. The best compound (**28**) administered orally to mice in a single dose of 150 and 300 mg/kg showed no toxicity signs for any of the doses and, importantly, no mortality was observed.

Another important class of compounds is triazole-based cruzain inhibitors, whose representative analogs showed promising *in vivo* efficacy (Brak et al., 2010; Neitz et al., 2015). These non-peptidic ketones irreversibly inactivate cruzain by attaching covalently to Cys25, which can raise selectivity issues and, therefore, be a drawback for further development. Regarding the PK profile, optimization of these triazoles resulted in enhanced bioavailability and exposure after oral dosing, although they proved to inhibit CYP3A4, the most important CYP isoform for the elimination of xenobiotics. The best compound identified herein (**28**), showed a suitable tradeoff among pharmacodynamics and PK properties. Regarding its mechanism of action, inhibitor **28** is a reversible inhibitor and interacts with Glu208, which reduces the probability of inhibition of human proteases. Additionally, the extensive PK studies enabled the identification of permeable, metabolically stable, and bioavailable compounds with high selectivity indices, and that are metabolized mainly by CYP3A4. Incubation of the compounds with isolated recombinant CYPs using CYP3A4 and pan-CYP inhibitors as controls showed that the benzimidazoles do not inhibit CYP3A4. These findings are pivotal in the context of drug-drug interactions, particularly in the case of chagasic patients who need to use different drugs to mitigate the complications of the disease.

## Conclusion

An MPO strategy for the optimization of benzimidazole derivatives as antichagasic agents was developed. This strategy relied on the parallel optimization of activity against cruzain and *T. cruzi*, selectivity, and PK parameters such as metabolic stability and permeability. New compounds were synthesized, and previously synthesized analogs were thoroughly evaluated for PK properties. Newly introduced *N*-substituents at the benzimidazole ring revealed that increasing bulkiness at this site modifies the mechanism of action toward cruzain from competitive to noncompetitive. These results introduce new and interesting aspects regarding the binding mode and mechanism of action of cruzain inhibitors. Newly designed phenyl-substituted analogs showed increased inhibition of cruzain over rhodesain, demonstrating the key role played by Glu208 in the selective inhibition of cruzain over other proteases. Some of the benzimidazole derivatives showed appropriate metabolic stability and clearance values comparable to those of drug-like molecules. Phase I oxidation reactions catalyzed by CYP3A4 were detected as the main elimination pathway, and the identified sites of metabolism provided insights into the improvement of metabolic stability. Moreover, the analysis of the *in vitro* trypanocidal and cytotoxicity data revealed a sound selectivity index for the investigated compounds, indicating a low potential for toxicity.

The applied MPO approach enabled the prioritization of compounds considering an appropriate combination of *in vitro* activity, toxicity, and PK properties. The gathered *in vitro* data supported *in vivo* PK studies for representative compounds. A solid IVIVC was obtained, demonstrating the high predictive ability of the *in vitro* PK models for the corresponding *in vivo* endpoints. Finally, acute toxicity and efficacy studies were conducted for compound **28**, which showed no toxicity signs and a moderate reduction in peak parasitemia at 150 mg/kg. Importantly, the knowledge gathered in this study opens novel opportunities to understand the molecular aspects of cruzain inhibition, enabling the discovery of compounds with a good trade-off between pharmacodynamics and pharmacokinetics.

## Data Availability

The original contributions presented in the study are included in the article/Supplementary Material, further inquiries can be directed to the corresponding authors.

## Footnotes

1
https://www.who.int/news-room/fact-sheets/detail/chagas-disease-(american-trypanosomiasis).

2
https://www.paho.org/en/topics/chagas-disease.
