# Supplementary material for: Multiparameter Optimization of Trypanocidal Cruzain Inhibitors With In Vivo Activity and Favorable Pharmacokinetics
Source: Front Pharmacol. 2022 Jan 5;12:774069. doi: 10.3389/fphar.2021.774069 (PMC8767159; doi:10.3389/fphar.2021.774069)
Supplement: Supplementary file 1 [file DataSheet1.pdf]

SUPPORTING INFORMATION

Title

Multiparameter Optimization of Trypanocidal Cruzain Inhibitors with *In Vivo* Activity and Favorable Pharmacokinetics

Authors

Ivani Pauli,[a*] Celso O. Rezende Jr.,[b*] Brian W. Slafer,[b] Marco A. Dessoy,[b] Mariana L. de Souza,[a] Leonardo L. G. Ferreira,[a] Abraham L. M. Adjanohun,[c] Rafaela S. Ferreira,[c] Luma G. Magalhães,[a] Renata Krogh,[a] Simone Michelan-Duarte,[a] Ricardo Vaz Del Pintor,[d] Fernando B. R. da Silva,[d] Fabio C. Cruz,[d] Luiz C. Dias,[b**], Adriano D. Andricopulo[a**]

Affiliations

[a]Laboratório de Química Medicinal e Computacional, Instituto de Física de São Carlos, Universidade de São Paulo, Avenida João Dagnone 1100, São Carlos, SP 13563-120, Brazil.[b] Instituto de Química, Universidade Estadual de Campinas, 6154, Campinas, SP 13084-971, Brazil.[c] Departamento de Bioquímica e Imunologia, Universidade Federal de Minas Gerais, Avenida Antônio Carlos 6627, Belo Horizonte, MG 31270-901, Brazil. [d] Departamento de Farmacologia, Universidade Federal de São Paulo, Rua Botucatu 862, São Paulo – SP 04023-062, Brazil.

**Correspondence

Adriano D. Andricopulo: aandrico@ifsc.usp.br

Luiz Carlos Dias: ldias@unicamp.br

*These authors contributed equally to this work

Supporting Figures

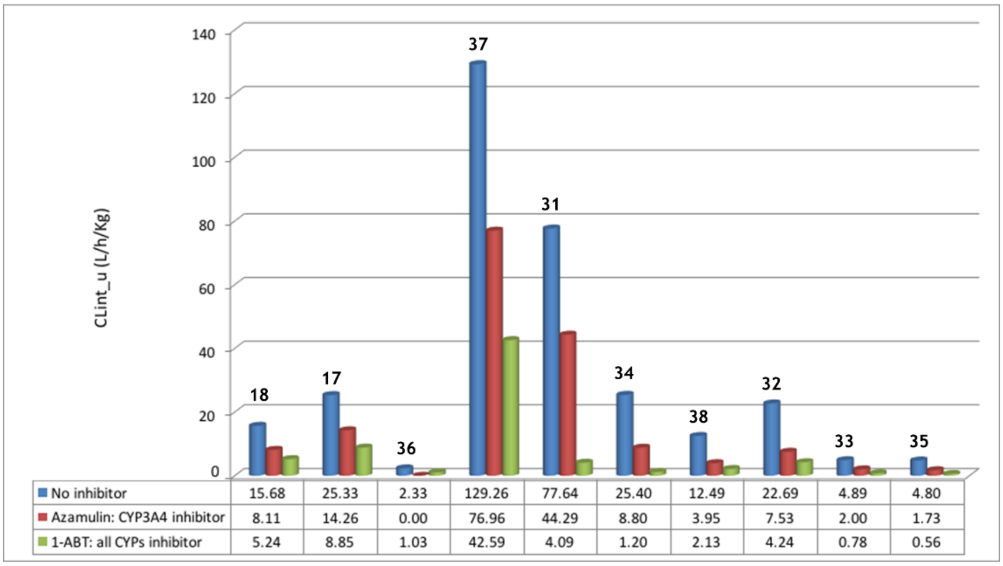

Figure S1. Clearance of compounds evaluated after incubation with human hepatocytes, represented as clearance values. Blue bars correspond to total clearance. Residual clearance after incubation with azamulin is shown as red bars. Residual clearance after incubation with 1-ABT is represented as green bars.

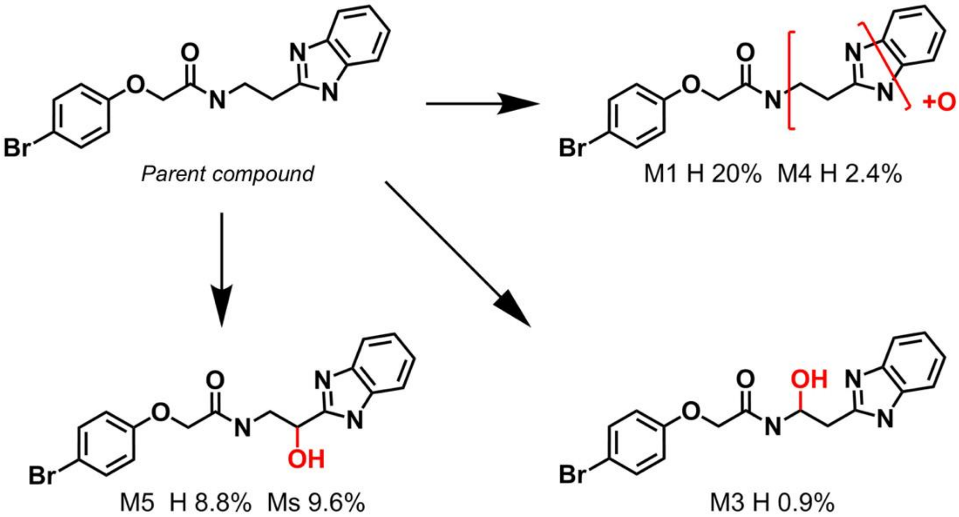

Figure S2. Biotransformation of compound **38**. **M**: metabolite, **H**: human, **Ms**: mouse.

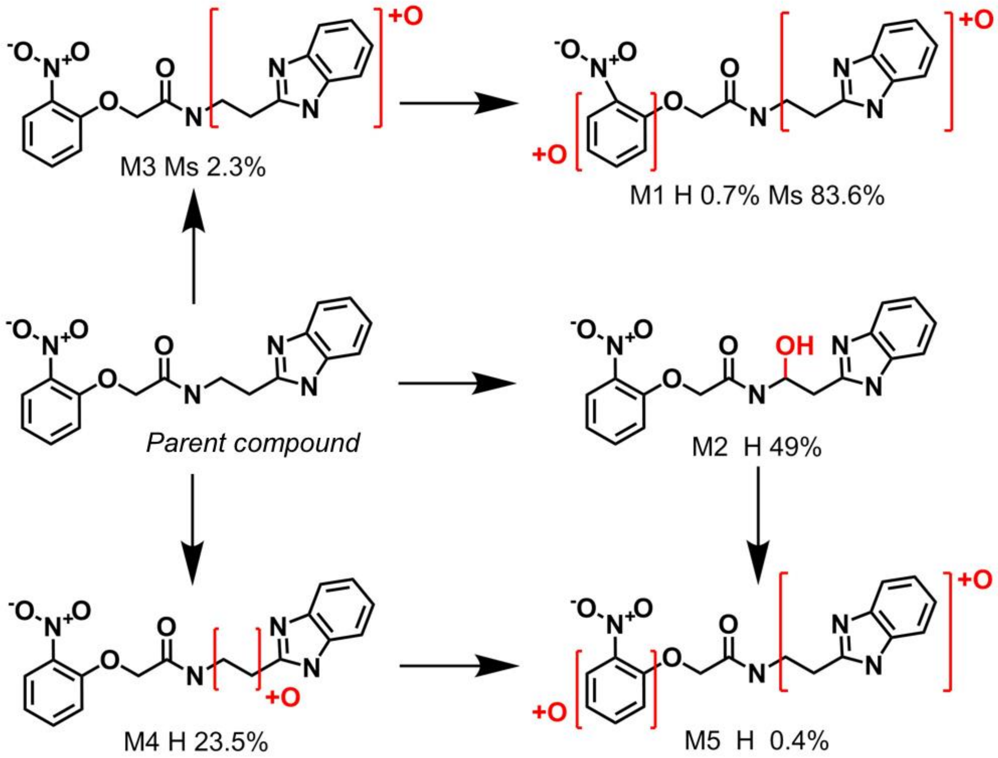

Figure S3. Biotransformation of compound **18**. **M**: metabolite, **H**: human, **Ms**: mouse.

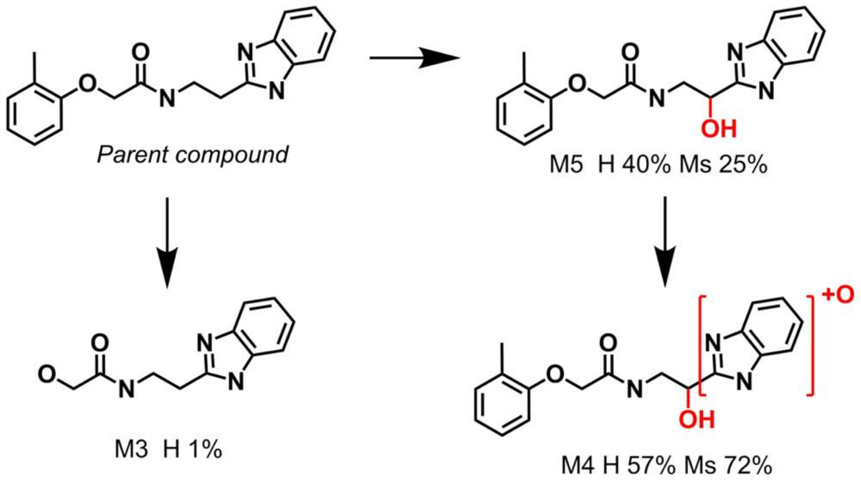

Figure S4. Biotransformation of compound **33**. **M**: metabolite, **H**: human, **Ms**: mouse.

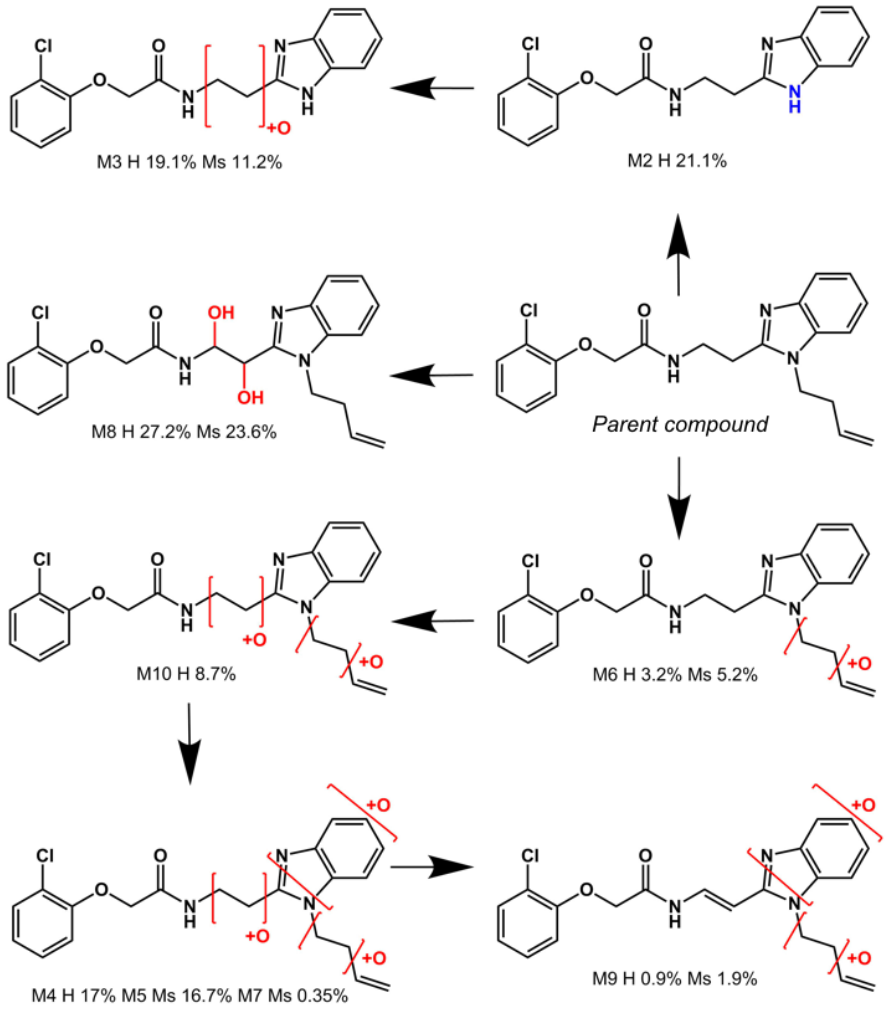

Figure S5. Biotransformation of compound **17**. **M**: metabolite, **H**: human, **Ms**: mouse.

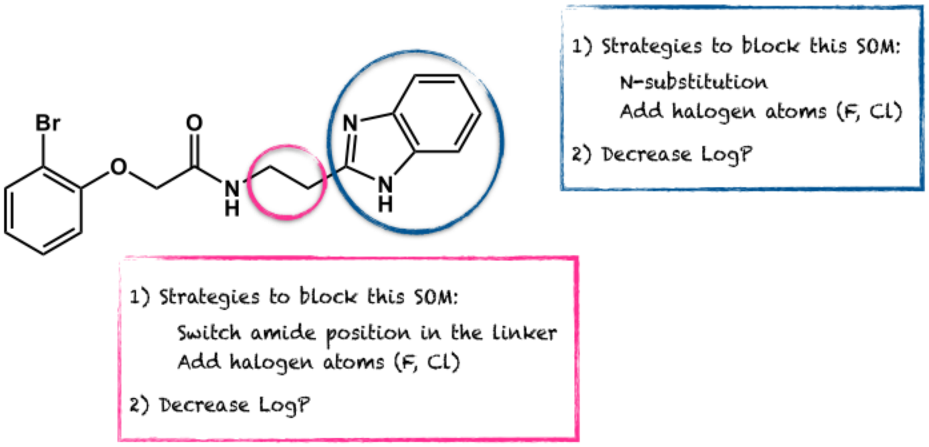

Figure S6. Strategies to block the identified SOMs**.**

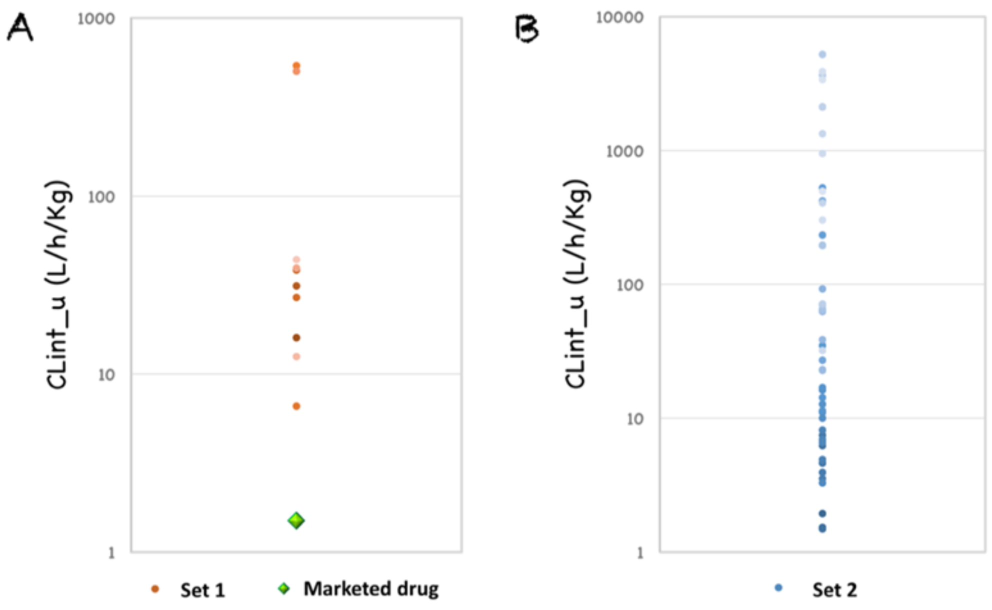

Figure S7. Graphical representation of intrinsic clearance values obtained by the incubation of the compounds with human liver microsomesµ considering unbound fraction as a correction factor. This figure allows a comparison of the values for sets 1 and 2, and the drug **BZ**.

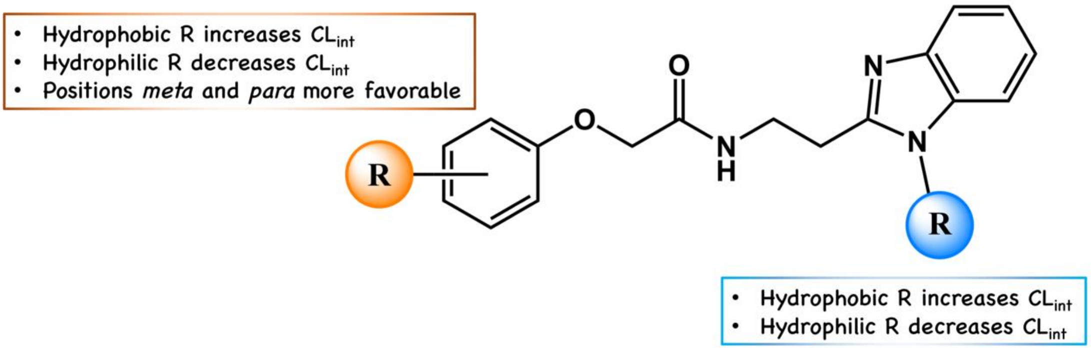

Figure S8. SAR scheme for the influence of hydrophobicity of substituents at the phenyl and benzimidazole and *fu*-corrected intrinsic clearance (CL_{int}_u).

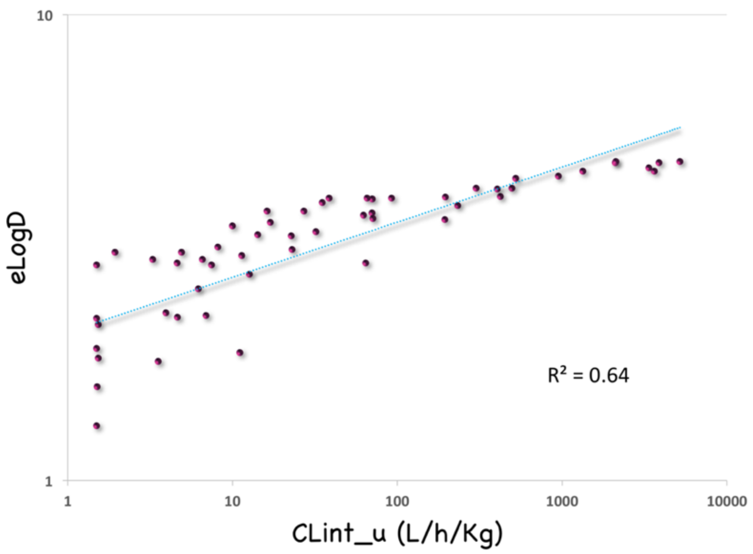

Figure S9. Correlation between *fu*-corrected intrinsic clearance (CL_{int}_u) after incubation with human liver microsomes and hydrophobicity of the compounds represented by the coefficient of distribution (eLog*D*).

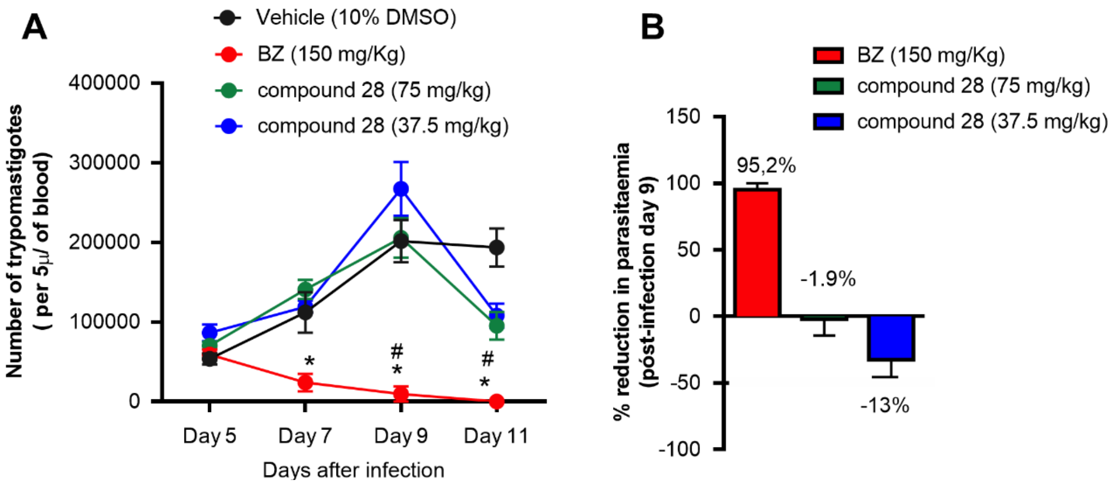

Figure S10. Reduction of parasite burden *in vivo* after treatment with **BZ**, Compound **28** (75 and 37.5 mg/kg). (A) Parasitemia during *T. cruzi* (Y strain) infection in mice treated with vehicle, benznidazole (**BZ**) or Compound **28** (75 and 37.5 mg/kg), expressed as the number of trypomastigotes per 5 µL of blood. Treatment started on day five after infection. (B) Percentage of reduction in parasitemia on the peak day of infection (post-infection day 9) in mice treated with vehicle, **BZ**, or Compound **28** (75 and 37.5 mg/kg). The data represent the mean ± SEM (5 animals per group). Vehicle solution: 0.9% NaCl + 10% DMSO. *p<0.05 when compared to all doses of the compound **28**; #p<0.05 when compared to vehicle.

Supporting Tables

Table S1. Cruzain inhibition sensitivity assay to Triton X100.[a]

Compound	0% Triton	0.01% Triton	0.1% Triton
**19**	73.4 ± 2.5	72.1 ± 1.7	77.5 ± 2.2
**20**	95.2 ± 0.6	95.9 ± 1.7	96.0 ± 1.1
**21**	80.3 ± 1.4	85.5 ± 2.3	84.6 ± 1.3
**23**	52.1 ± 1.3	53.2 ± 1.9	55.5 ± 5.2
**24**	87.9 ± 0.7	82.6 ± 0.3	82.9 ± 3.5

[a]The % of inhibition values correspond to the mean of three measures.

Table S2. Confirmation of the non-competitive mechanism of action of compounds **20**, **24**, **26**, and **27**.[a]

[S] (μM)/IC_{50} (μM)[a]	20	24	26	27
**2.5**	0.5 ± 1.3	1.7 ± 1.3	1.6 ± 0.1	12.4 ± 2.2
**5**	0.4 ± 1.2	2.2 ± 1.3	1.8 ± 0.1	7.5 ± 1.2
**10**	1.0 ± 1.5	2.2 ± 1.1	1.9 ± 0.2	14.7 ± 1.8
**15**	0.8 ± 1.7	1.9 ± 1.8	1.4 ± 0.5	11.2 ± 1.5
**20**	0.3 ± 1.5	2.5± 1.2	2.4 ± 0.9	10.2 ± 1.5
**40**	0.5 ± 1.2	2.1± 0.9	1.2 ± 0.5	8.9 ± 3.1

[a] IC_{50} values were determined independently by the measured triplicate for at least six inhibitor concentrations. At least two independent assays were run.

Table S3. Inhibitory properties of benzimidazole derivatives with substituents in the phenyl ring against cruzain and rhodesain.[a]

Compound	% Cruzain inhibition (100 μM)[a]	% Rhodesain inhibition (100 μM)[a]
**10**	90	60
**3**	70	41
**11**	96	67
**12**	100	56
**13**	89	54
**9**	90	40

[a] The percentage of inhibition values shown in this table refer to the mean of three experimental measures.

Table S4. CL_{int_u} (L/h/kg) values and fraction metabolized (%) for each evaluated CYP450 isoform using recombinant enzymes.

Compound	Structure	CYP3A4	CYP2D6	CYP1A2	CYP2C9	CYP2C19	Fraction %
18	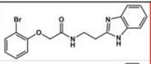	9.67 L/h/Kg 58.51 %	1.78 L/h/Kg 10.77 %	4.30 L/h/Kg 26.02 %	0.36 L/h/Kg 2.15 %	0.42 L/h/Kg 2.56 %	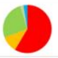
17	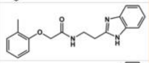	14.40 L/h/Kg 48.25 %	9.69 L/h/Kg 32.47 %	3.94 L/h/Kg 13.20 %	1.01 L/h/Kg 3.38 %	0.76 L/h/Kg 2.54 %	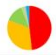
36	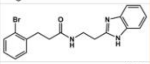	53.00 L/h/Kg 88.42 %	2.76 L/h/Kg 4.60 %	0.30 L/h/Kg 0.50 %	1.24 L/h/Kg 2.07 %	2.62 L/h/Kg 4.37 %	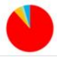
37	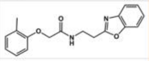	6.47 L/h/Kg 23.75 %	0.44 L/h/Kg 1.60 %	16.40 L/h/Kg 60.21 %	1.38 L/h/Kg 5.07 %	2.53 L/h/Kg 9.29 %	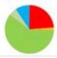
31	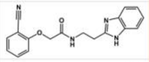	5.71 L/h/Kg 75.85 %	1.40 L/h/Kg 18.60 %	<0.04 L/h/Kg 0.00 %	< 0.11 L/h/Kg 0.00 %	0.42 L/h/Kg 5.55 %	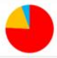
34	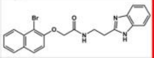	24.50 L/h/Kg 68.72 %	4.18 L/h/Kg 11.72 %	2.29 L/h/Kg 6.42 %	4.04 L/h/Kg 11.33 %	0.61 L/h/Kg 1.70 %	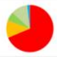
38	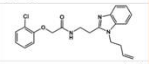	53.30 L/h/Kg 84.20 %	5.67 L/h/Kg 8.96 %	0.39 L/h/Kg 0.62 %	0.95 L/h/Kg 1.51 %	2.93 L/h/Kg 4.63 %	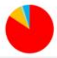
32	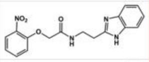	22.5 L/h/Kg 62.45 %	7.10 L/h/Kg 19.71 %	2.11 L/h/Kg 5.86 %	1.78 L/h/Kg 4.94 %	2.54 L/h/Kg 7.05 %	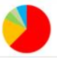
33	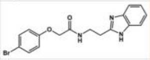	16.6 L/h/Kg 90.80 %	0.56 L/h/Kg 3.04 %	0.13 L/h/Kg 0.72 %	< 0.11 L/h/Kg 0.00 %	1.00 L/h/Kg 5.44 %	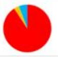
35	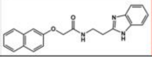	21.60 L/h/Kg 70.87 %	5.06 L/h/Kg 16.60 %	3.54 L/h/Kg 11.61 %	< 0.11 L/h/Kg 0.00 %	1.00 L/h/Kg 5.44 %	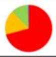

Table S5. In *vitro* PK profile of the set 2 benzimidazole analogs.

Compound	Structure	CLint (L/h/kg) Human	CLint (L/h/kg) Mouse	*fu*	CLint_u (L/h/kg) Human	CLint_u (L/h/kg) Mouse	eLog*D*	PAMPA
**BZ**	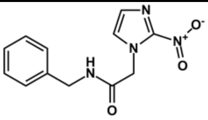	1.50	4.43	1.00	1.50	4.43	0.84	3.17
**39**	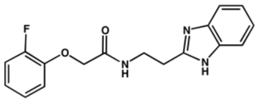	7.03	125.00	0.94	7.45	132.56	2.90	1.61
**40**	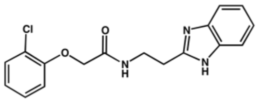	19.50	ND	0.86	22.70	419.09	3.35	3.74
**41**	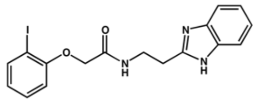	39.40	683.00	0.63	62.44	1,082.41	3.71	2.00
**42**	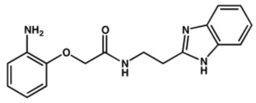	3.91	24.80	0.84	4.64	29.45	2.24	0.58
**43**	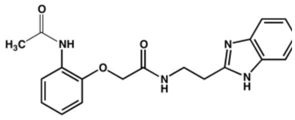	3.94	10.30	1.00	3.94	10.30	2.29	0.69
**1**	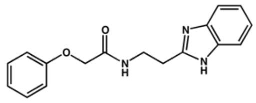	<1.50	72.10	1.00	1.50	72.10	2.90	4.21

**44**	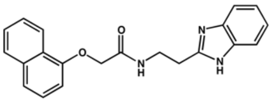	23.80	504.00	0.34	70.62	1,495.55	4.02	2.35
**45**	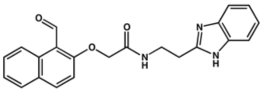	37.30	669.00	0.52	71.18	1,276.72	3.65	0.62
**46**	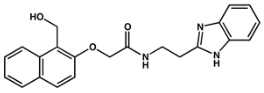	24.70	757.00	0.77	32.04	981.84	3.42	0.29
**47**	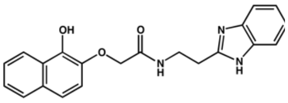	ND	655.00	0.72	35.00	939.71	3.95	0.33
**48**	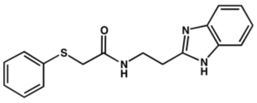	9.07	234.00	0.80	11.38	293.60	3.04	2.90
**49**	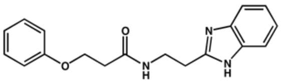	3.28	86.20	1.00	3.28	86.20	2.98	1.80
**50**	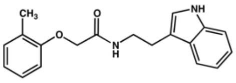	23.60	790.00	0.61	38.63	1,292.96	4.04	12.70
**51**	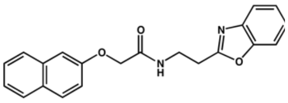	35.40	618.00	0.54	65.56	1,144.44	4.04	16.00
**52**	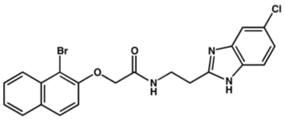	41.60	407.00	0.02	2,122.4	20,765.31	4.84	0.12
**53**	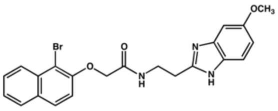	72.40	500.00	0.18	404.47	2,793.30	4.22	0.29
**54**	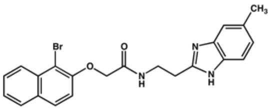	65.00	528.00	0.05	1,334.7	10,841.89	4.61	0.09
**19**	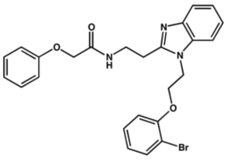	112.00	305.00	0.12	949.15	2,584.75	4.50	0.08
**20**	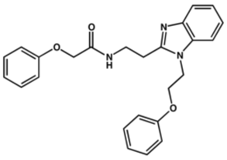	123.00	489.00	0.41	301.47	1,198.53	4.24	4.30
**21**	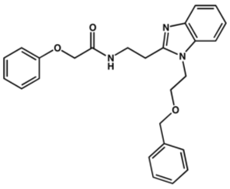	235.00	847.00	0.47	495.78	1,786.92	4.24	3.98

**22**	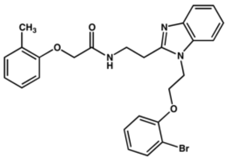	123.00	487.00	0.03	3,867.9	15,314.17	4.81	0.04
**23**	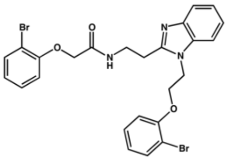	136.00	300.00	0.04	3,366.3	7,425.74	4.69	0.08
**24**	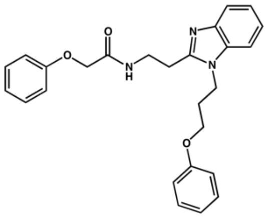	142.00	500.94	0.27	524.02	1,848.62	4.45	2.29
**25**	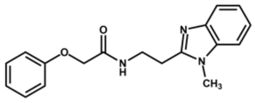	4.57	122.00	0.99	4.61	123.11	2.93	6.55
**26**	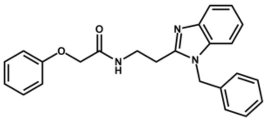	120.00	620.00	0.61	196.08	1,013.07	4.06	8.72
**27**	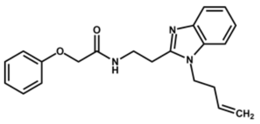	60.40	650.00	0.86	70.23	755.81	3.75	9.06
**28**	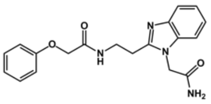	<1.50	5.34	0.98	1.53	5.43	2.16	0.71
**30**	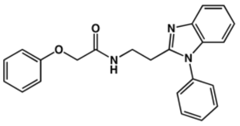	58.10	713.00	0.63	92.37	1,133.55	4.04	17.8
**29**	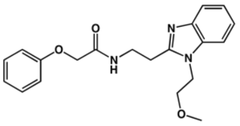	23.00	235.00	1.00	23.00	235.00	3.13	9.35
**2**	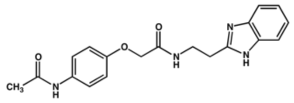	<1.50	<4.43	1.00	1.50	4.43	1.92	0.21
**4**	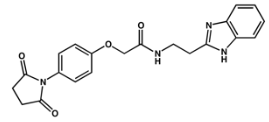	<1.50	20.80	0.99	1.51	20.88	1.59	0.17
**6**	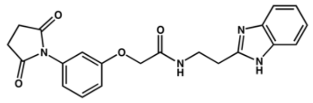	3.54	217.00	1.00	3.54	217.00	1.80	0.20

**7**	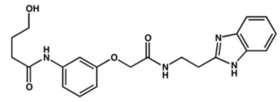	<1.50	8.19	1.00	1.50	8.19	2.23	0.03
**5**	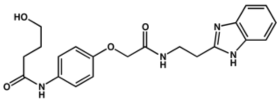	1.50	4.43	0.96	1.53	4.51	1.83	0.02
**10**	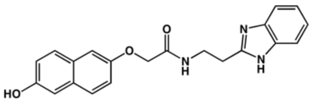	6.66	26.40	0.82	8.15	32.31	3.17	0.21
**3**	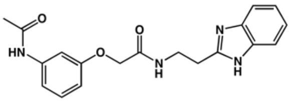	6.90	10.60	1.00	6.90	10.60	2.26	0.17
**11**	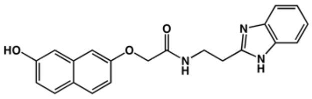	9.48	45.70	0.67	14.21	68.52	3.37	0.13
**12**	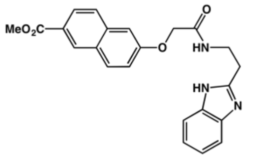	25.60	177.00	ND	232.73	1,609.09	3.89	0.34
**8**	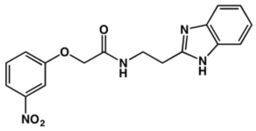	6.58	67.50	1.00	6.58	67.50	2.98	2.17
**9**	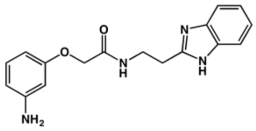	11.00	22.70	0.99	11.07	22.84	1.88	0.38
**15**	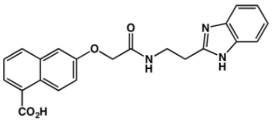	<1.50	12.90	1.00	1.50	12.90	1.31	ND
**55**	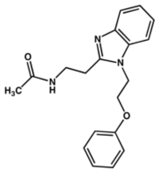	1.78	35.90	0.91	1.94	39.06	3.09	5.46
**56**	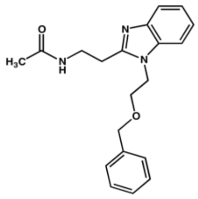	4.75	95.10	0.97	4.90	98.14	3.09	6.65
**57**	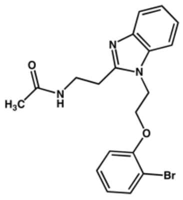	9.38	306.00	0.94	9.97	325.19	3.52	3.60

**58**	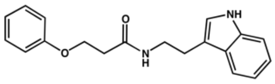	14.20	536.00	0.84	16.99	641.15	3.58	14.60
**59**	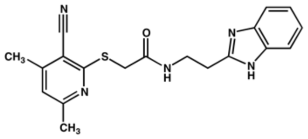	61.50	433.00	0.96	64.36	453.17	2.93	2.17
**60**	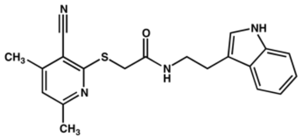	155.00	485.00	0.79	194.48	608.53	3.63	11.00
**61**	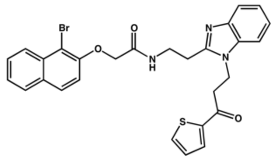	157.00	355.00	0.04	3,634.26	8,217.59	4.61	0.03
**62**	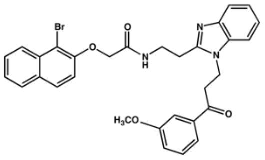	125.00	331.00	0.02	5,186.72	13,734.44	4.84	0.04
**63**	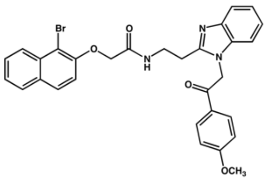	40.40	50.10	0.02	2,104.17	2,609.38	4.81	0.03

CL_{int}, intrinsic clearance after incubation with human and mouse microsomes; fu, fraction unbound; CL_{int_u}, corrected clearance (CL_{int}/fu); eLog*D*, experimentally determined distribution coefficient; PAMPA, parallel artificial membrane permeability assay.

Table S6. Structures and *in vivo* PK profiles of set 2 benzimidazole derivatives.

	IV							PO				
Cpd	$T_{1/2}$(h)	C_0 (ng/mL)	VSS (L/kg)	AUC (ng*h/mL)	CLp (L/h/kg)	fu	CLp_u (L/h/kg)	$T_{1/2}$ (h)	C_{max} (ng/mL)	T_{max} (h)	AUC (ng*h/mL)	F(%)
**BZ**	0.8	961	1.1	1020	1.0	0.99	1.0	1.5	404	0,4	1040	90
**28**	0.52	1930	0.43	1420	0.73	0.18	3.98	0.82	275	0.51	499	35
**1**	0.21	988	1.02	250	4.01	0.23	17.39	0.73	21	0.32	22.51	9
**2**	0.63	1040	1.03	506	2.03	0.49	4.16	1.21	54	0.83	125.11	25
**10**	0.14	660	1.21	156	6.52	0.34	18.84	-	0	-	0	0
**3**	0.35	12000	0.45	916	2.31	0.16	14.55	-	9	0.44	9.91	1
**11**	0.13	400	1.92	77	12.91	0.03	480.66	-	0	-	-	-
**8**	0.21	925	1.04	322	3.31	0.14	23.77	0.42	61	0.31	44.62	14

IV, intravenous administration; PO, oral administration; $T_{1/2}$, plasma half-life; C_0, concentration at time = 0; VSS, steady-state volume of distribution; AUC, area under the curve; CLp, plasma clearance; *fu*, fraction unbound; CLp_u, plasma clearance corrected for the fraction unbound; C_{max}, peak plasma concentration; T_{max}, time of peak plasma concentration; F, bioavailability.

NMR spectra

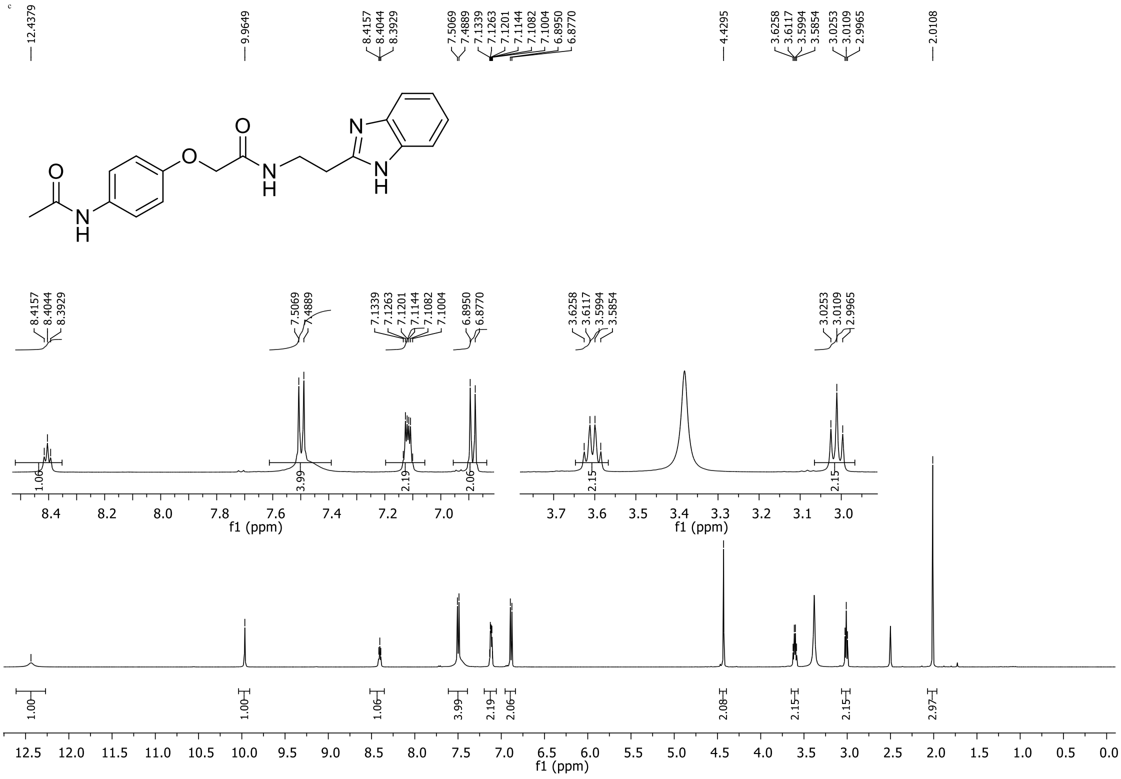

^{1}H NMR of **2** (500 MHz, DMSO).

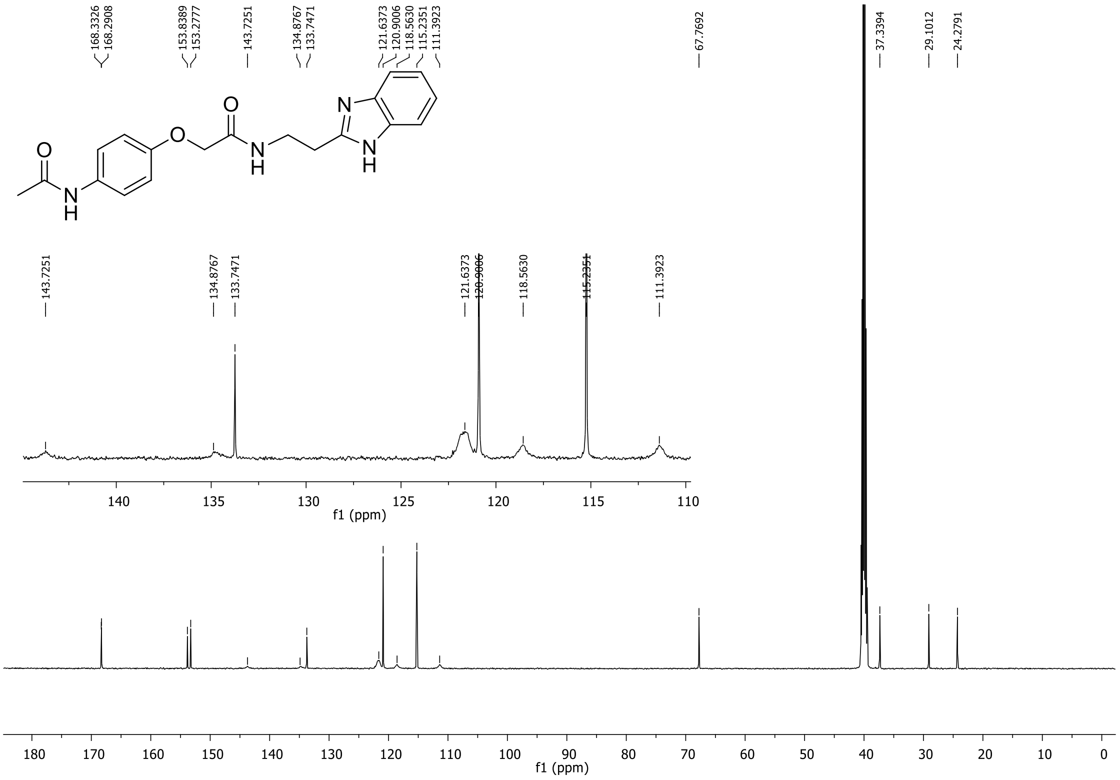

^{1}H NMR of **2** (125 MHz, DMSO).

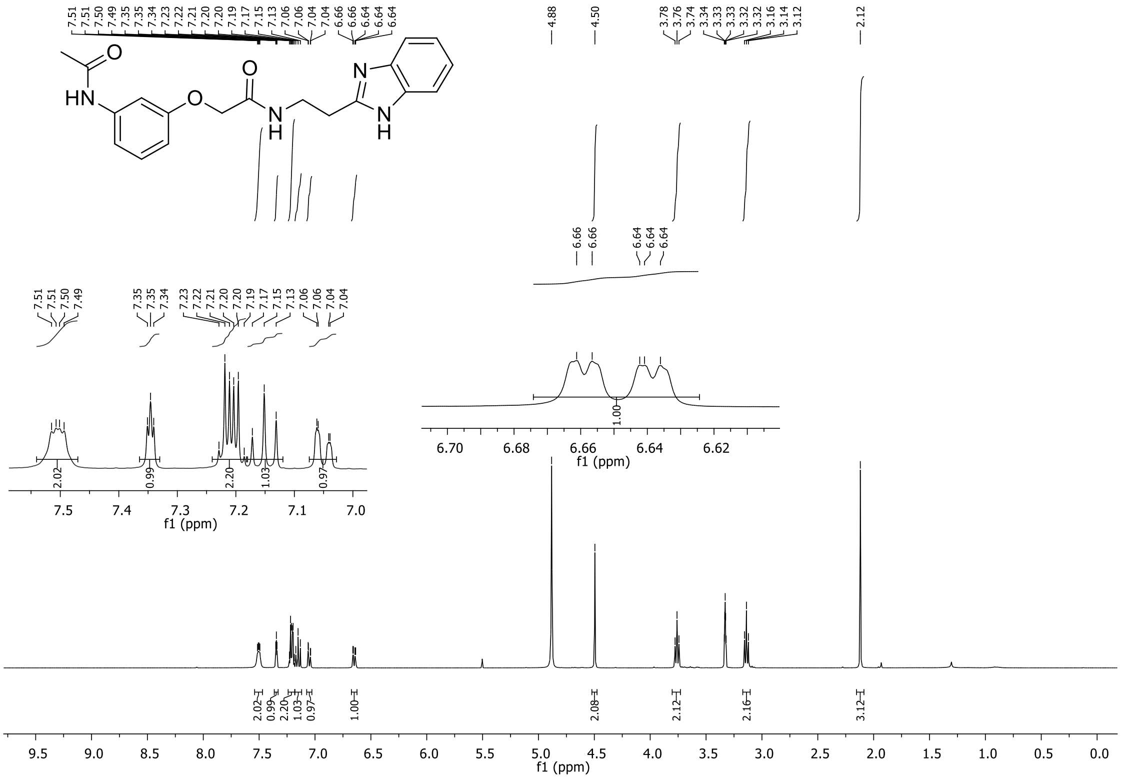

^{1}H NMR of **3** (400 MHz, MeOD).

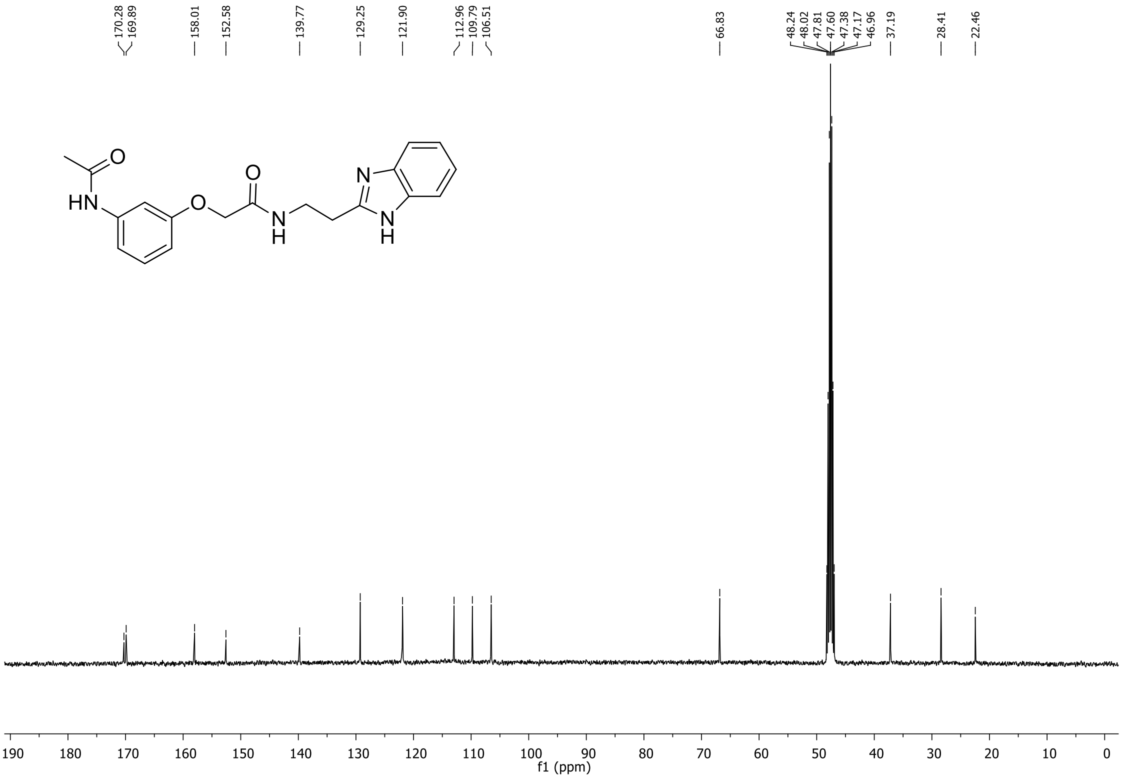

^{13}C NMR of **3** (101 MHz, MeOD).

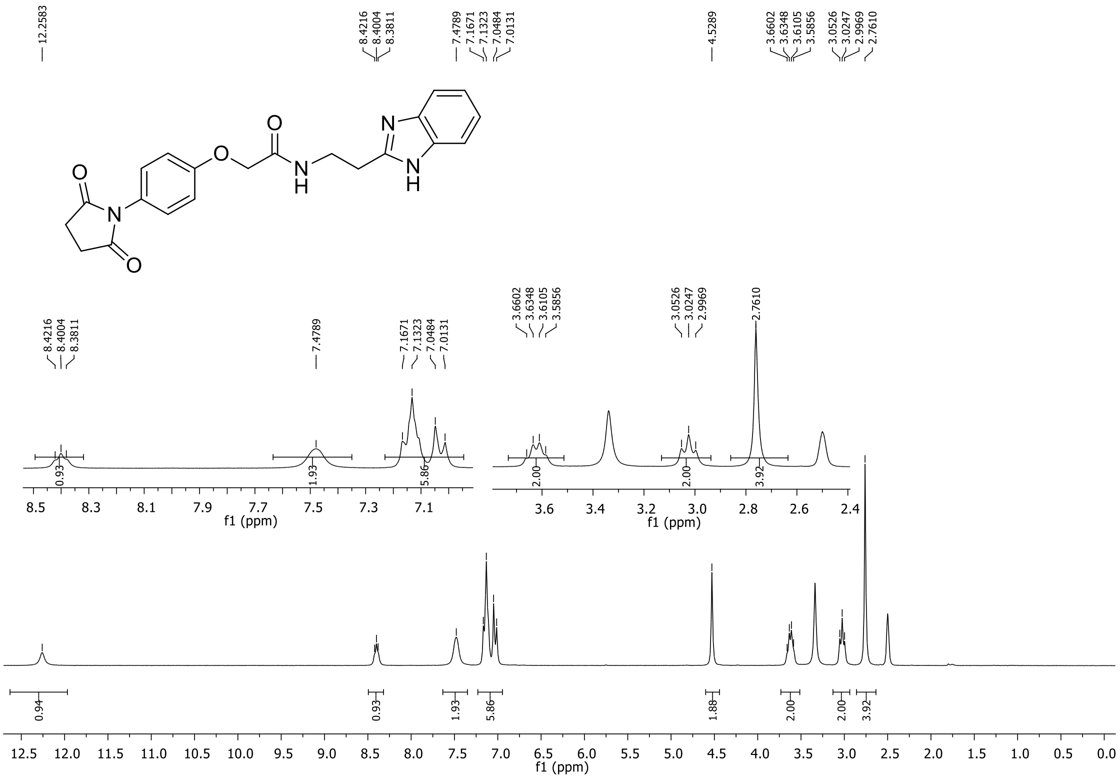

^{1}H NMR of **4** (250 MHz, DMSO).

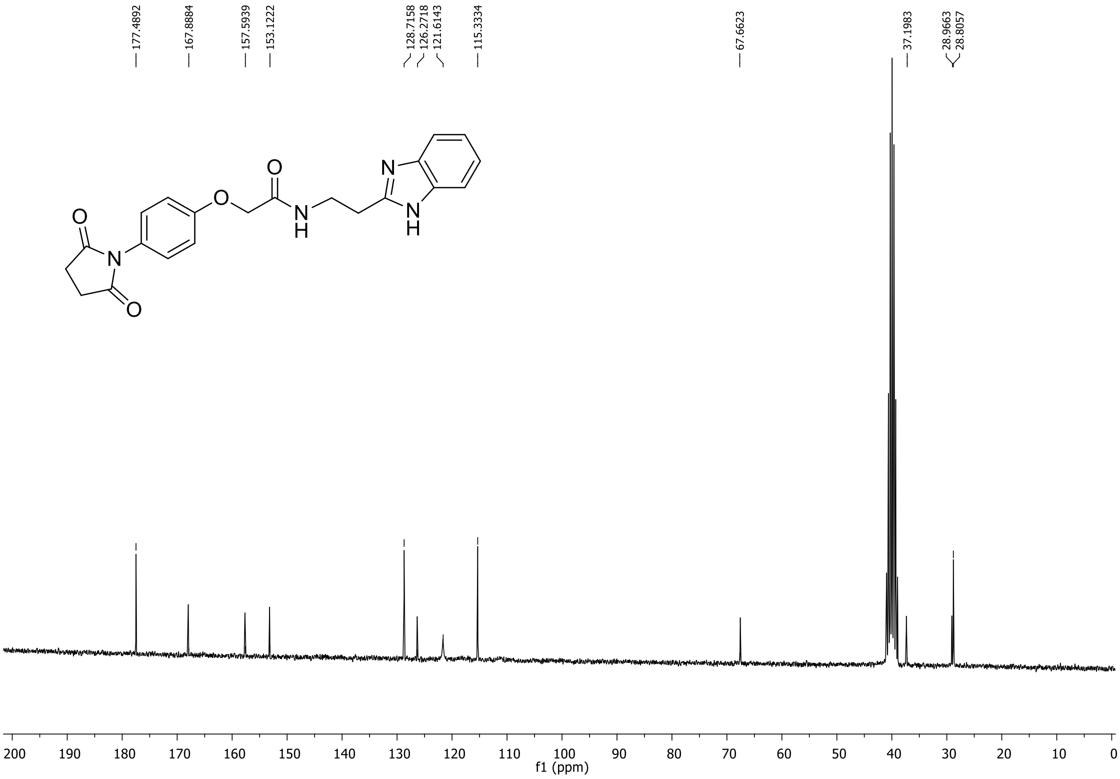

^{1}H NMR of **4** (63 MHz, DMSO).

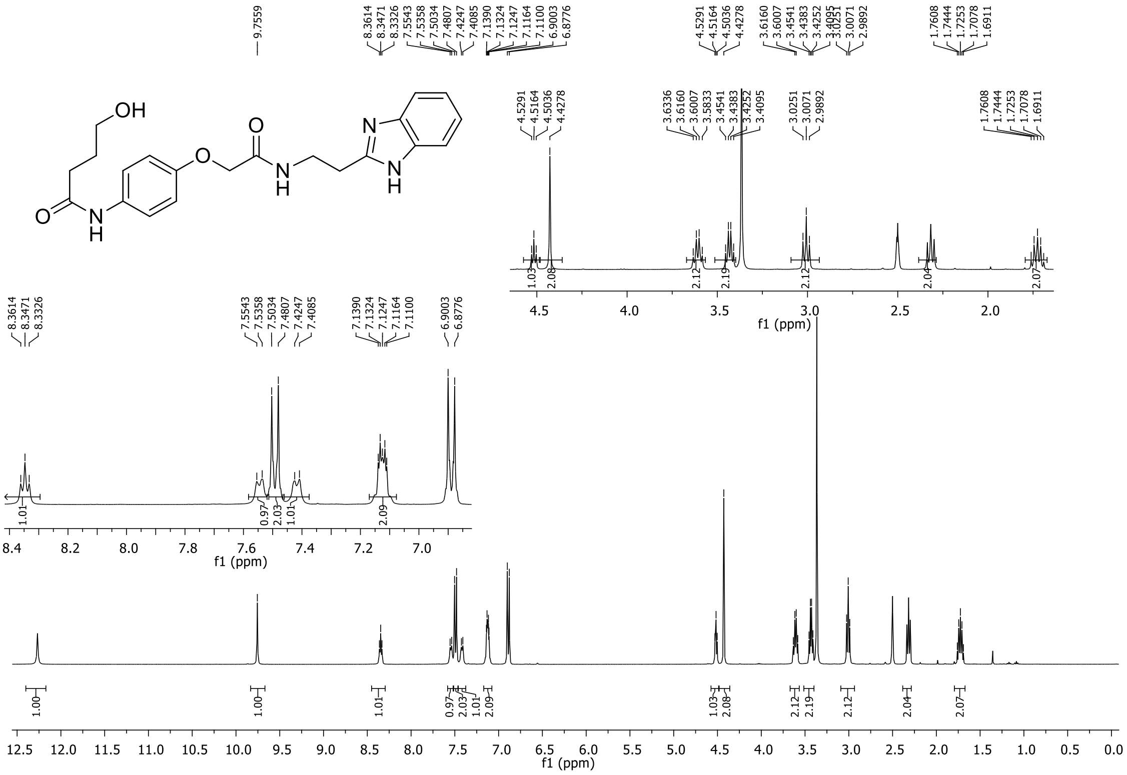

^{1}H NMR of **5** (400 MHz, DMSO).

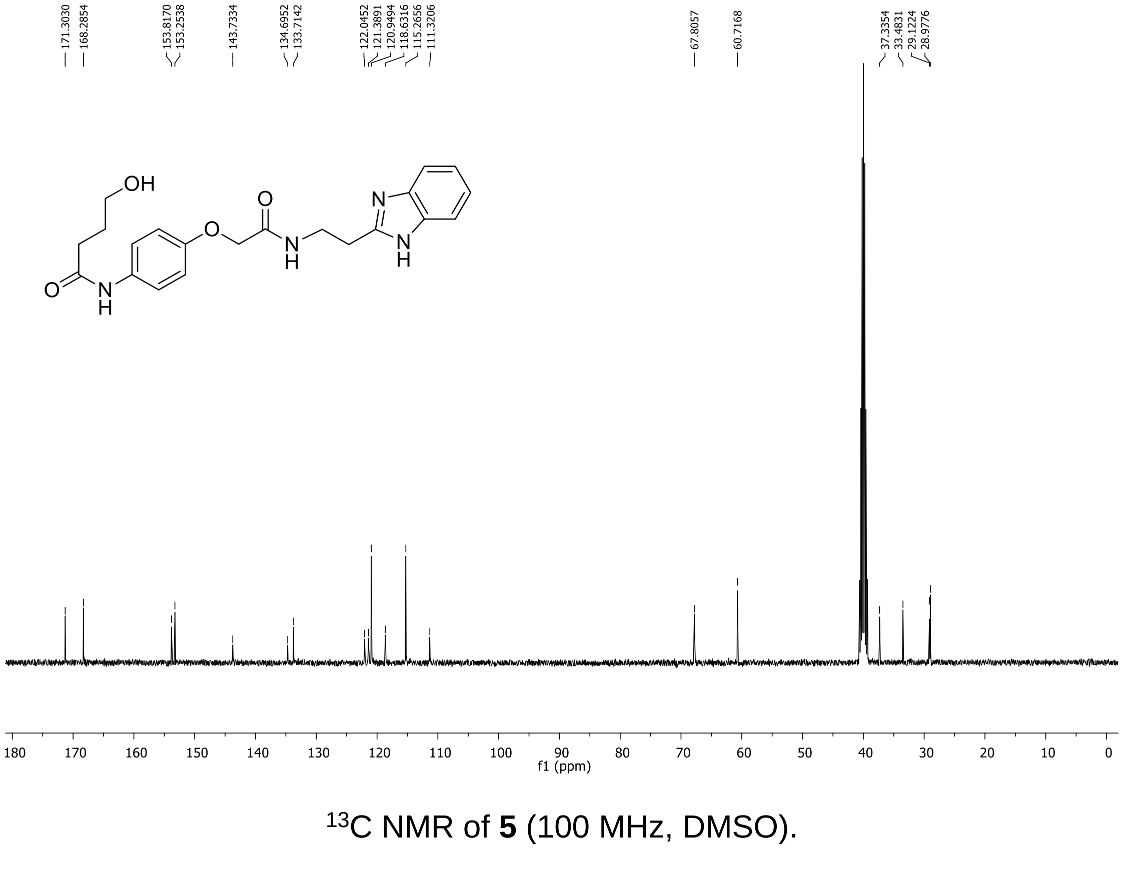

^{13}C NMR of **5** (100 MHz, DMSO).

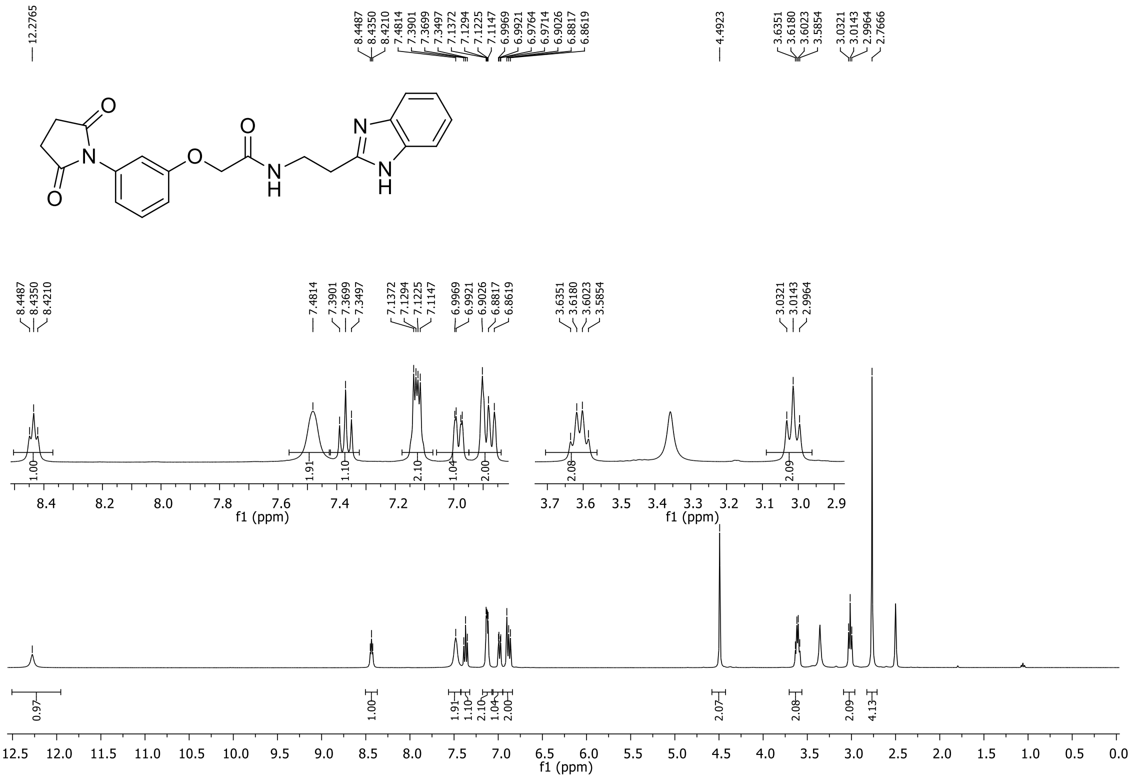

^{1}H NMR of **6** (400 MHz, DMSO).

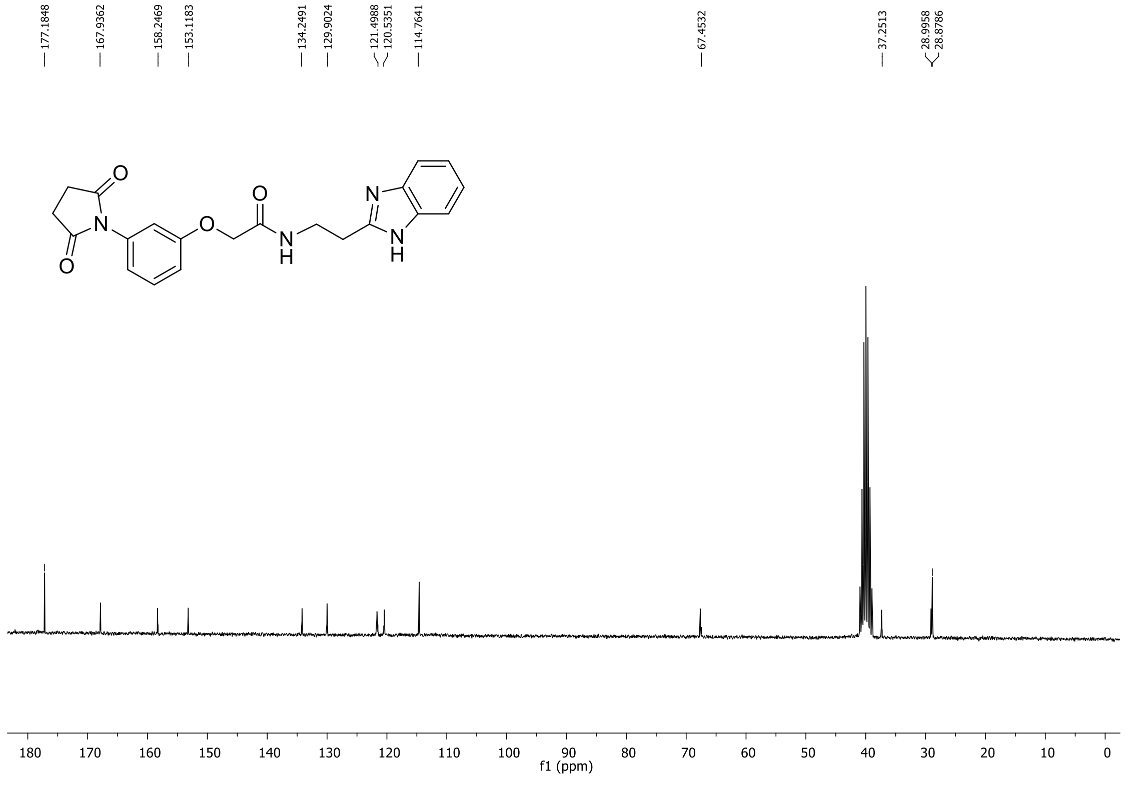

^{1}H NMR of **6** (63 MHz, DMSO).

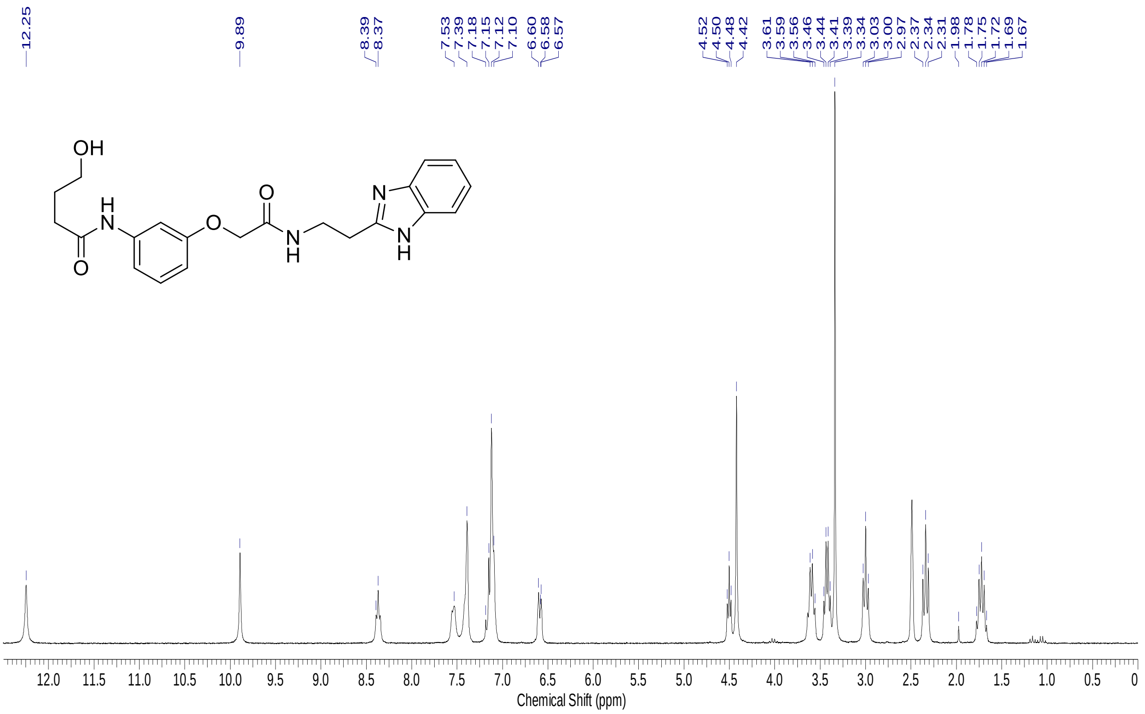

^{1}H NMR of **7** (250 MHz, DMSO).

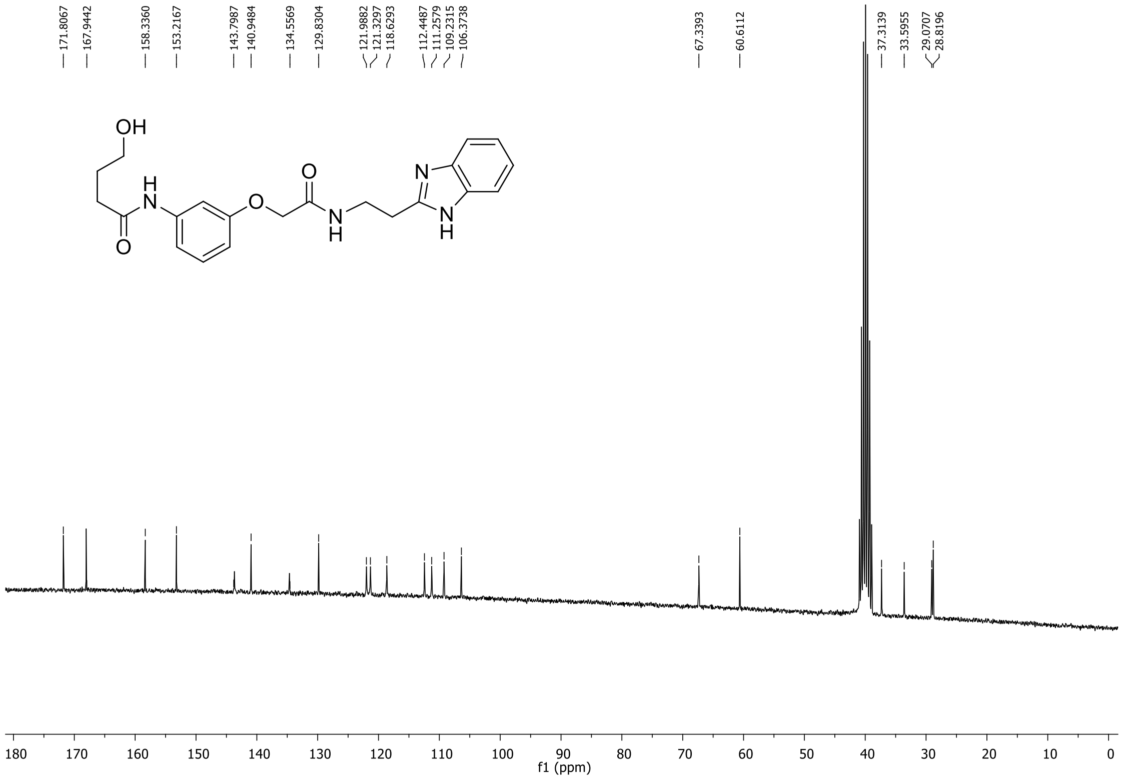

^{1}H NMR of **7** (63 MHz, DMSO).

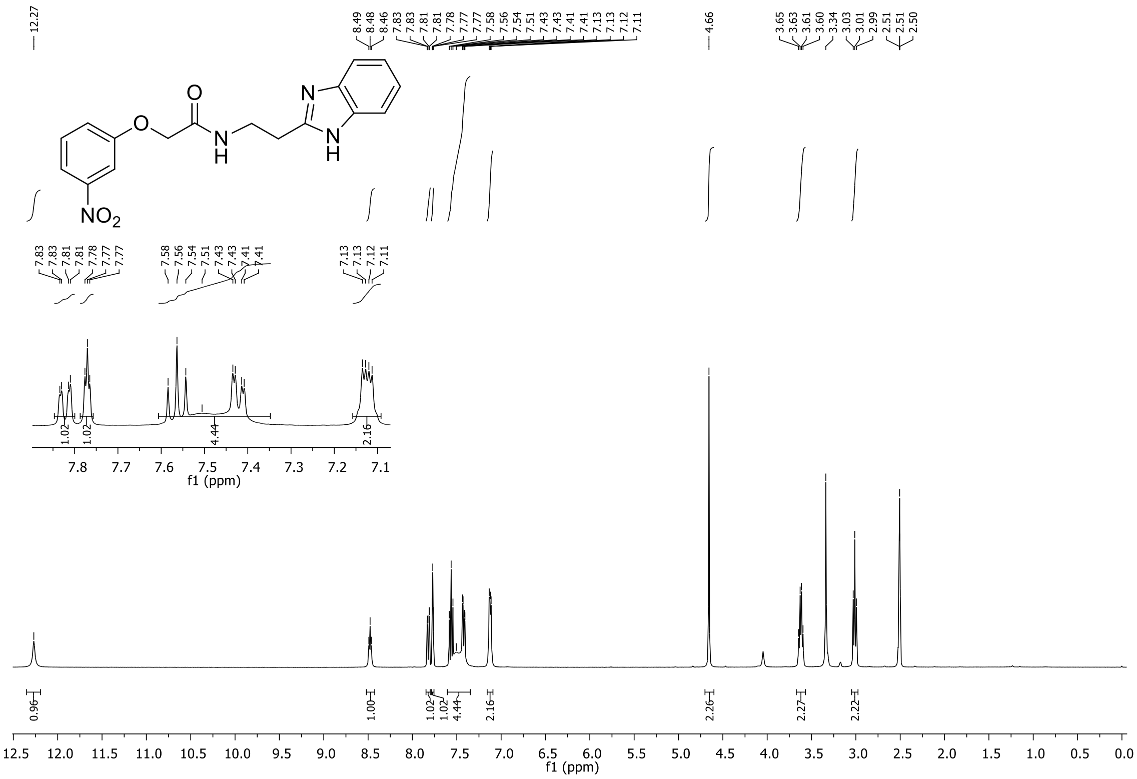

^{1}H NMR of **8** (400 MHz, DMSO).

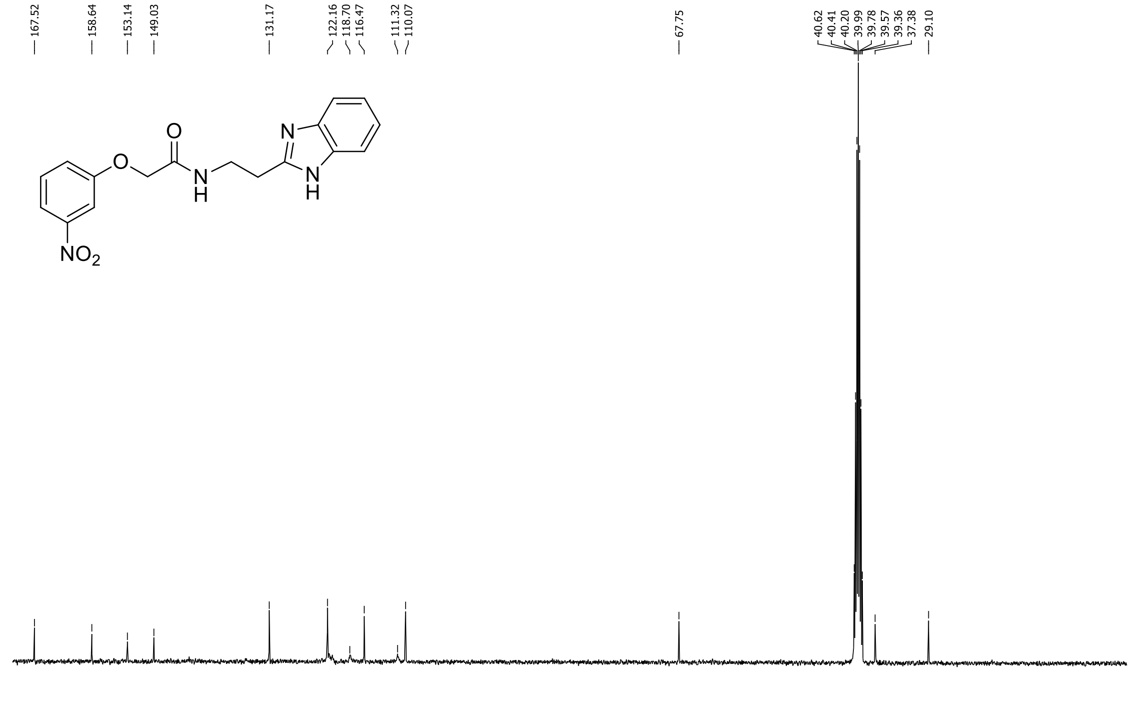

^{13}C NMR of **8** (101 MHz, DMSO).

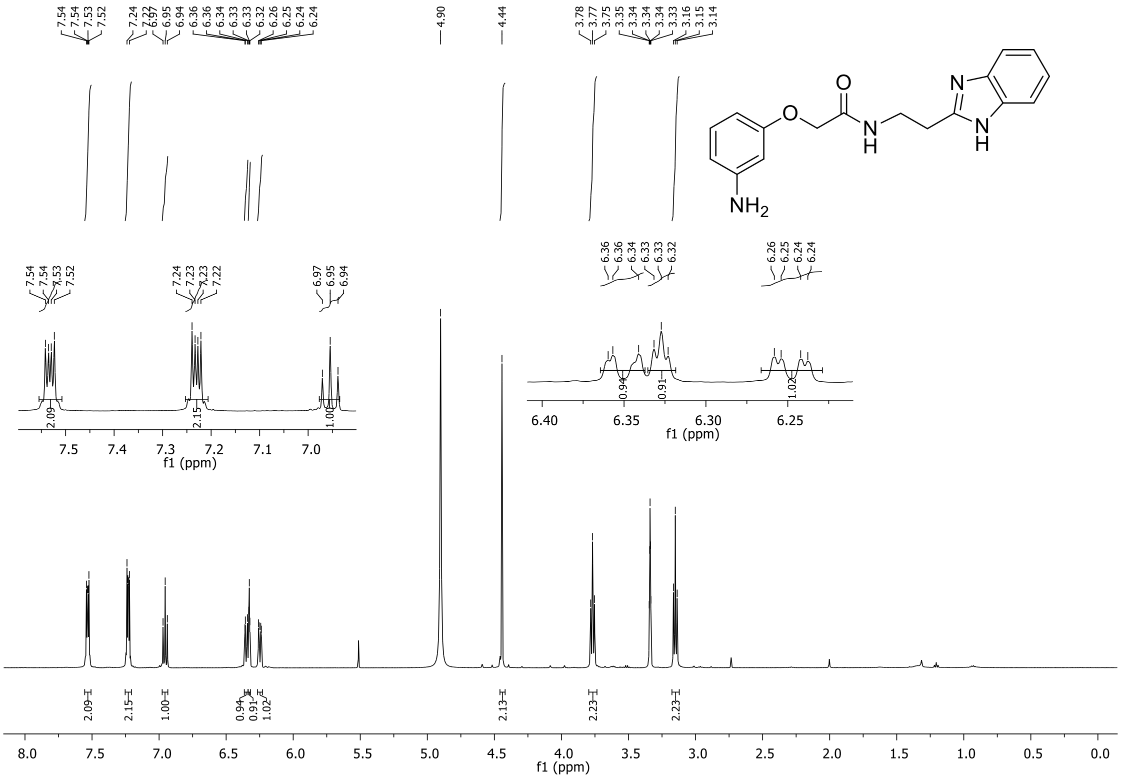

^{1}H NMR of **9** (500 MHz, MeOD).

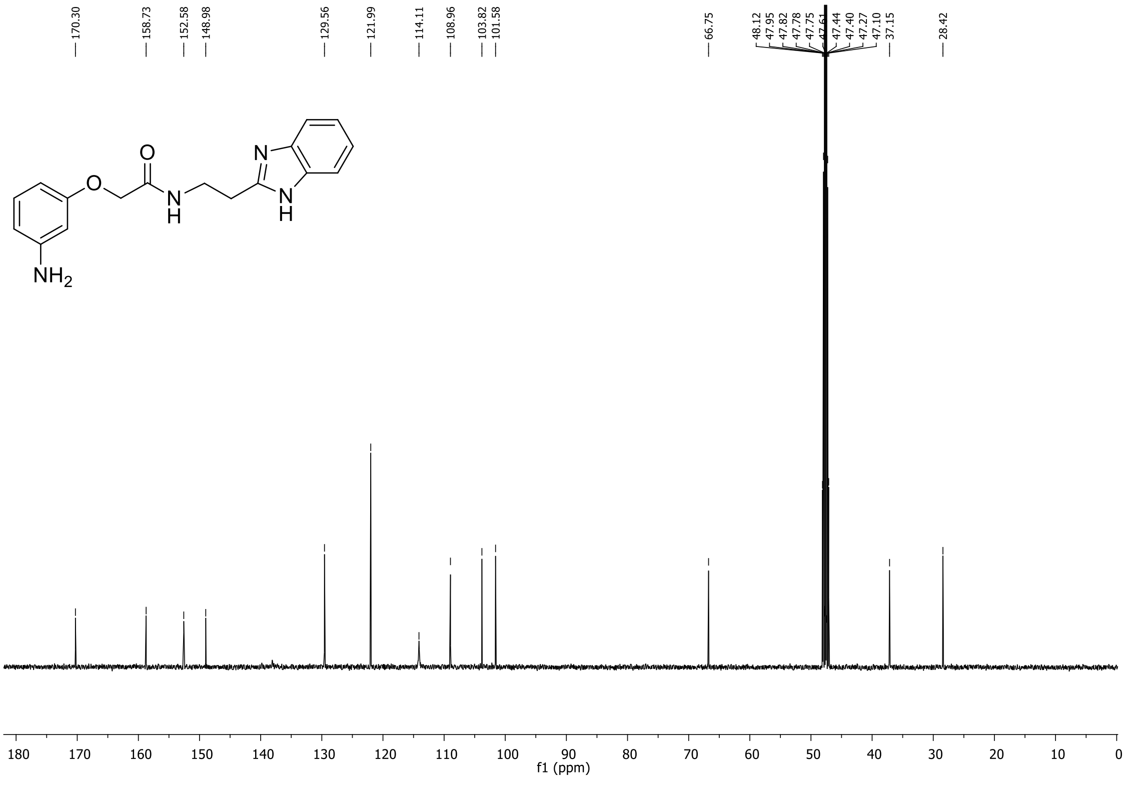

^{13}C NMR of **9** (126 MHz, MeOD).

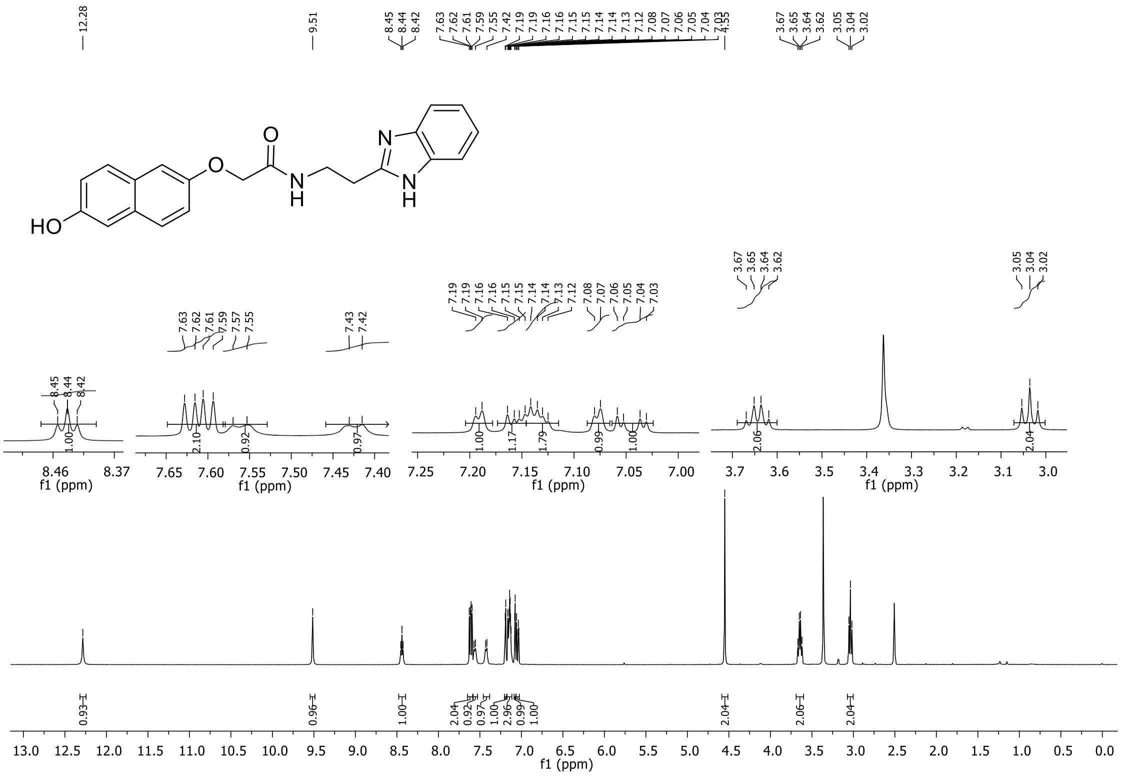

^{1}H NMR of **10** (400 MHz, DMSO).

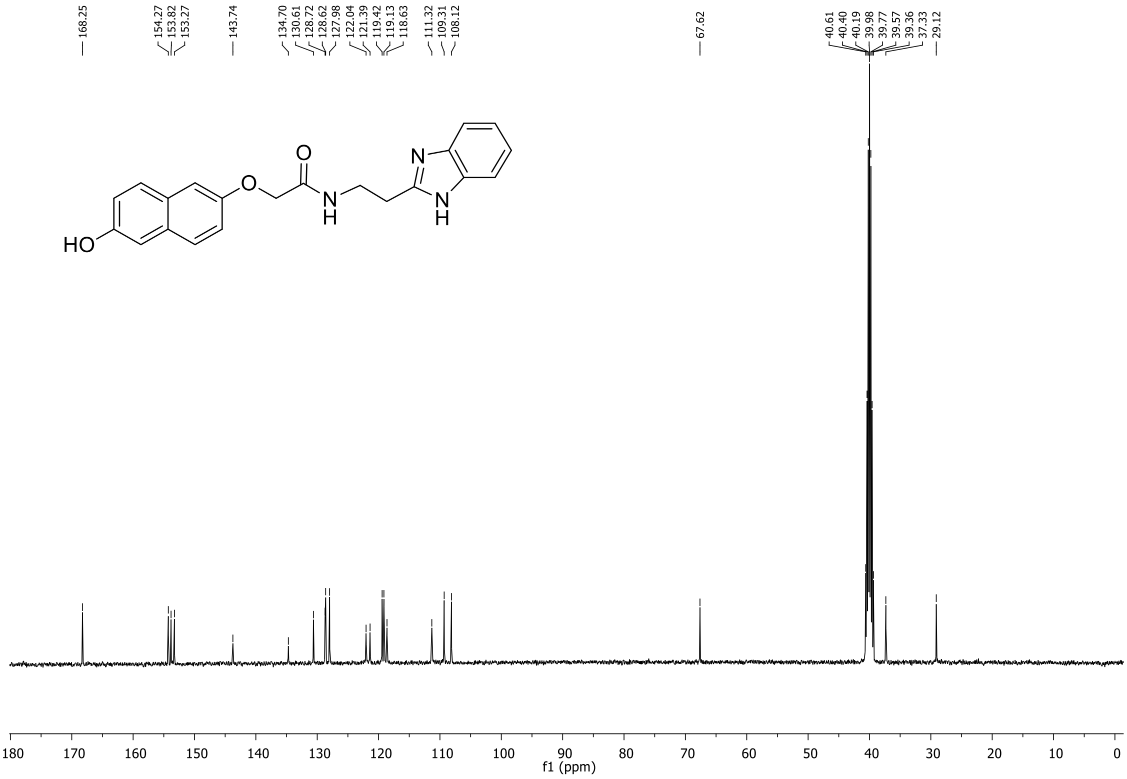

^{13}C NMR of **10** (101 MHz, DMSO).

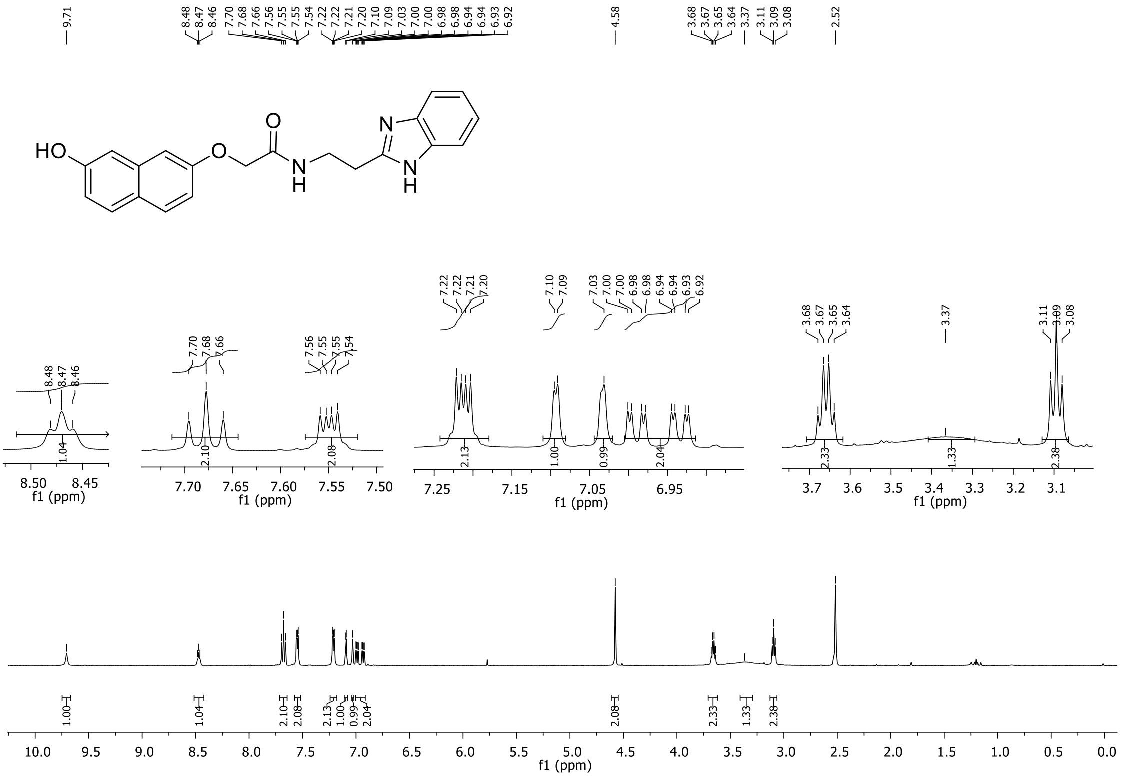

^{1}H NMR of **11** (500 MHz, DMSO).

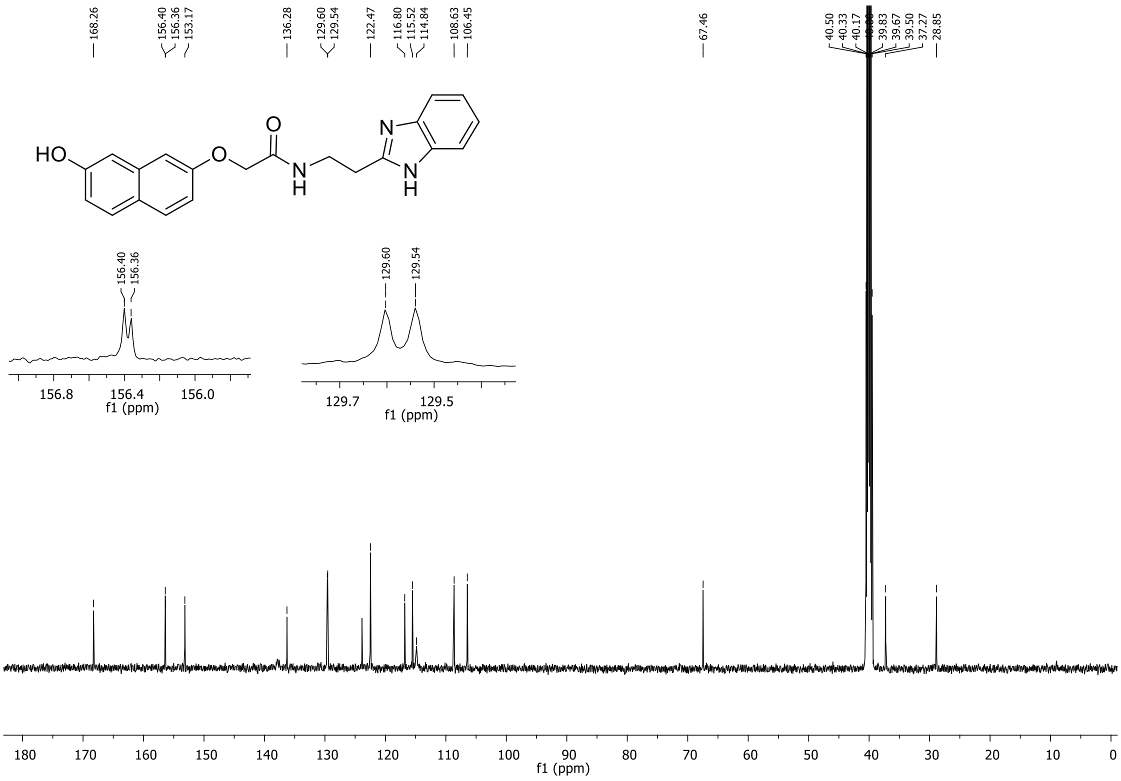

^{13}C NMR of **11** (126 MHz, DMSO).

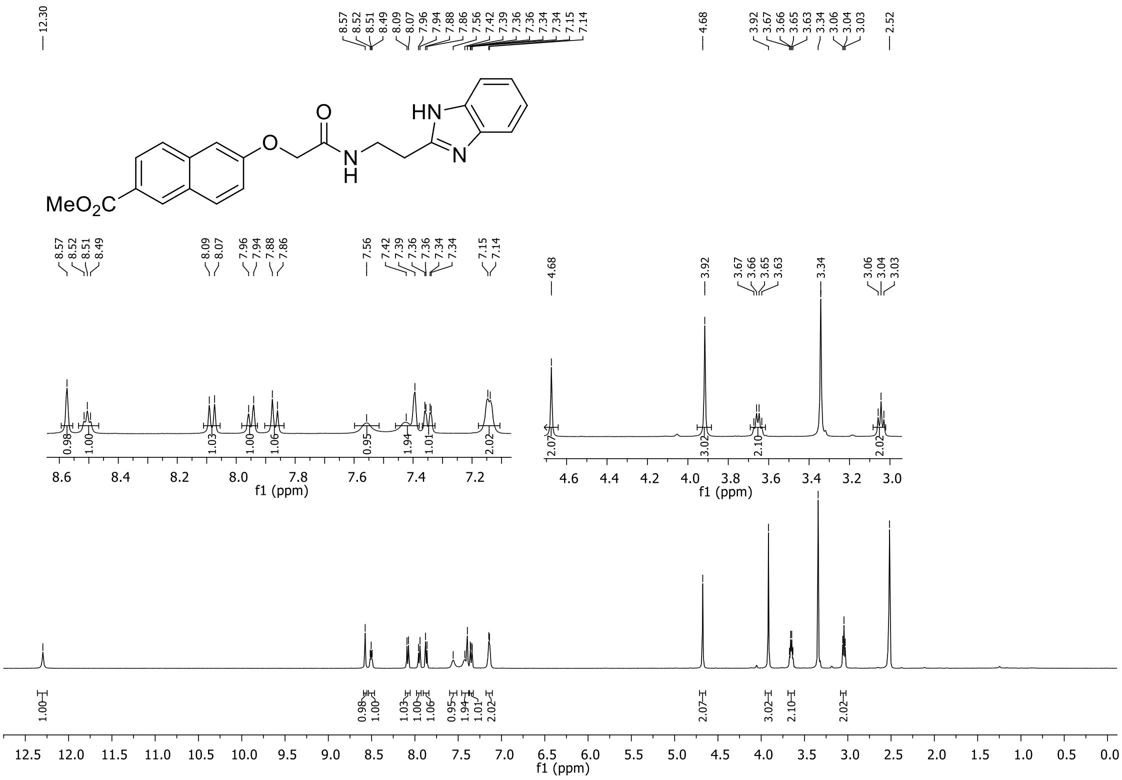

^{1}H NMR of **12** (500 MHz, DMSO).

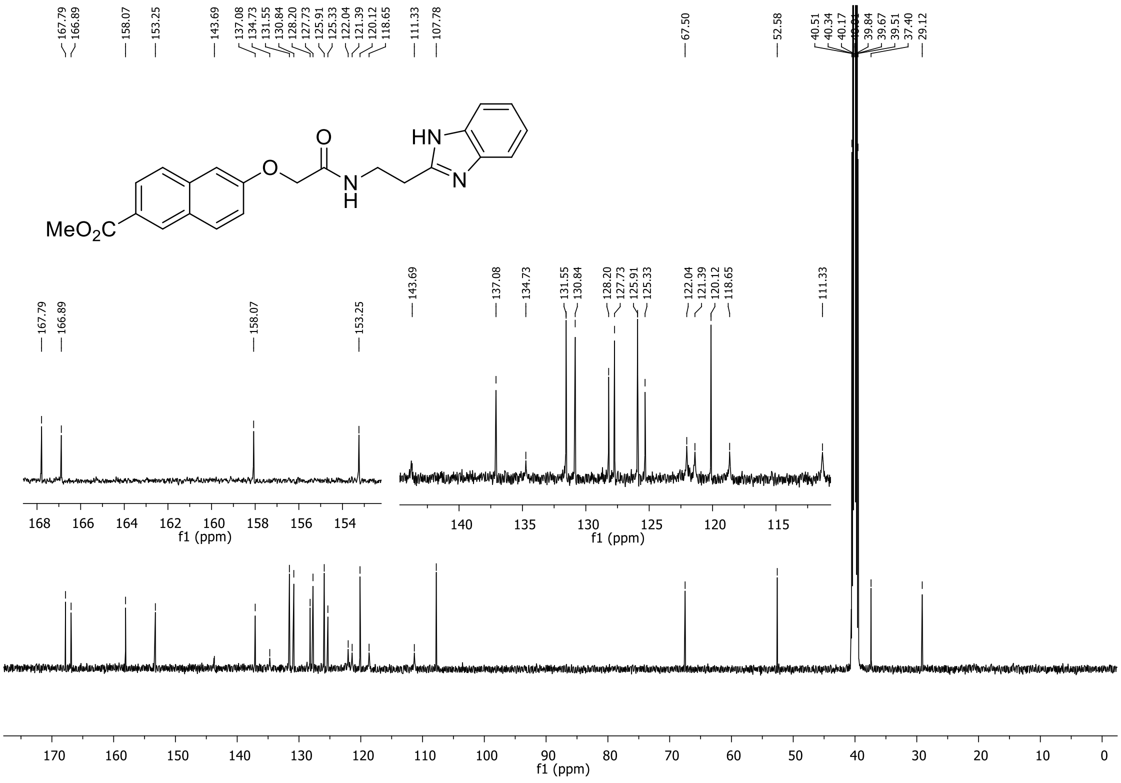

^{13}C NMR of **12** (126 MHz, DMSO).

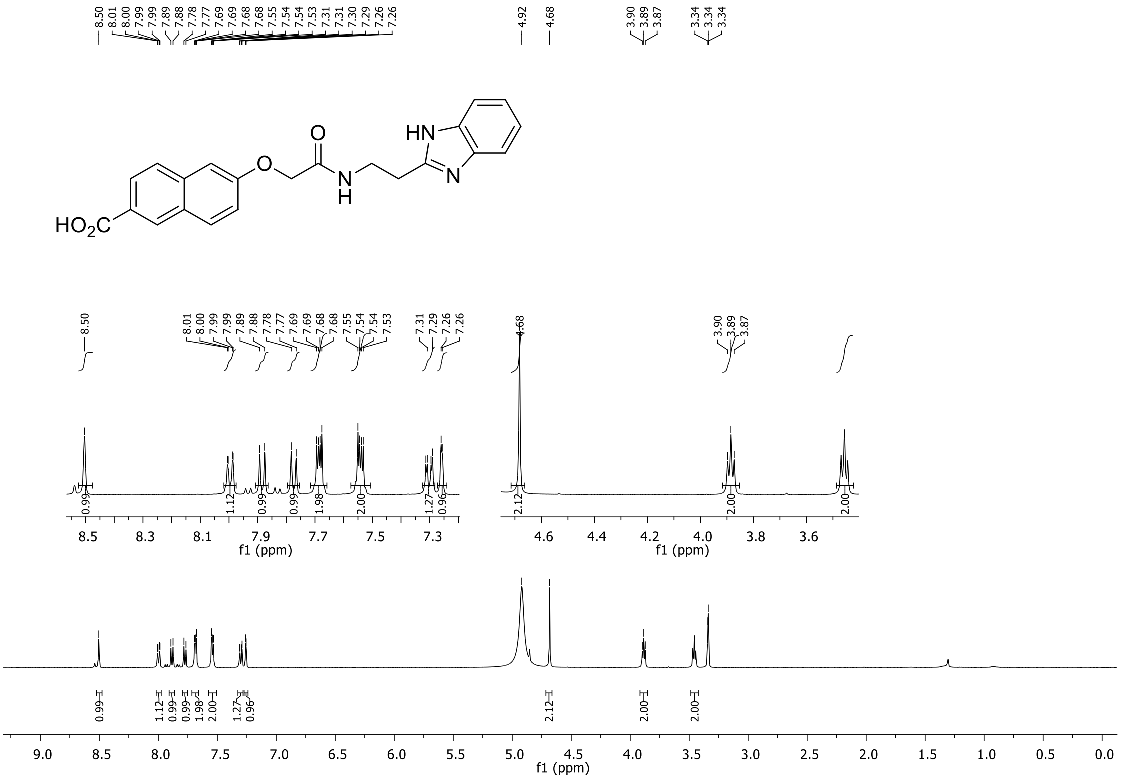

^{1}H NMR of **13** (500 MHz, MeOD).

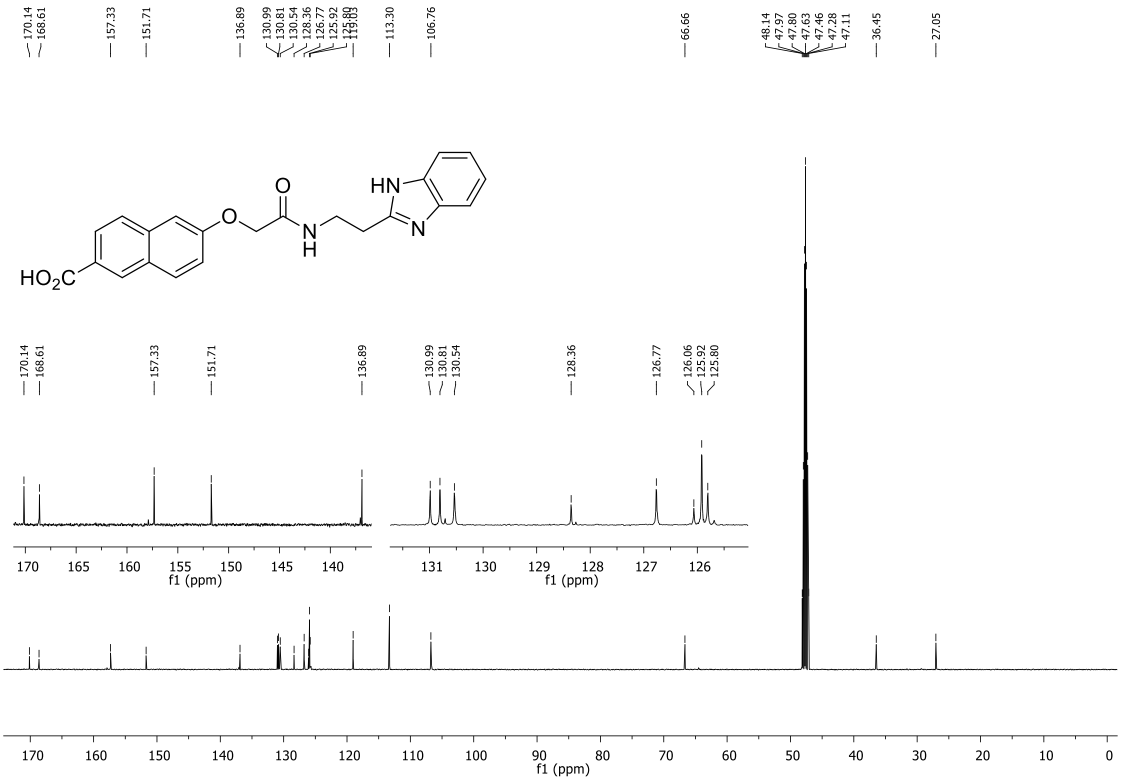

^{13}C NMR of **13** (126 MHz, MeOD).

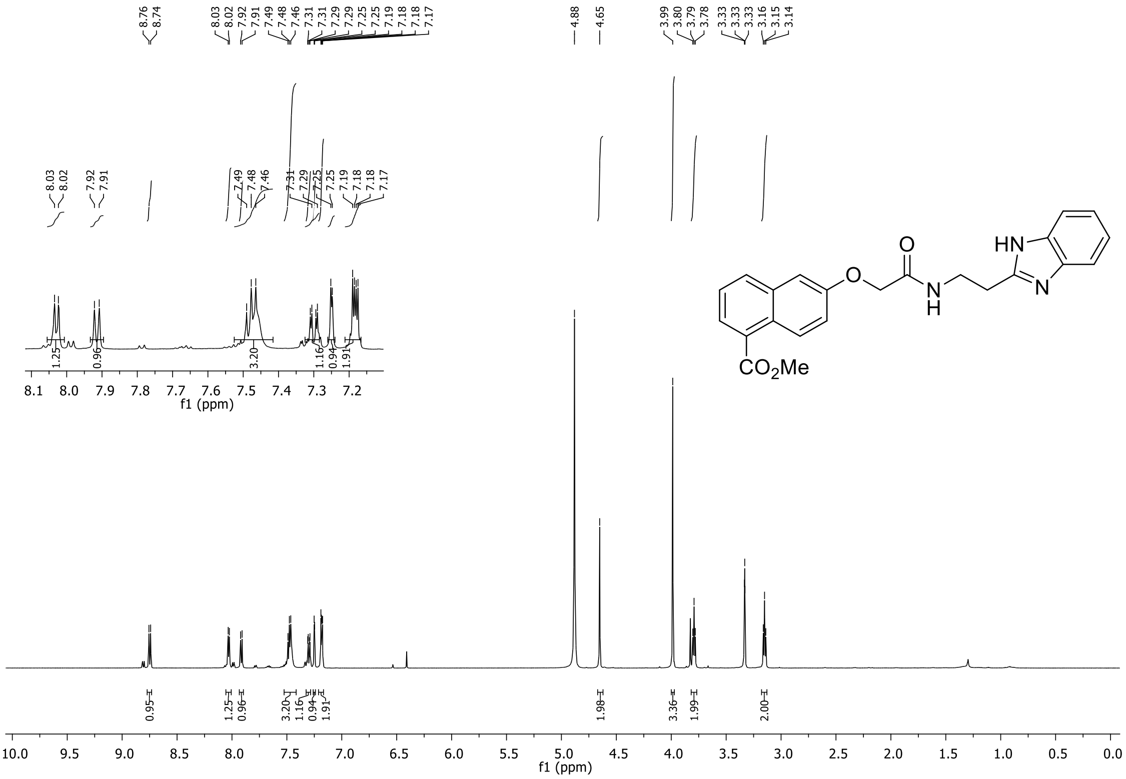

^{1}H NMR of **14** (600 MHz, MeOD).

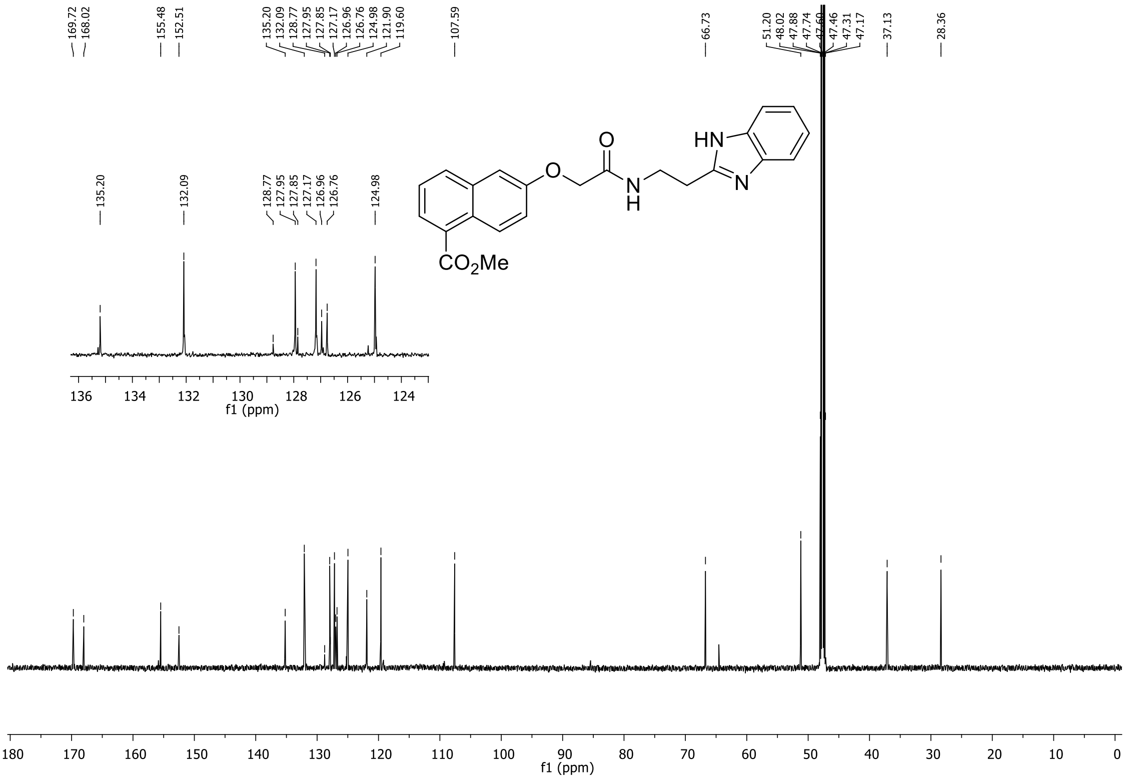

^{13}C NMR of **14** (151 MHz, MeOD).

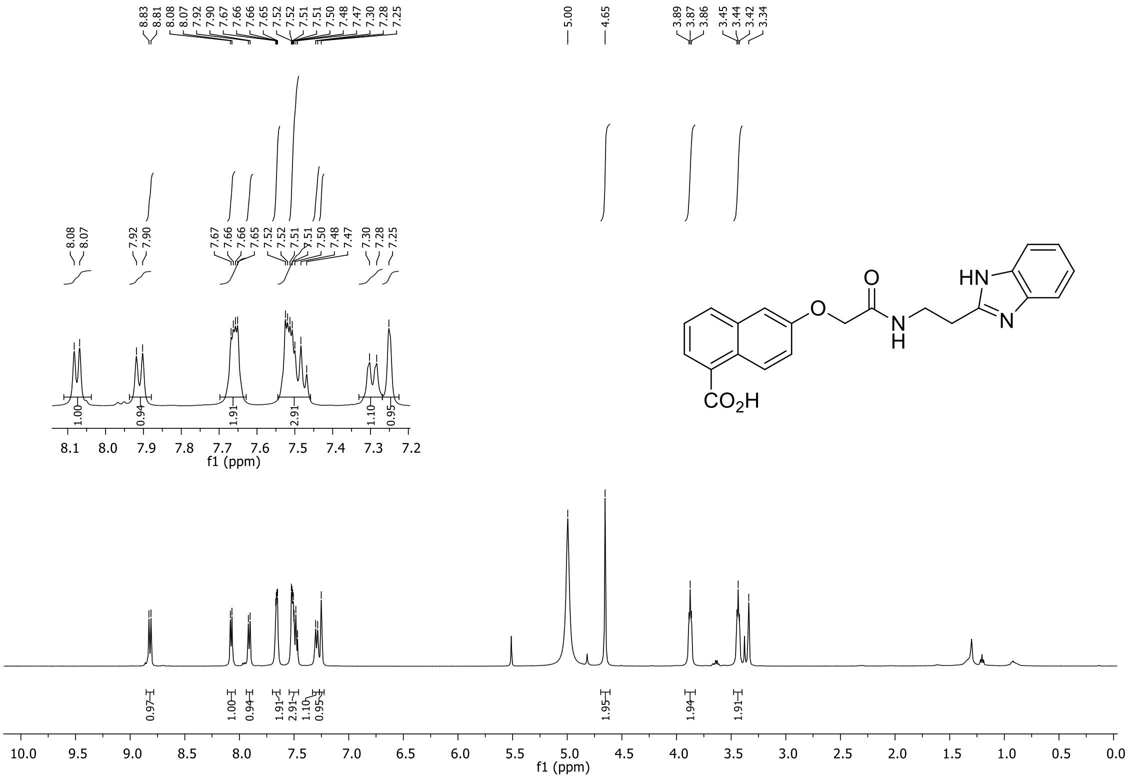

^1H NMR of **15** (500 MHz, MeOD).

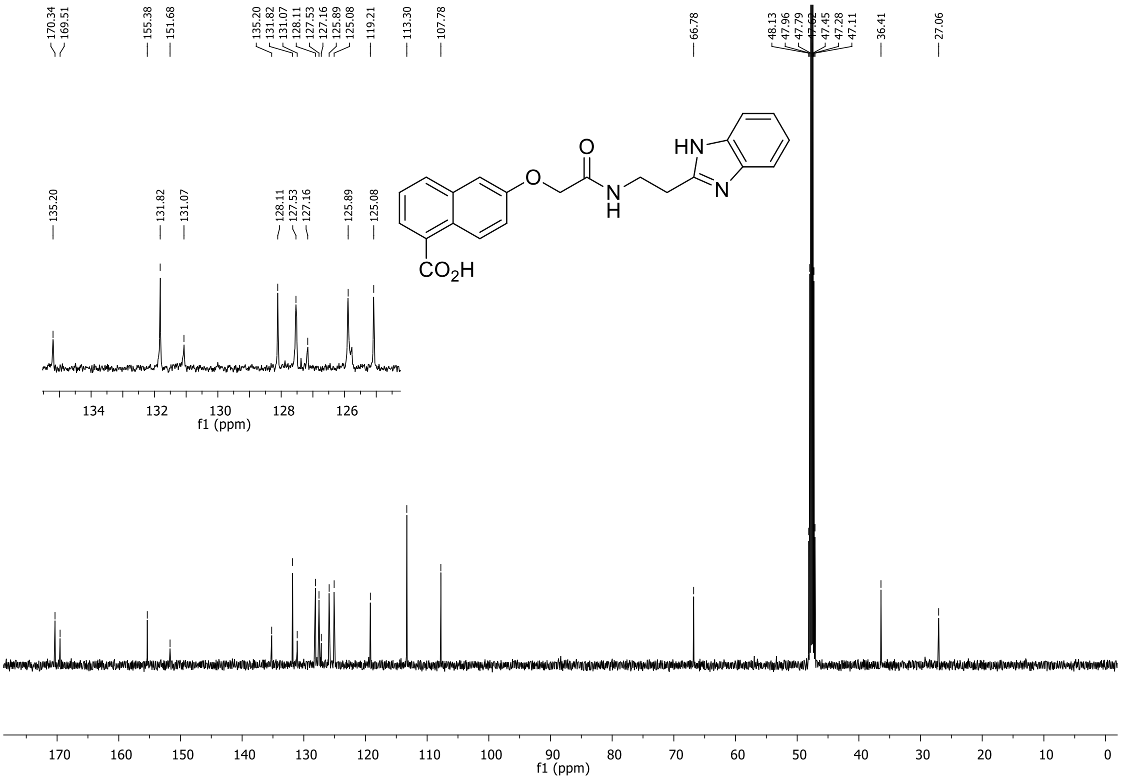

^{13}C NMR of **15** (126 MHz, MeOD).

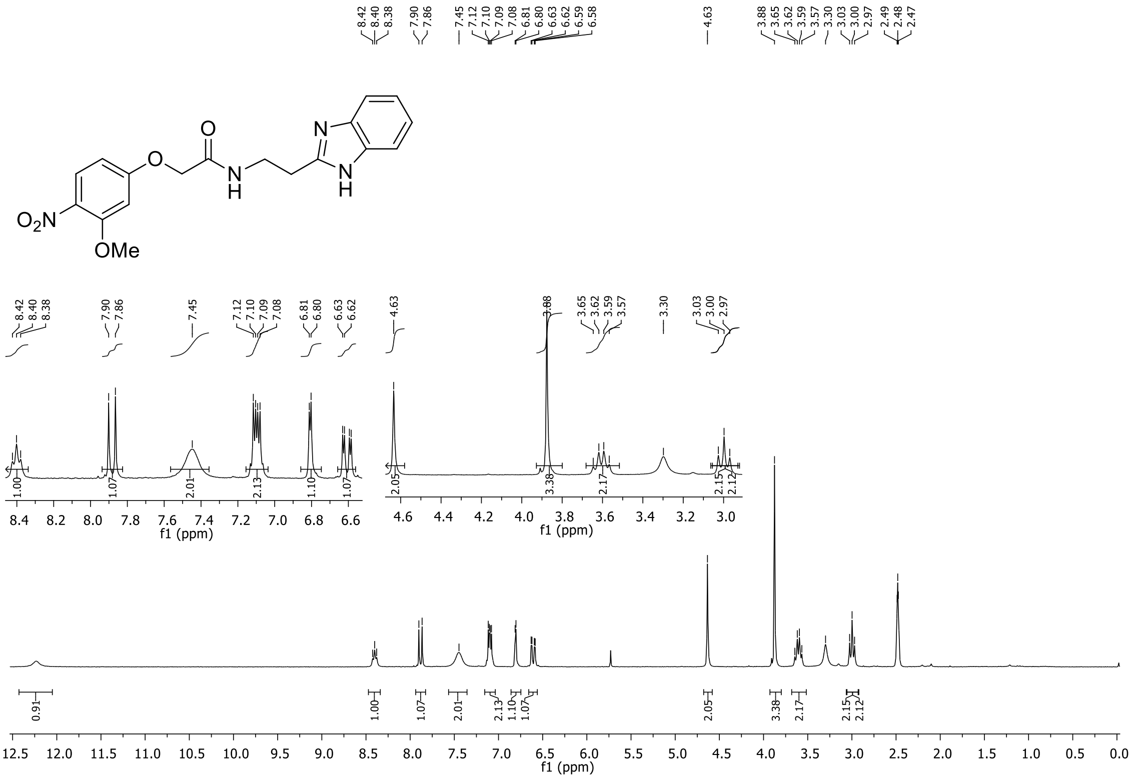

^{1}H NMR of **16** (500 MHz, DMSO).

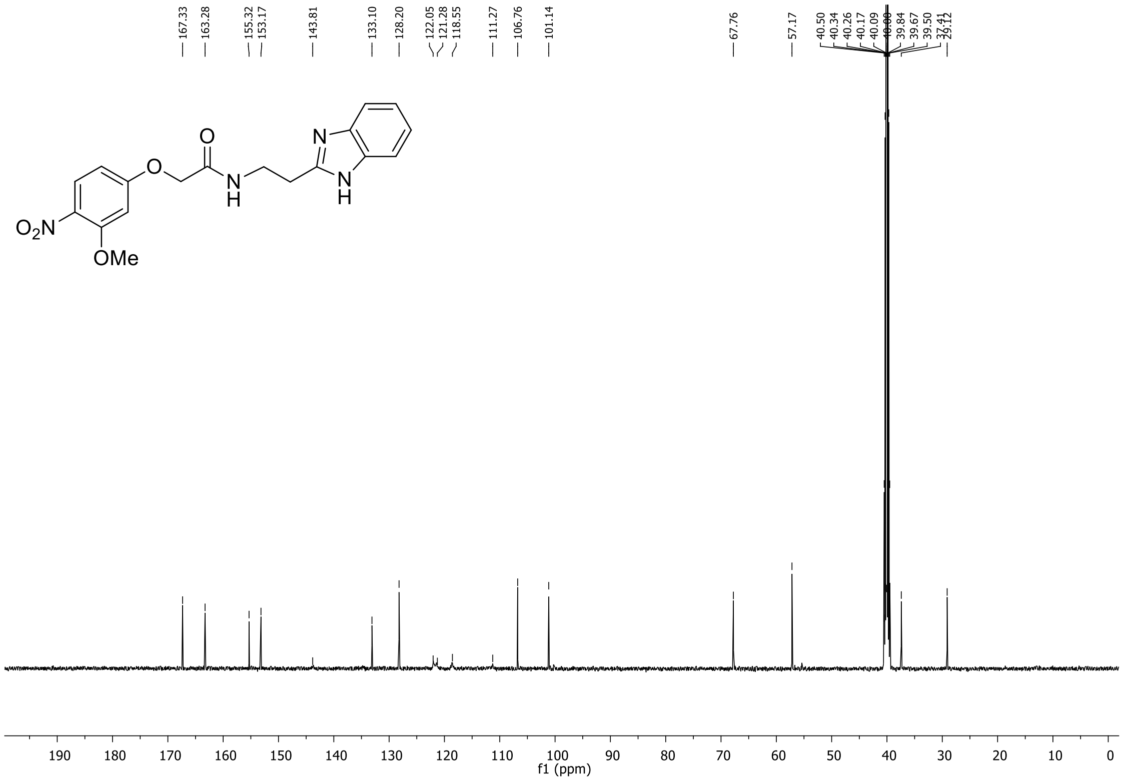

^{13}C NMR of **16** (126 MHz, DMSO).

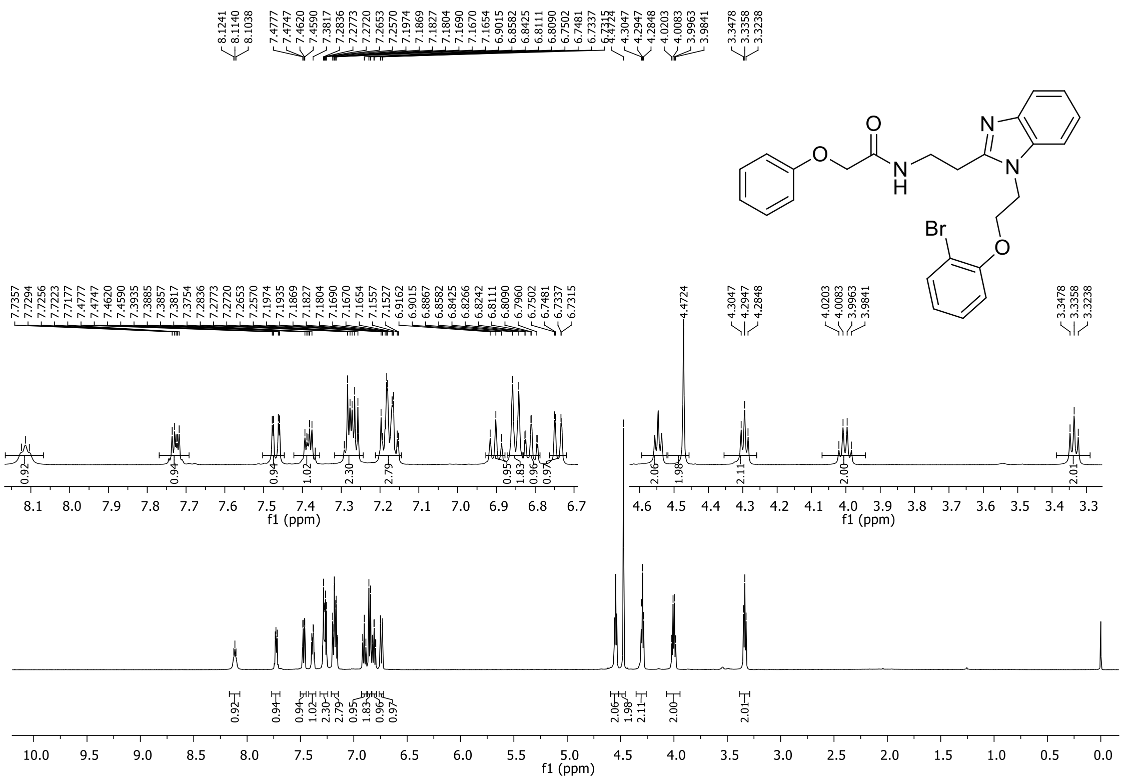

^{1}H NMR of **19** (500 MHz, $CDCl_3$).

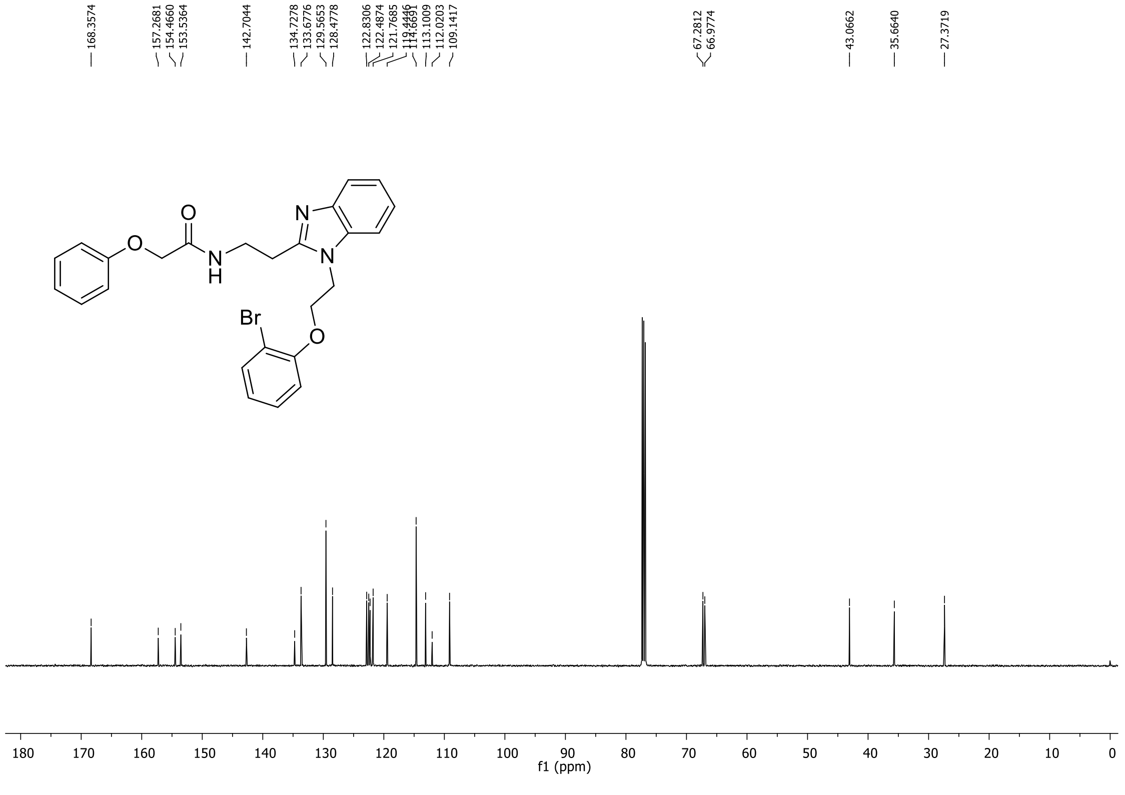

^{13}C NMR of **19** (126 MHz, $CDCl_3$).

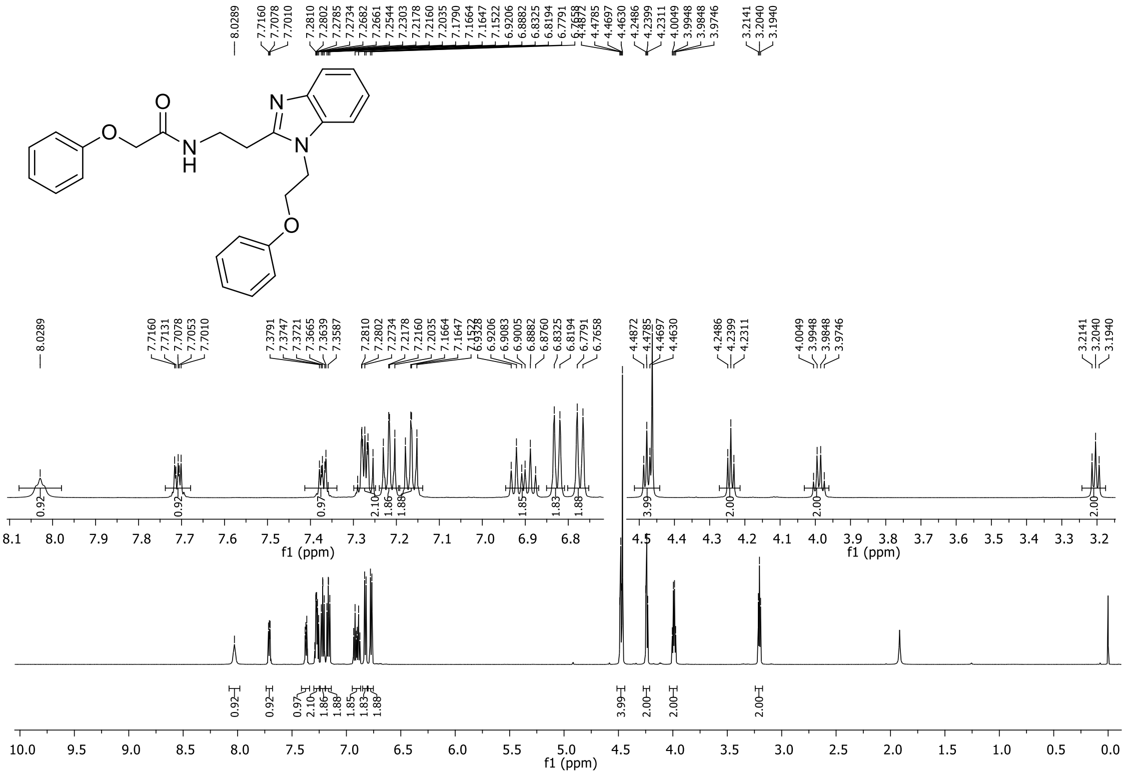

^{1}H NMR of **20** (600 MHz, $CDCl_3$).

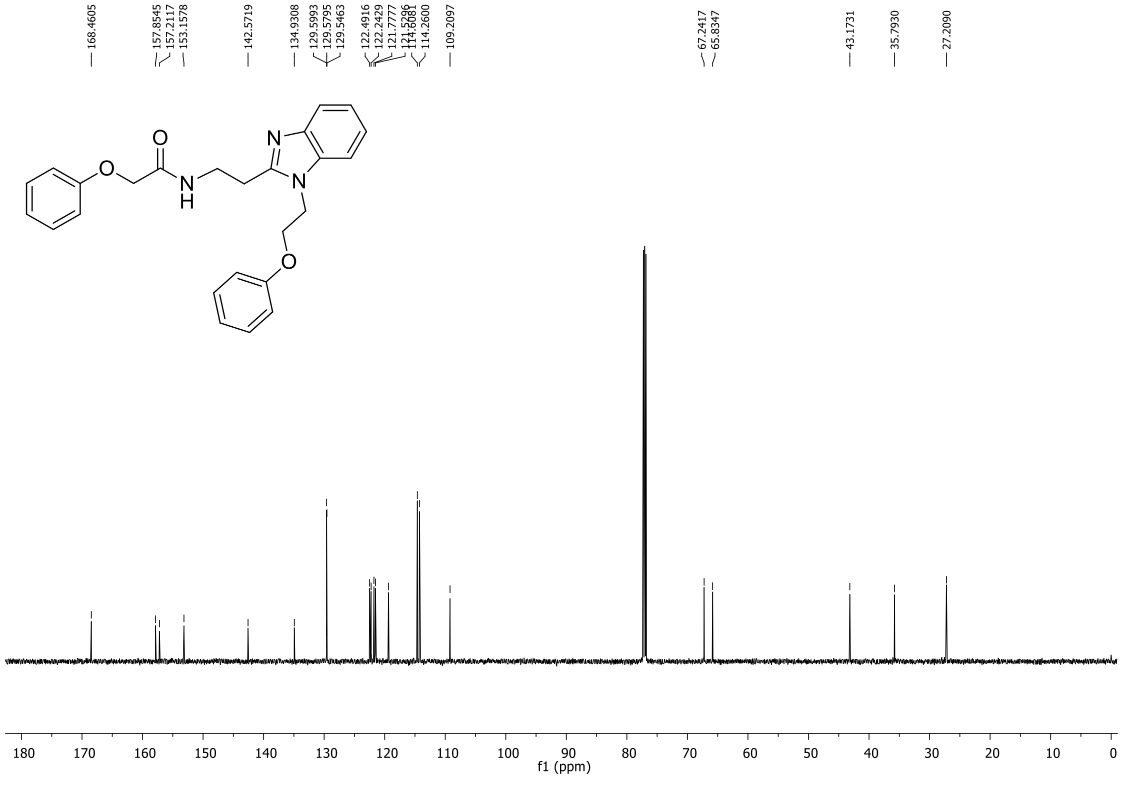

^{13}C NMR of **20** (151 MHz, $CDCl_3$).

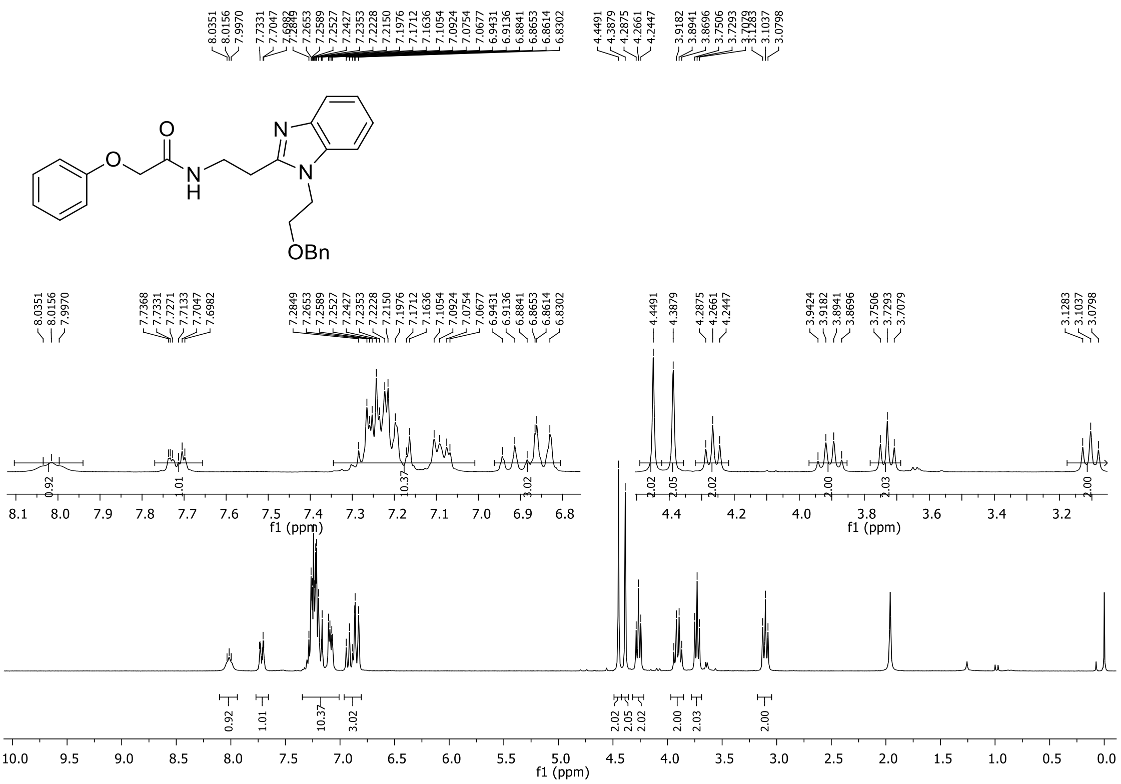

^{1}H NMR of **21** (250 MHz, $CDCl_3$).

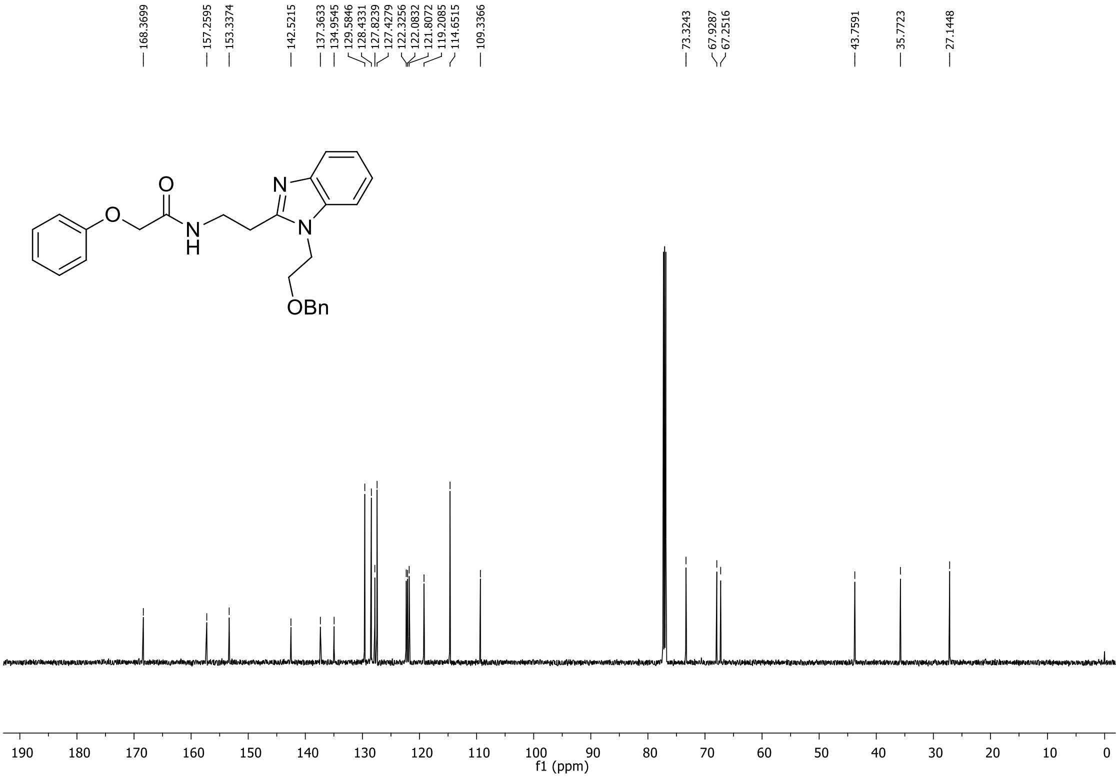

^{13}C NMR **21** (151 MHz, $CDCl_3$).

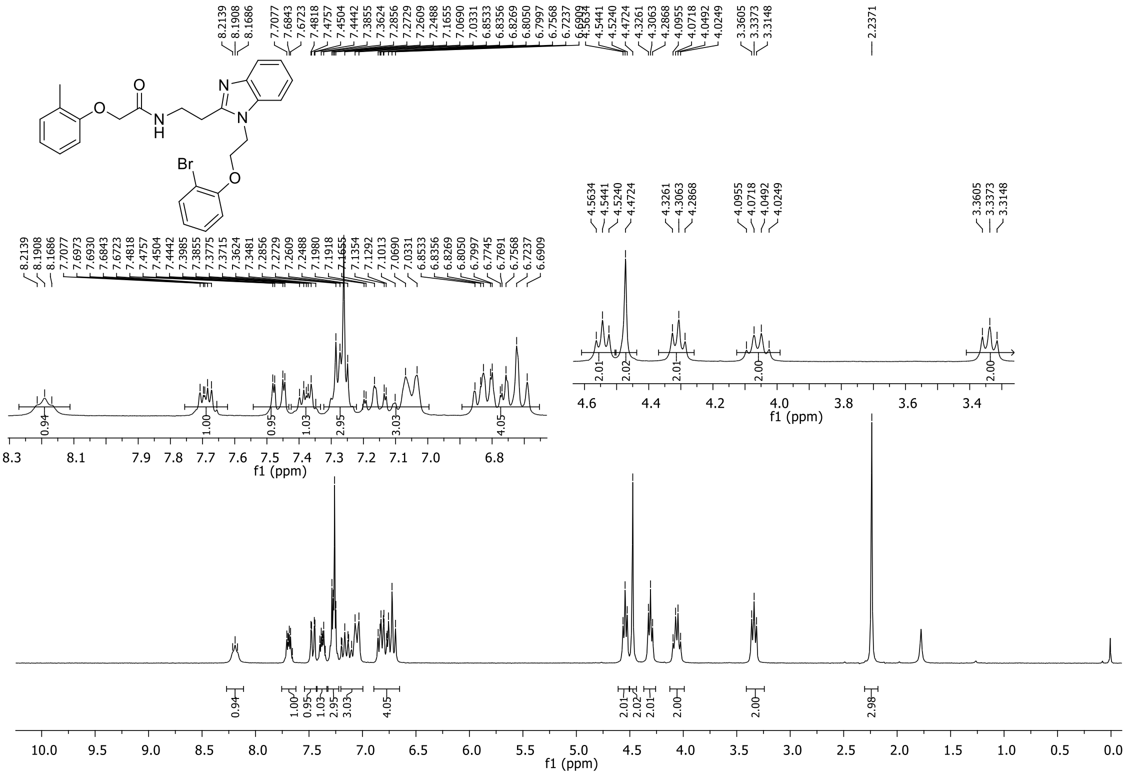

^{1}H NMR of **22** (250 MHz, $CDCl_3$).

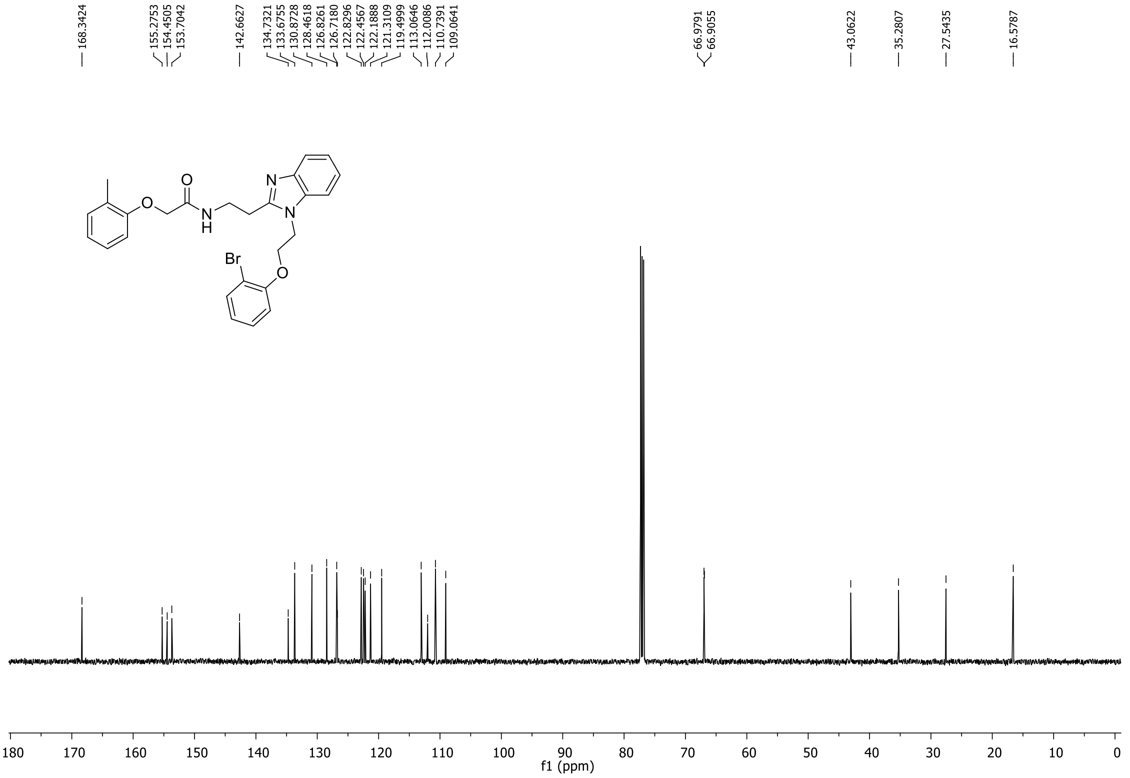

^{13}C NMR of **22** (126 MHz, $CDCl_3$).

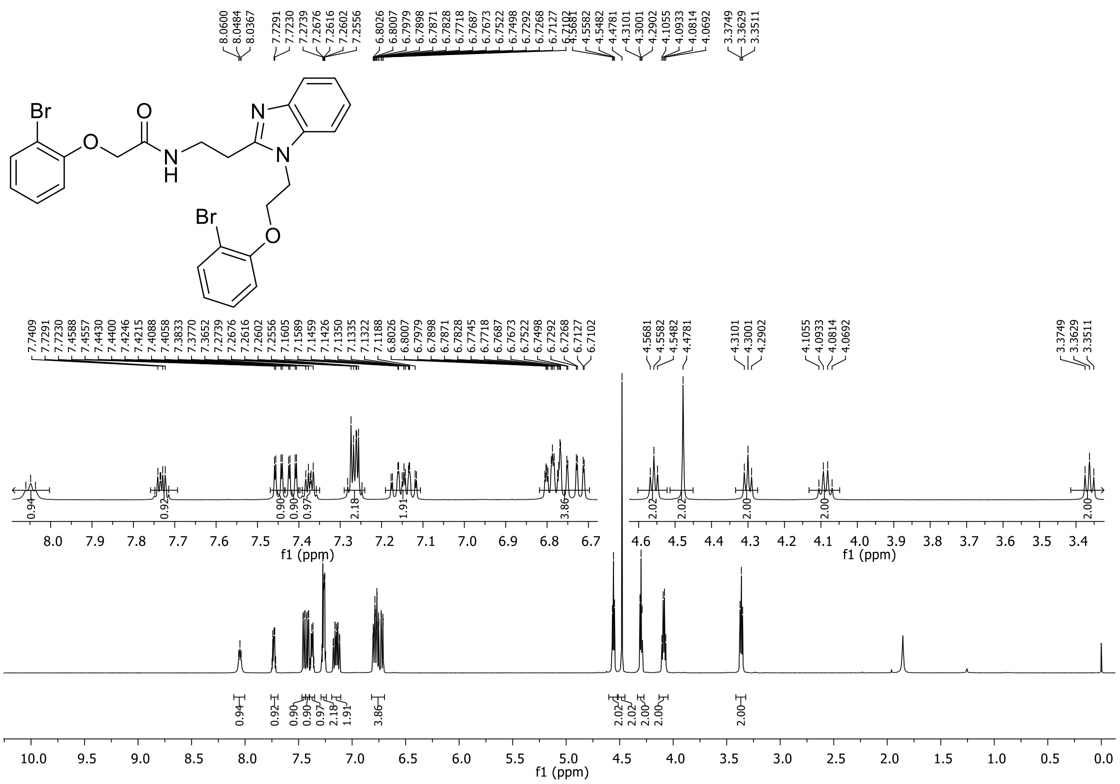

^1H NMR of **23** (500 MHz, $CDCl_3$).

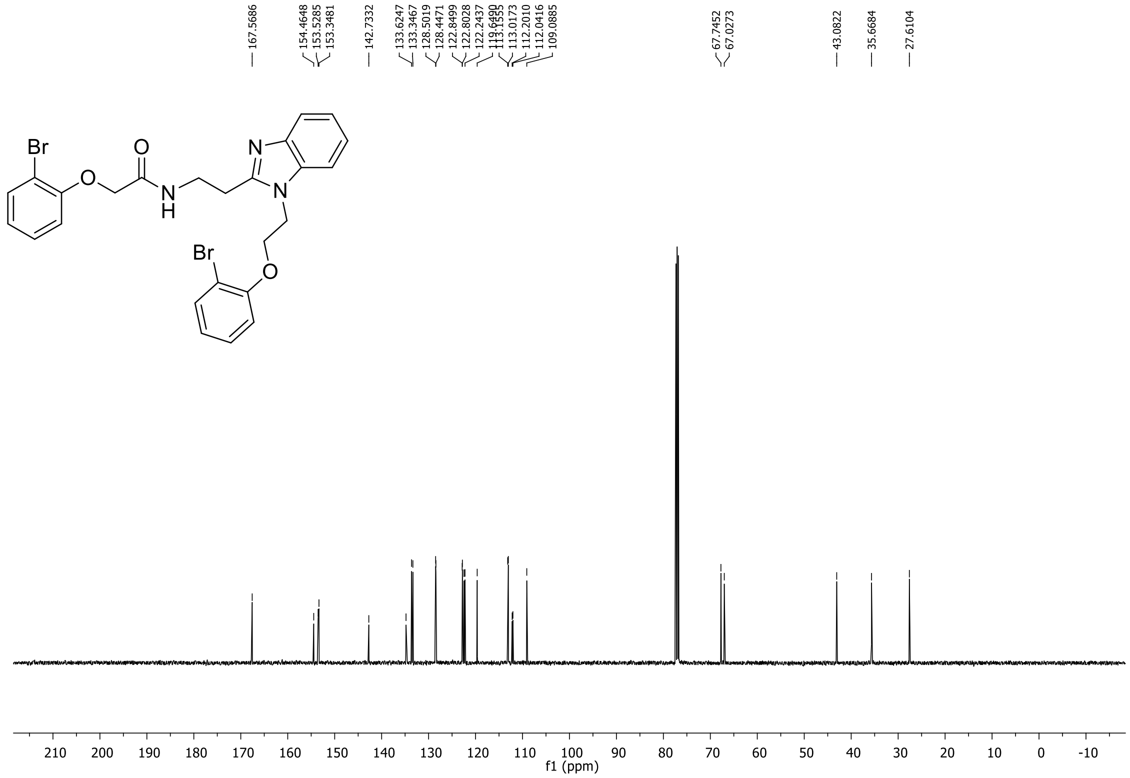

^{13}C NMR of **23** (126 MHz, $CDCl_3$).

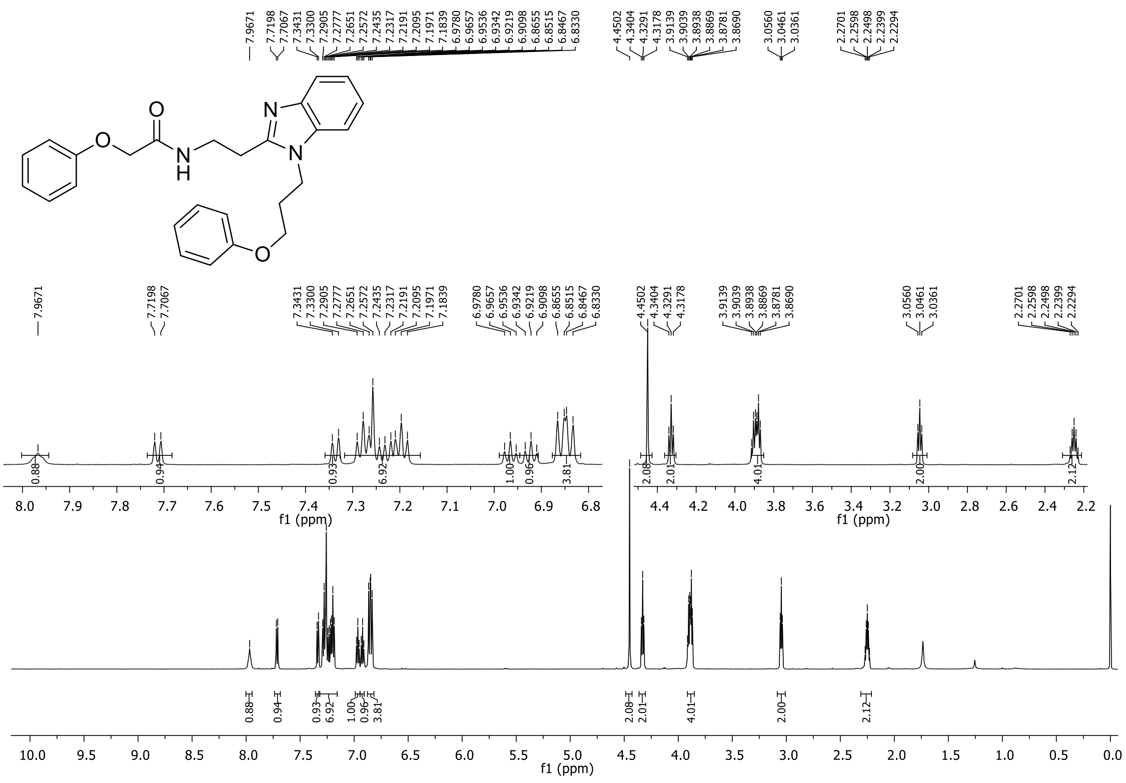

^{1}H NMR of **24** (600 MHz, $CDCl_3$).

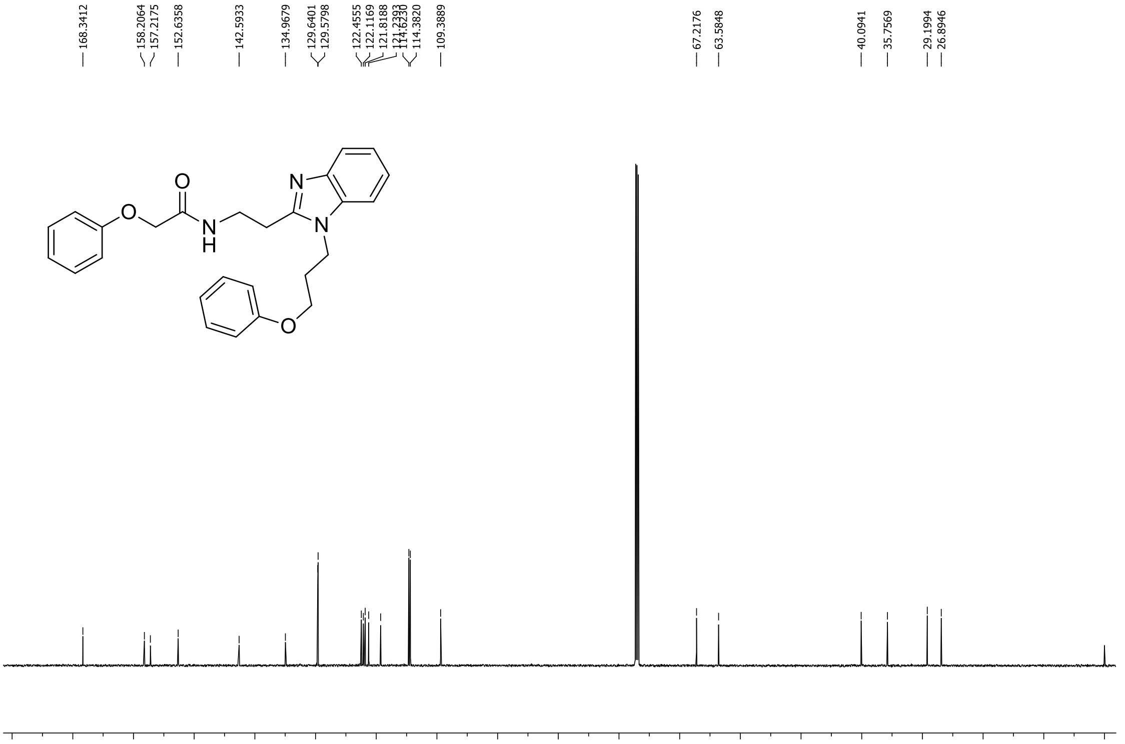

^{13}C NMR of **24** (151 MHz, $CDCl_3$).

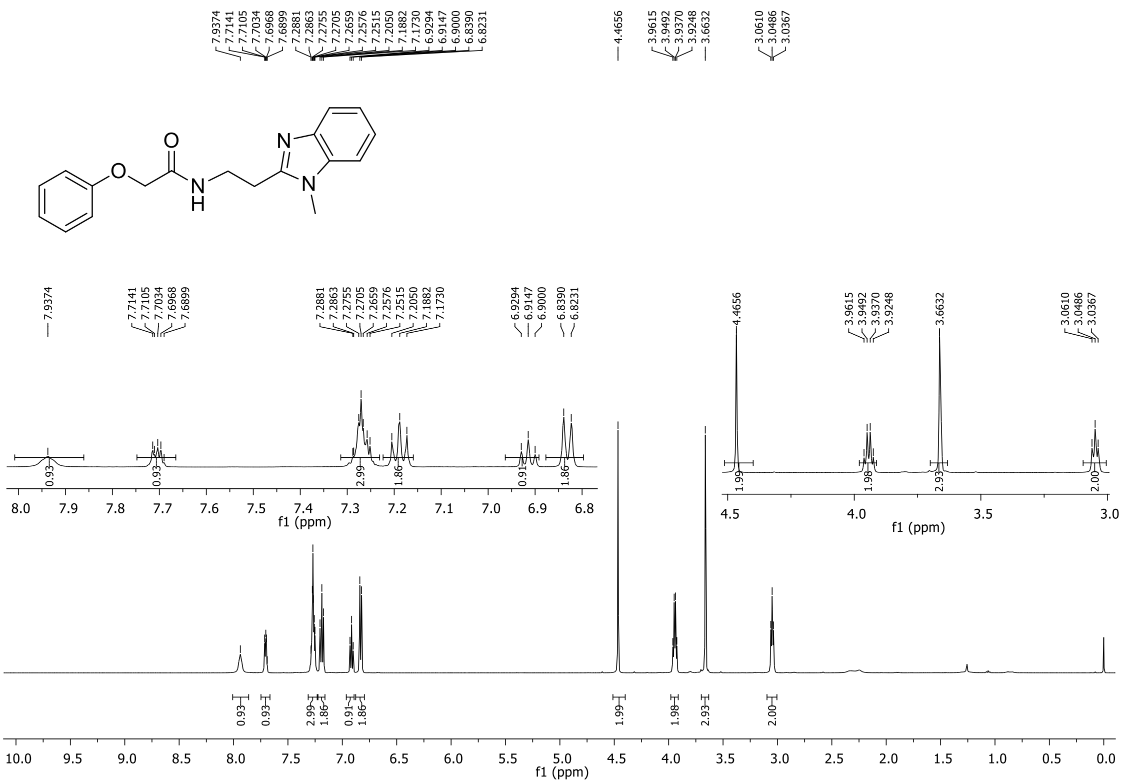

^{1}H NMR of **25** (500 MHz, $CDCl_3$).

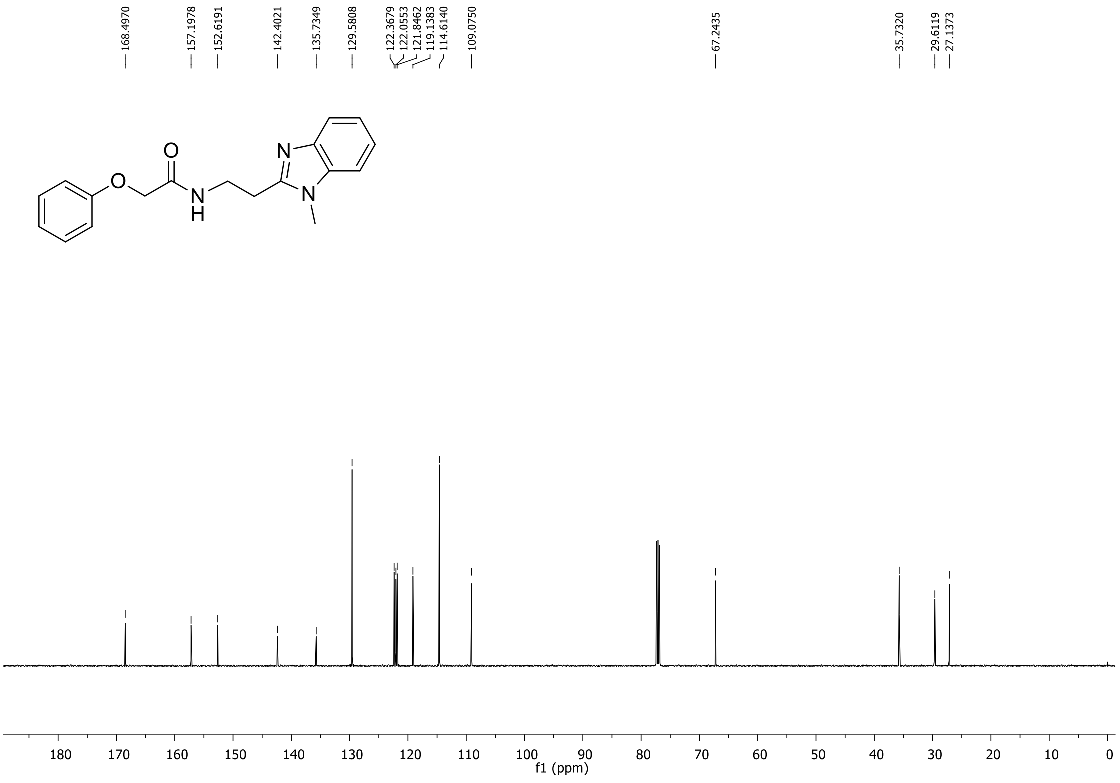

^{13}C NMR of **25** (126 MHz, $CDCl_3$).

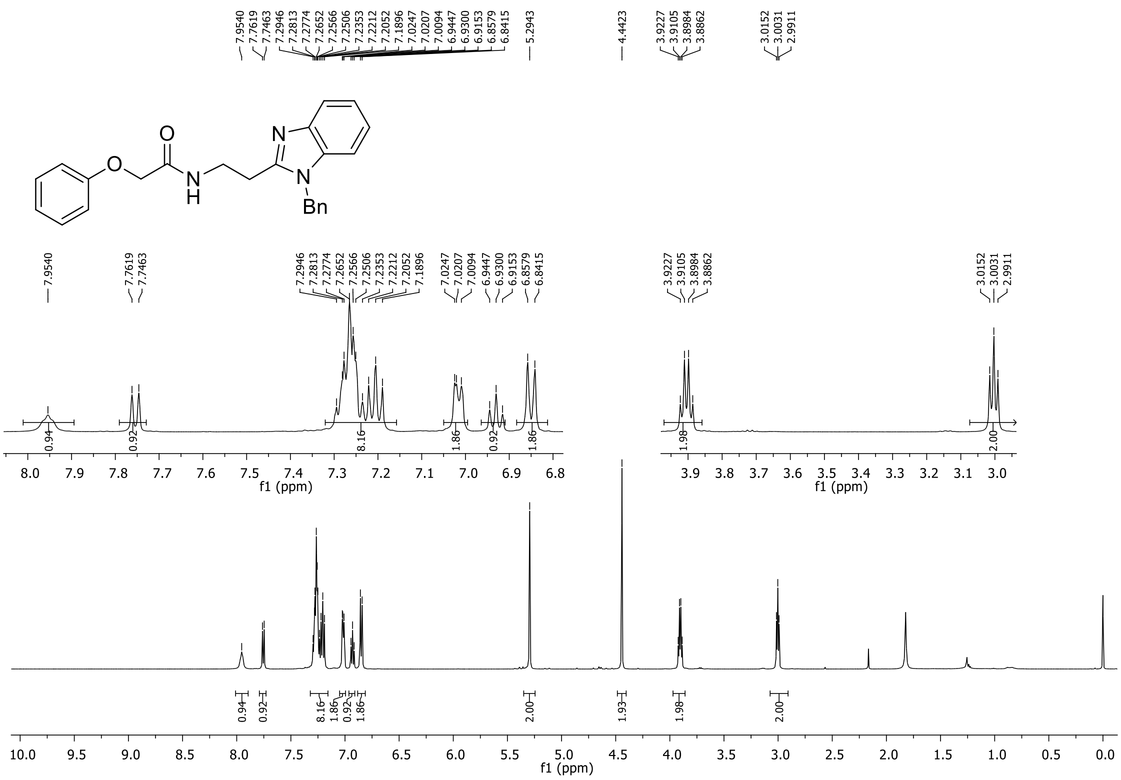

^{1}H NMR of **26** (500 MHz, $CDCl_3$).

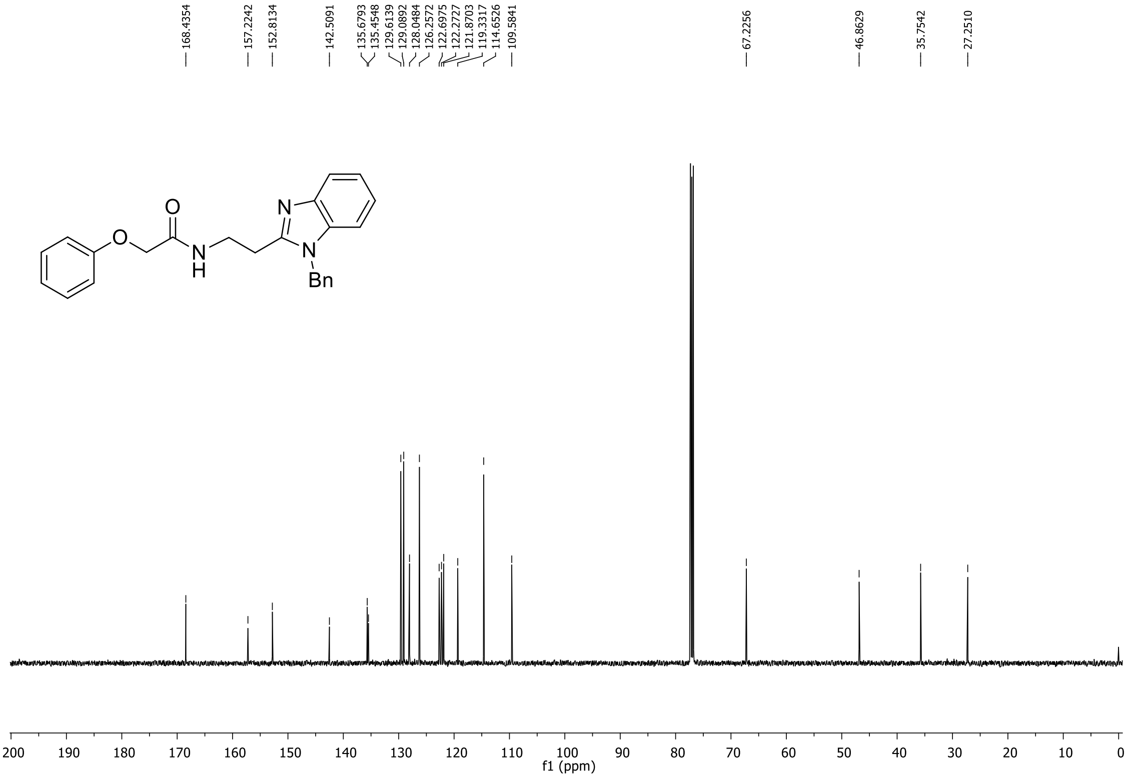

^{13}C NMR of **26** (126 MHz, $CDCl_3$).

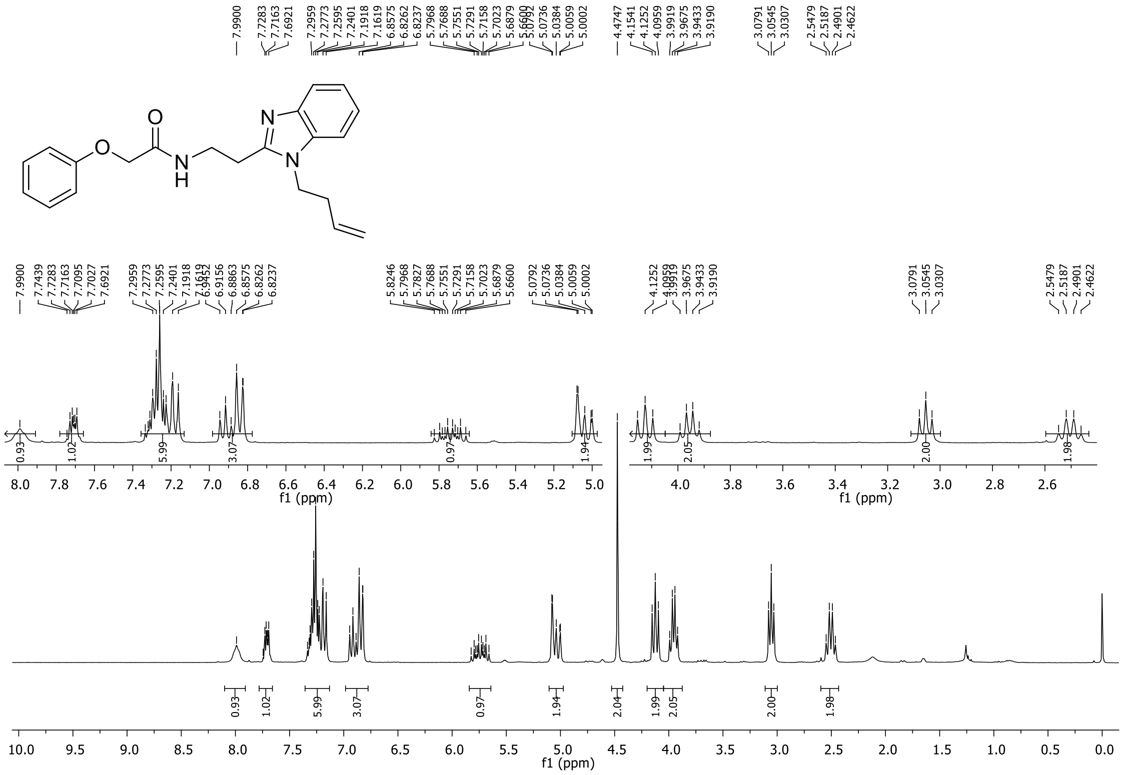

^{1}H NMR of **27** (250 MHz, $CDCl_3$).

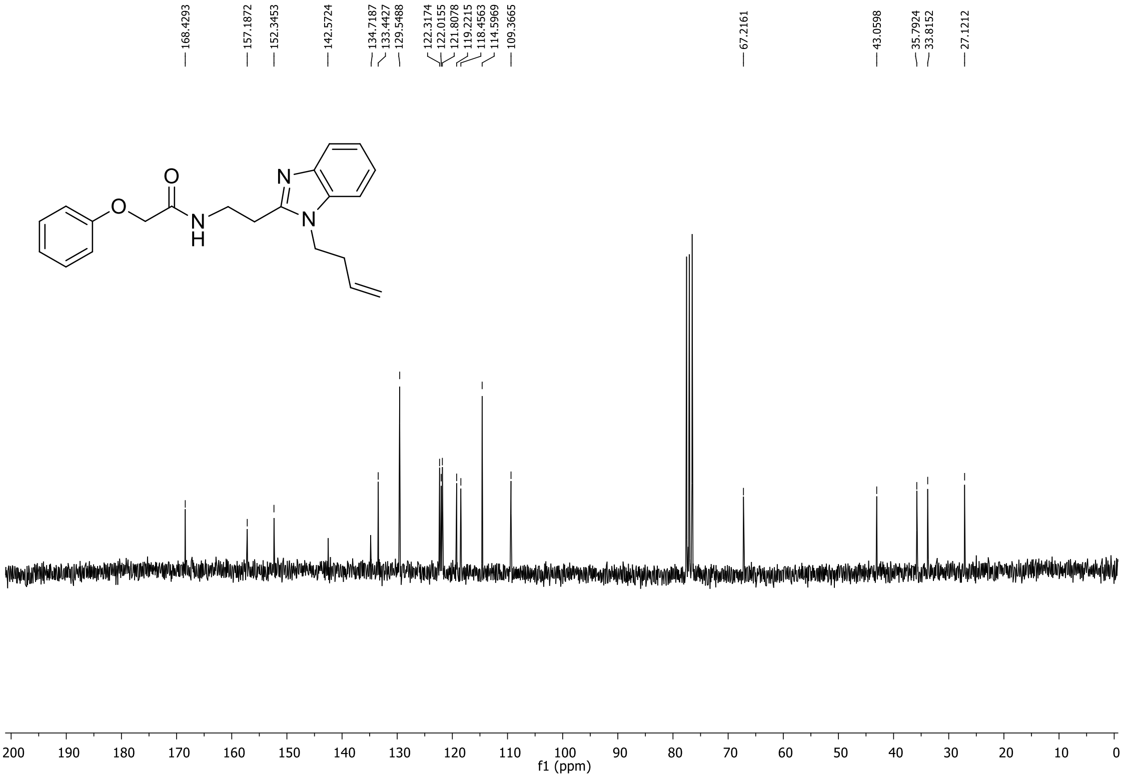

^{13}C NMR of **27** (63 MHz, $CDCl_3$).

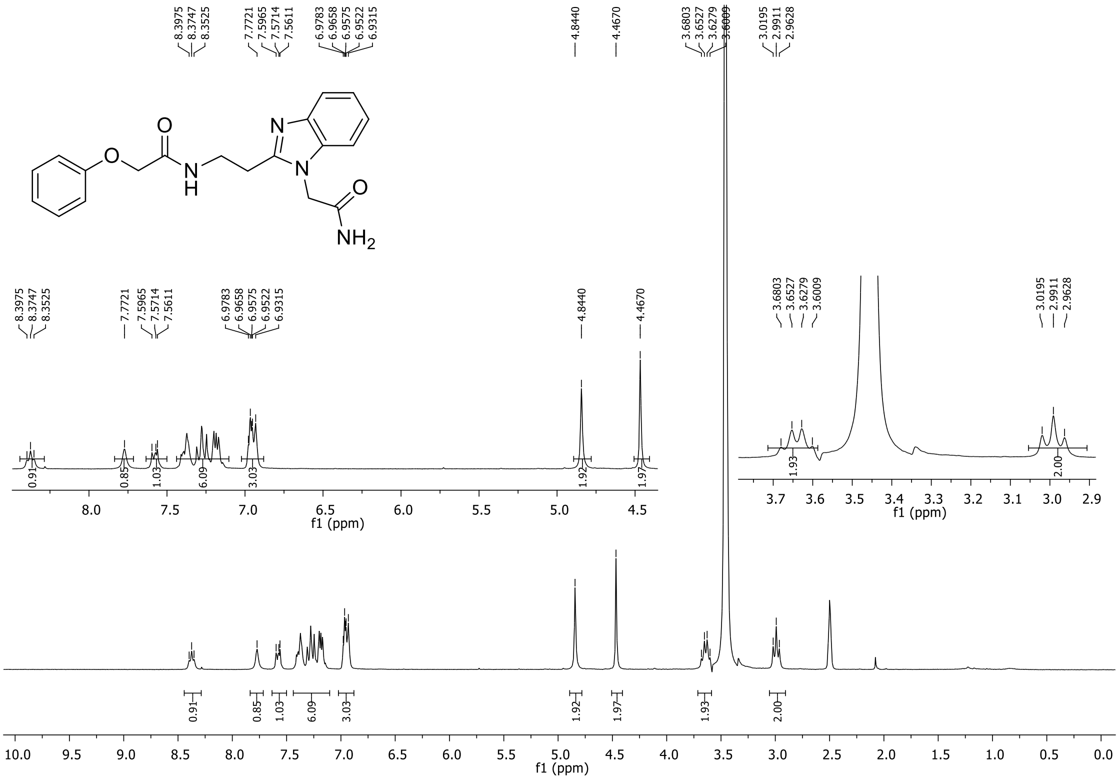

^{1}H NMR of **28** (250 MHz, DMSO).

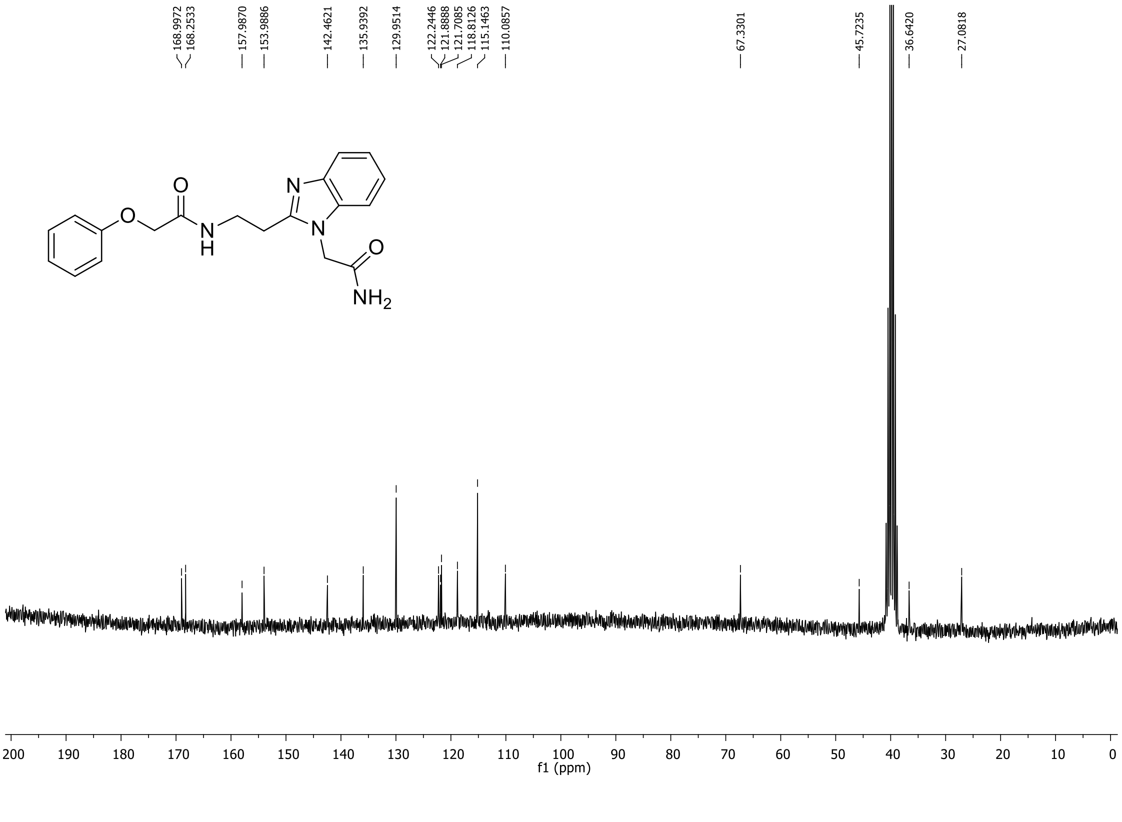

^{13}C NMR of **28** (63 MHz, DMSO).

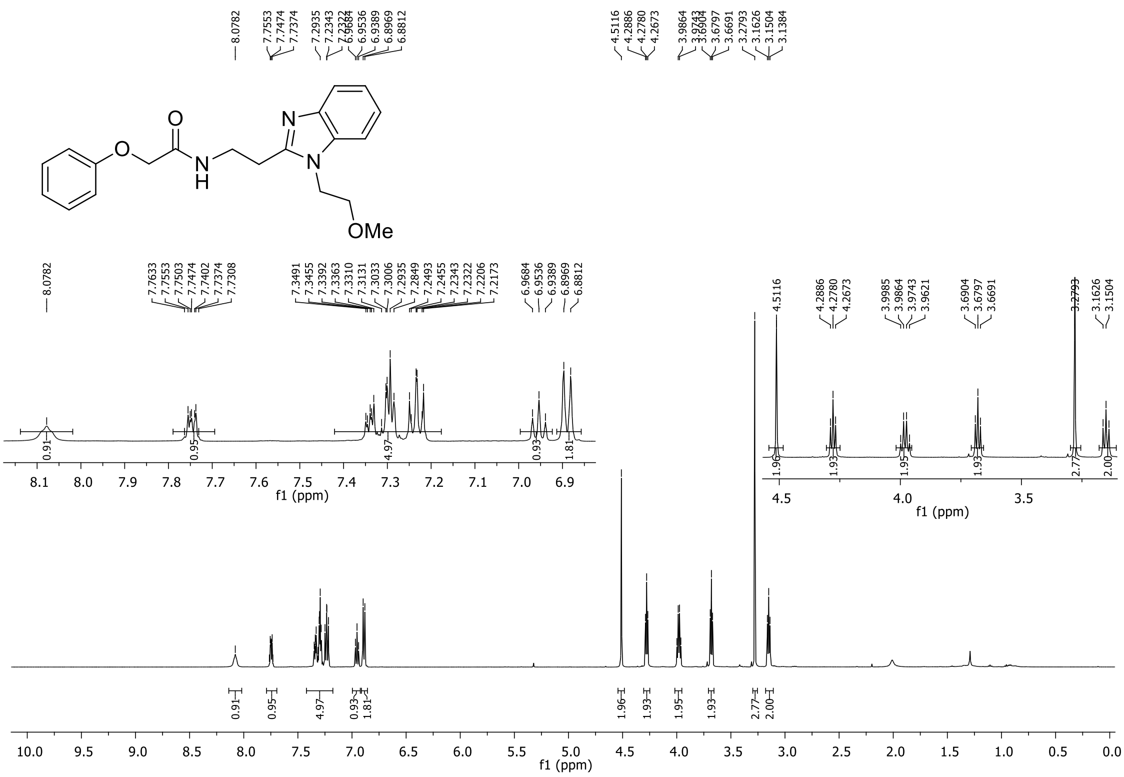

^{1}H NMR **29** (500 MHz, $CDCl_3$).

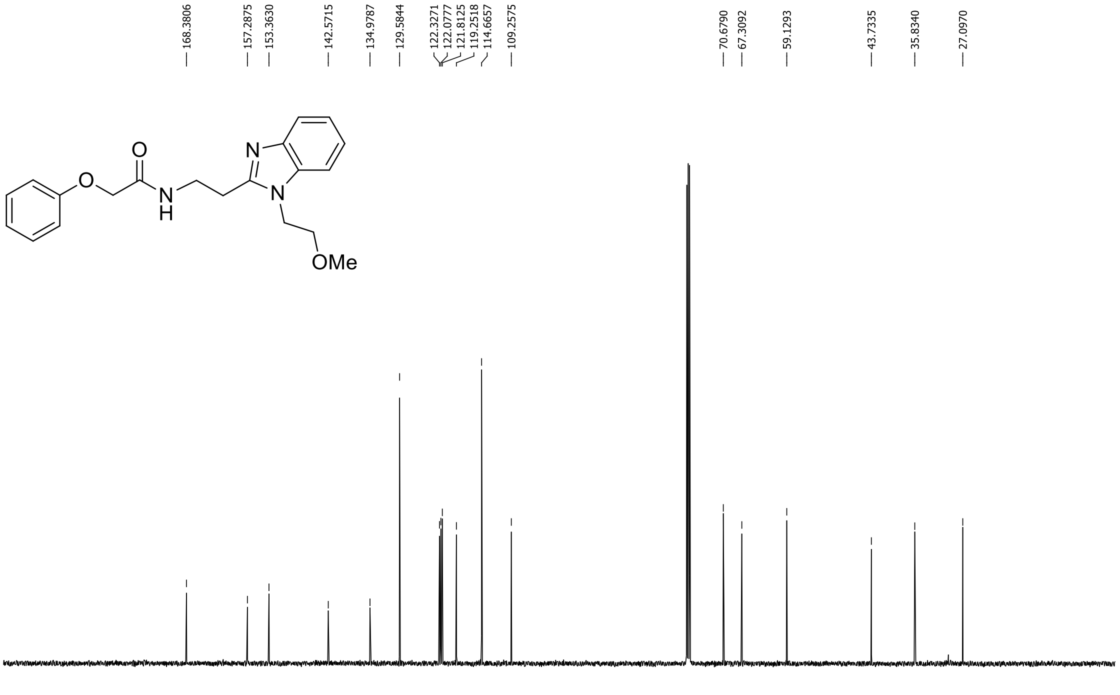

^{13}C NMR of **29** (126 MHz, $CDCl_3$).

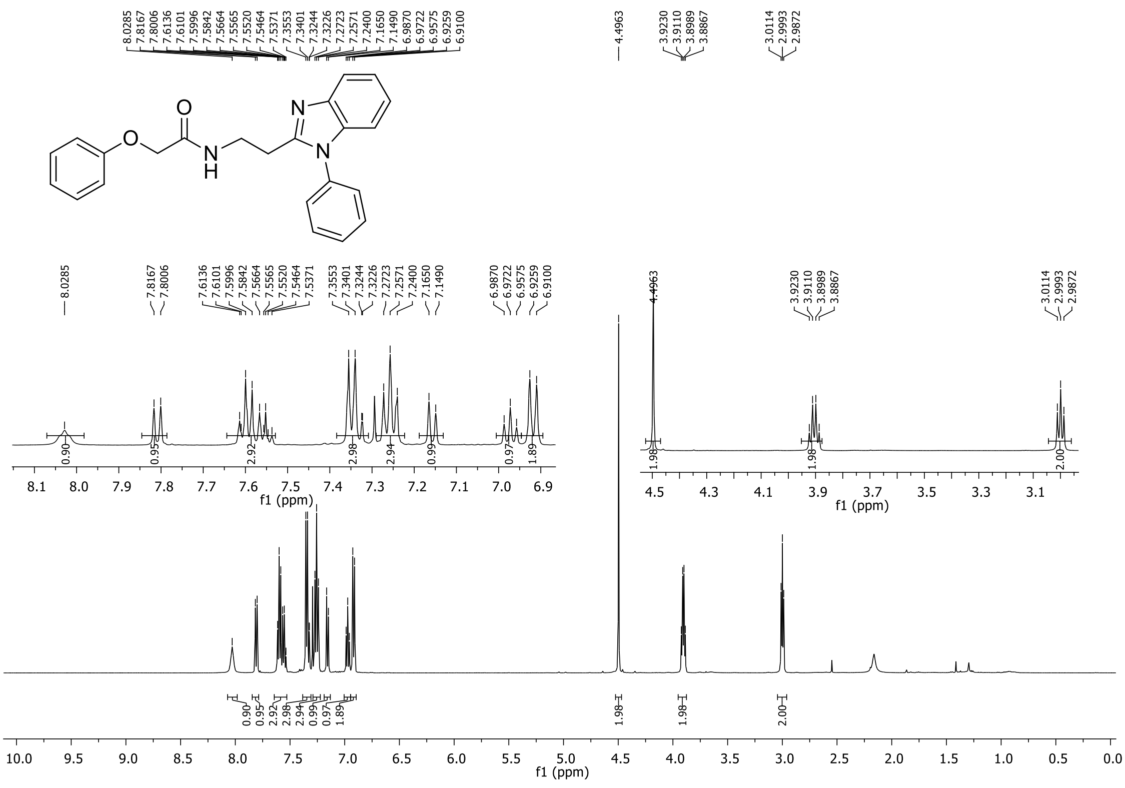

^{1}H NMR of **30** (500 MHz, $CDCl_3$).

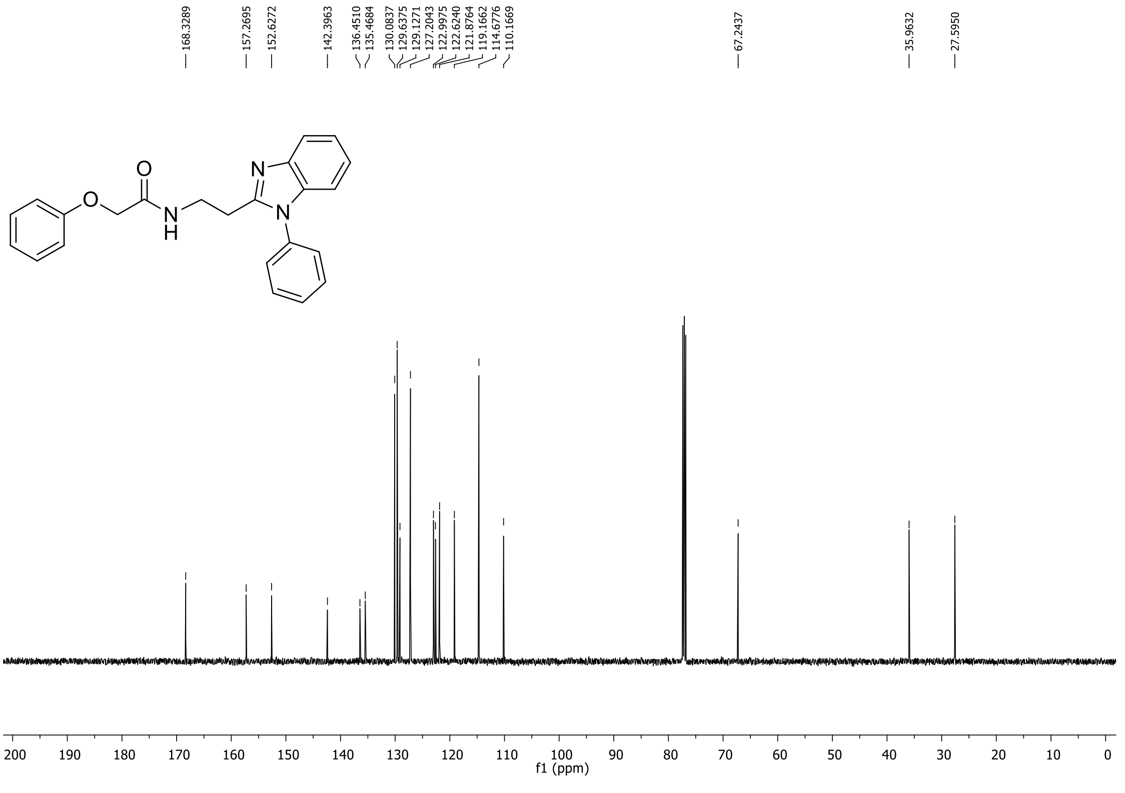

^{13}C NMR of **30** (126 MHz, $CDCl_3$).

Organic Synthesis Methodology

Method A

To a solution of corresponding phenol (1 equiv.) in anhydrous DMF (in a minimum concentration of 0.3 mol. L^{-1}) was added potassium carbonate (2 equiv.) and ethyl 2-bromoacetate (1.2 equiv.). The reaction mixture was stirred at room temperature for 4–6 hours and monitored by TLC. After completion, the reaction mixture was quenched with water and extracted with diethyl ether (2 times). The organic layers were combined, washed with brine, dried over $MgSO_4$ and concentrated under vacuum.

To a solution of the crude material in methanol (at a concentration of 0.5 mol. L^{-1}) was added NaOH (6 mol. L^{-1}, in an equal volume of methanol) at 0 °C. The reaction mixture was stirred at 0 °C for 30 minutes. Hydrochloric acid (6 mol. L^{-1}) was added at 0 °C until pH 2 was reached. The mixture was then stirred at 0 °C for 10 minutes. The solid was filtered and washed with water to yield the corresponding carboxylic acid.

Method B

To a solution of corresponding phenol (1 equiv.) in anhydrous DMF (in a minimum concentration of 0.3 mol. L^{-1}) was added potassium carbonate (2 equiv.) and benzyl 2-bromoacetate (1.2 equiv.). The reaction mixture was stirred at room temperature for 4–6 hours and monitored by TLC. After completion, the reaction mixture was quenched with water and extracted with diethyl ether (2 times). The organic layers were combined, washed with brine, dried with $MgSO_4$ and concentrated under vacuum.

To a solution of crude ethyl acetate/methanol (1:1) (in a concentration of 0.2 mol. L^{-1}) was added 20% Pd/C (10 mol% equiv.) at room temperature. The reaction mixture was stirred at room temperature and under a hydrogen atmosphere for 1–2 hours. After completion, the mixture was filtered with a celite pad and evaporated under vacuum to yield the corresponding carboxylic acid.

Method C

Phenoxyacetic acid (5 mmol) was suspended in absolute dichloromethane (15 mL) at room temperature. A catalytic amount of *N*,*N*-dimethylformamide (0.10 mL) was added, and then neat oxalyl chloride (0.63 g, 5 mmol) was combined portionwise within 10 min. Stirring was continued until gas evolution ceased. The resulting clear solution was cooled down to 0 ºC, solid *N*-hydroxysuccinimide (6 mmol) was combined and then neat dry triethylamine (1.52 g, 15 mmol) was added portionwise within 10 min. After 15 min., the ice bath was removed, and stirring was continued for 2 h. The solvent was evaporated in vacuum, and the solid evaporation residue was partitioned between ethyl acetate and ice-cold water. The organic layer was washed with cold diluted $NaHCO_3$ solution, brine, dried over Na_2SO_4, and concentrated in vacuum. The crude *N*-hydroxysuccinimide ester thus obtained was suspended in diethyl ether (3 mL), and the mixture

was stirred at room temperature (15 min). Hexane (3 mL) was added, and the mixture was kept in a freezer overnight. The solid was filtered off, rinsed with cold diethyl ether, collected, dried in vacuum, and stored. In a separate reaction flask, commercial 2-(2-aminoethyl)benzimidazole dihydrochloride (1.07 mmol) was suspended in ordinary dichloromethane (30 mL). Neat, ordinary triethylamine (3.52 mmol) was added, and to the resulting clear solution was added the matching solid *N*-hydroxysuccinimide ester. The mixture was stirred at room temperature for 15 min. The solvent was evaporated in vacuum, and the evaporation residue was partitioned between ethyl acetate and water. The organic layer was washed with diluted NaOH solution, brine, dried over Na_2SO_4, and concentrated to dryness in vacuum. The crude solid product was suspended in diethyl ether, and the resulting slurry was stirred for 1 h at room temperature. The precipitate was filtered off, rinsed with diethyl ether, collected, and dried in vacuum. Unless otherwise stated, no further purification was required.

Method D

To a solution of 2-(1H-benzo[d]imidazol-2-yl)ethanamine dihydrochloride (**II**) (1 equiv.) in anhydrous DMF was added triethylamine (4 equiv.), followed by the corresponding carboxylic acid (1 equiv.), EDC (1.2 equiv.) and HOBt (1 equiv.) at room temperature. The reaction mixture was stirred at room temperature for 8–15 hours. After completion, the mixture was quenched with water (ten times the volume of DMF). The solid was filtered and washed with water to yield the corresponding amide.

Method E

To a solution of 1*H*-benzo[*d*]imidazole derivative (1 equiv.) in THF (in a minimum concentration of 0.1 mol. L^{-1}) under argon was added 18-c-6 (0.5 equiv.), potassium *tert*-butoxide (1.7 equiv.), and alkyl halide (2–4 equiv.) at room temperature. The reaction mixture was stirred at room temperature or 45 °C for 13–48 hours. The mixture was then poured over water, extracted with EtOAc (3 times), dried over $MgSO_4$, and concentrated under vacuum. Purification by column chromatography ($CHCl_3$/MeOH) yielded the title compound.

Method F

The imide (1 eq) was dissolved in a hot blend of THF/methanol (1:1, v/v, 0.01 mol.L^{-1}). The resulting solution was allowed to cool to room temperature. An excess of solid sodium borohydride (16 eq) was added portionwise within approximately 1.5 h. The reaction progress was monitored by TLC (chloroform/methanol 20:3). After 2 to 3 hours of stirring, excess sodium borohydride was destroyed by the controlled addition of acetic acid in an ice bath. The solvent blend was evaporated under reduced pressure. The dry evaporation residue was partitioned between ethyl acetate (50 mL) and water (20 mL). The organic layer was then washed with diluted

$NaHCO_3$ solution, brine, dried over $MgSO_4$, and concentrated to dryness in vacuum. The solid residue thus obtained was suspended in a blend of ethyl acetate/diethyl ether (1:1, v/v, 10 mL), and the slurry was stirred at room temperature for approximately 2 hours. The solid was filtered off, rinsed with diethyl ether and dried in vacuum. Desired products were obtained as white, amorphous solids in 70 to 85% yield and excellent purity.

1: Prepared as previously described (Ferreira et al., 2014).

2: Method A, followed by C, 71%, white solid; **^{1}H NMR (500 MHz, DMSO)** δ 12.44 (s, 1H), 9.96 (s, 1H), 8.40 (t, *J* = 5.7 Hz, 1H), 7.50 (d, *J* = 9.0 Hz, 4H), 7.20 – 7.06 (m, 2H), 6.89 (d, *J* = 9.0 Hz, 2H), 4.43 (s, 2H), 3.61 (dd, *J* = 13.2, 7.0 Hz, 2H), 3.01 (t, *J* = 7.2 Hz, 2H), 2.01 (s, 3H);

^{13}C NMR (126 MHz, DMSO) δ 168.33, 168.29, 153.84, 153.28, 143.73, 134.88, 133.75, 121.64, 120.90, 118.56, 115.24, 111.39, 67.77, 37.34, 29.10, 24.28;

HRMS for $[C_{19}H_{21}N_4O_3]^+$ $(M + H)^+$ 353.1610, found 353.1588.

3: Method A, followed by D, 45%, white solid; **^{1}H NMR (400 MHz, MeOD)** δ 7.50 (dd, *J* = 5.5, 3.1 Hz, 2H), 7.35 (t, *J* = 2.2 Hz, 1H), 7.24 – 7.18 (m, 2H), 7.15 (t, *J* = 8.2 Hz, 1H), 7.05 (dd, *J* = 8.1, 0.9 Hz, 1H), 6.67 – 6.62 (m, 1H), 4.50 (s, 2H), 3.76 (t, *J* = 7.0 Hz, 2H), 3.14 (t, *J* = 7.0 Hz, 2H), 2.12 (s, 3H);

^{13}C NMR (101 MHz, MeOD) δ 170.28, 169.89, 158.01, 152.58, 139.77, 129.25, 121.90, 112.96, 109.79, 106.51, 66.83, 37.19, 28.41, 22.46;

HRMS for $[C_{19}H_{21}N_4O_3]^+$ $(M + H)^+$ 353.16082, found 353.16089.

4: Method A, followed by C, 44%, white solid; **^{1}H NMR (500 MHz, DMSO)** δ 12.44 (s, 1H), 9.96 (s, 1H), 8.40 (t, *J* = 5.7 Hz, 1H), 7.50 (d, *J* = 9.0 Hz, 4H), 7.20 – 7.06 (m, 2H), 6.89 (d, *J* = 9.0 Hz, 2H), 4.43 (s, 2H), 3.61 (dd, *J* = 13.2, 7.0 Hz, 2H), 3.01 (t, *J* = 7.2 Hz, 2H), 2.01 (s, 3H);

^{13}C NMR (63 MHz, DMSO) δ 177.49, 167.89, 157.59, 153.12, 128.72, 126.27, 121.61, 115.33, 67.66, 37.20, 28.97, 28.81;

HRMS for $[C_{21}H_{21}N_4O_4]^+$ $(M + H)^+$ 393.1559, found 393.1532.

5: Method F, 85%, white solid; **^{1}H NMR (400 MHz, DMSO)** δ 12.27 (s, 1H), 9.76 (s, 1H), 8.35 (t, *J* = 5.8 Hz, 1H), 7.55 (d, *J* = 7.4 Hz, 1H), 7.49 (d, *J* = 9.1 Hz, 2H), 7.42 (d, *J* = 6.5 Hz, 1H), 7.17 – 7.08 (m, 2H), 4.52 (t, *J* = 5.1 Hz, 1H), 4.43 (s, 2H), 3.61 (dd, *J* = 13.1, 7.0 Hz, 2H), 3.43 (dd, *J* = 11.5, 6.3 Hz, 2H), 3.01 (t, *J* = 7.2 Hz, 2H), 2.32 (t, *J* = 7.5 Hz, 2H), 1.79 – 1.67 (m, 2H);

^{13}C NMR (101 MHz, DMSO) δ 171.30, 168.29, 153.82, 153.25, 143.73, 134.70, 133.71, 122.05, 121.39, 120.95, 118.63, 115.27, 111.32, 67.81, 60.72, 37.34, 33.48, 29.12, 28.98;

HRMS for $[C_{21}H_{25}N_4O_4]^+$ $(M + H)^+$ 397.1872, found 397.1847.

6: Method A, followed by C, 57%, white solid; **^{1}H NMR (400 MHz, DMSO)** δ 12.28 (s, 1H), 8.43 (t, *J* = 5.5 Hz, 1H), 7.48 (s, 2H), 7.37 (t, *J* = 8.1 Hz, 1H), 7.13 (dd, *J* = 5.9, 3.1 Hz, 2H), 6.98

(dd, *J* = 8.3, 2.0 Hz, 1H), 6.95 – 6.84 (m, 2H), 4.49 (s, 2H), 3.61 (dd, *J* = 13.1, 6.8 Hz, 2H), 3.01 (t, *J* = 7.1 Hz, 2H), 2.77 (s, 4H);

^{13}C NMR (63 MHz, DMSO) δ 177.18, 167.94, 158.25, 153.12, 134.25, 129.90, 121.50, 120.54, 114.76, 67.45, 37.25, 29.00, 28.88;

HRMS for $[C_{21}H_{21}N_4O_4]^+$ $(M + H)^+$ 393.1559, found 393.1539.

7: Method F, 67%, white solid; **^{1}H NMR (250 MHz, DMSO)** ∑ 1.7 (qd, *J*=7.0, 6.8 Hz, 2 H) 2.3 (t, *J*=7.4 Hz, 2 H) 3.0 (t, *J*=7.1 Hz, 2 H) 3.4 (q, *J*=6.1 Hz, 2 H) 3.6 (m, 2 H) 4.4 (s, 2 H) 4.5 (t, *J*=5.1 Hz, 1 H) 6.6 (m, 1 H) 7.1 (m, 4 H) 7.4 (s, 2 H) 7.5 (s, 1 H) 8.4 (m, 1 H) 9.9 (s, 1 H) 12.2 (s, 1 H);

^{13}C NMR (63 MHz, DMSO) δ 171.81, 167.94, 158.34, 153.22, 143.80, 140.95, 134.56, 129.83, 121.99, 121.33, 118.63, 112.45, 111.26, 109.23, 106.37, 67.34, 60.61, 37.31, 33.60, 29.07, 28.82;

HRMS for $[C_{21}H_{25}N_4O_4]^+$ $(M + H)^+$ 397.1872, found 397.1852.

8: Method A, followed by D, 56%, white solid; **^{1}H NMR (400 MHz, DMSO)** δ 12.27 (s, 1H), 8.48 (t, *J* = 5.6 Hz, 1H), 7.82 (dd, *J* = 8.1, 1.6 Hz, 1H), 7.77 (t, *J* = 2.2 Hz, 1H), 7.61 – 7.35 (m, 4H), 7.12 (dd, *J* = 6.0, 2.9 Hz, 2H), 4.66 (s, 2H), 3.62 (dd, *J* = 13.1, 7.0 Hz, 2H), 3.01 (t, *J* = 7.2 Hz, 2H);

^{13}C NMR (101 MHz, DMSO) δ 167.52, 158.64, 153.14, 149.03, 131.17, 122.16, 118.70, 116.47, 111.32, 110.07, 67.75, 37.38, 29.10;

HRMS for $[C_{17}H_{17}N_4O_4]^+$ $(M + H)^+$ 341.12443, found 341.12450.

To a solution of **8** in methanol (in a concentration of 0.2 mol. L^{-1}) was added 20% Pd/C (10 mol% equiv.) at room temperature. The reaction mixture was stirred at room temperature and under a hydrogen atmosphere for 2 hours. After completion, the mixture was filtered with a celite pad and evaporated under vacuum to give **9** in 94% yield.

9: White solid; **^{1}H NMR (500 MHz, MeOD)** δ 7.53 (dd, *J* = 6.0, 3.2 Hz, 2H), 7.23 (dd, *J* = 6.0, 3.1 Hz, 2H), 6.95 (t, *J* = 8.0 Hz, 1H), 6.36 – 6.34 (m, 1H), 6.33 (t, *J* = 2.2 Hz, 1H), 6.25 (dd, *J* = 8.1, 2.1 Hz, 1H), 4.44 (s, 2H), 3.77 (t, *J* = 7.0 Hz, 2H), 3.15 (t, *J* = 7.0 Hz, 2H);

^{13}C NMR (126 MHz, MeOD) δ 170.30, 158.73, 152.58, 148.98, 129.56, 121.99, 114.11, 108.96, 103.82, 101.58, 66.75, 48.12, 47.95, 47.82, 47.78, 47.75, 47.61, 47.44, 47.40, 47.27, 47.10, 37.15, 28.42;

HRMS for $[C_{17}H_{19}N_4O_2]^+$ $(M + H)^+$ 311.15025, found 311.15010.

10: Method A, followed by D, 37%, white solid; **^{1}H NMR (400 MHz, DMSO)** δ 12.28 (s, 1H), 9.51 (s, 1H), 8.44 (t, *J* = 5.8 Hz, 1H), 7.61 (dd, *J* = 8.8, 4.9 Hz, 2H), 7.56 (d, *J* = 6.7 Hz, 1H), 7.42 (d, *J* = 6.2 Hz, 1H), 7.19 (d, *J* = 2.4 Hz, 1H), 7.17 – 7.11 (m, *J* = 8.8, 4.3, 2.4 Hz, 3H), 7.08

(d, J = 2.4 Hz, 1H), 7.04 (dd, J = 8.8, 2.4 Hz, 1H), 4.55 (s, 2H), 3.64 (dd, J = 13.1, 7.2 Hz, 2H), 3.04 (t, J = 7.2 Hz, 2H);

^{13}C NMR (101 MHz, DMSO) δ 168.25, 154.27, 153.82, 153.27, 143.74, 134.70, 130.61, 128.72, 128.62, 127.98, 122.04, 121.39, 119.42, 119.13, 118.63, 111.32, 109.31, 108.12, 67.62, 37.33, 29.12;

HRMS for $[C_{21}H_{20}N_3O_3]^+$ $(M + H)^+$ 362.14992, found 362.14976.

11: Method A, followed by D, 28%, white solid; **^{1}H NMR (500 MHz, DMSO)** δ 9.71 (s, 1H), 8.47 (t, J = 5.4 Hz, 1H), 7.68 (t, J = 9.0 Hz, 2H), 7.55 (dd, J = 5.4, 3.2 Hz, 2H), 7.21 (dd, J = 6.0, 3.1 Hz, 2H), 7.09 (d, J = 2.2 Hz, 1H), 7.03 (s, 1H), 6.96 (ddd, J = 27.8, 8.8, 2.3 Hz, 2H), 4.58 (s, 2H), 3.66 (dd, J = 13.1, 6.8 Hz, 2H), 3.37 (s, 1H), 3.09 (t, J = 7.0 Hz, 2H);

^{13}C NMR (126 MHz, DMSO) δ 168.26, 156.40, 156.36, 153.17, 136.28, 129.60, 129.54, 123.87, 122.47, 116.80, 115.52, 114.84, 108.63, 106.45, 67.46, 37.27, 28.85;

HRMS for $[C_{21}H_{20}N_3O_3]^+$ $(M + H)^+$ 362.14992, found 362.14934.

12: Method B, followed by D, 78%, white solid; **^{1}H NMR (500 MHz, DMSO)** δ 12.30 (s, 1H), 8.57 (s, 1H), 8.51 (t, J = 5.5 Hz, 1H), 8.08 (d, J = 9.0 Hz, 1H), 7.95 (d, J = 9.0 Hz, 1H), 7.87 (d, J = 9.0 Hz, 1H), 7.56 (s, 1H), 7.41 (d, J = 14.7 Hz, 2H), 7.35 (dd, J = 9.0, 1.9 Hz, 1H), 7.14 (d, J = 4.1 Hz, 2H), 4.68 (s, 2H), 3.92 (s, 3H), 3.65 (dd, J = 12.9, 7.1 Hz, 2H), 3.04 (t, J = 7.1 Hz, 2H);

^{13}C NMR (126 MHz, DMSO) δ 167.79, 166.89, 158.07, 153.25, 143.69, 137.08, 134.73, 131.55, 130.84, 128.20, 127.73, 125.91, 125.33, 122.04, 121.39, 120.12, 118.65, 111.33, 107.78, 67.50, 52.58, 37.40, 29.12;

HRMS for $[C_{23}H_{22}N_3O_4]^+$ $(M + H)^+$ 404.16048, found 404.16049.

To a solution of **12** (1 mmol) in methanol (4 mL) was added a solution of NaOH (6 mol. L^{-1}, 4 mL) at room temperature. The reaction mixture was stirred for 20 minutes, and HCl (6 mol. L^{-1}) was added at 0 ºC until pH 2 was reached. The solid was filtered and washed with water to give carboxylic acid **13** in 82% yield.

13: White solid; **^{1}H NMR (500 MHz, MeOD)** δ 8.50 (s, 1H), 8.00 (dd, J = 8.6, 1.3 Hz, 1H), 7.88 (d, J = 9.0 Hz, 1H), 7.77 (d, J = 8.6 Hz, 1H), 7.69 (dd, J = 6.1, 3.1 Hz, 2H), 7.54 (dd, J = 6.1, 3.1 Hz, 2H), 7.30 (dd, J = 9.0, 2.5 Hz, 1H), 7.26 (d, J = 2.0 Hz, 1H), 4.68 (s, 2H), 3.89 (t, J = 6.3 Hz, 2H), 3.46 (t, J = 6.3 Hz, 2H);

^{13}C NMR (126 MHz, MeOD) δ 170.14, 168.61, 157.33, 151.71, 136.89, 130.99, 130.81, 130.54, 128.36, 126.77, 126.06, 125.92, 125.80, 119.03, 113.30, 106.76, 66.66, 36.45, 27.05;

HRMS calcd for $[C_{22}H_{20}N_3O_4]^+$ $(M + H)^+$ 390.14483, found 390.14431.

14: Method B, followed by D, 85%, white solid; **^{1}H NMR (600 MHz, MeOD)** δ 8.75 (d, J = 9.4 Hz, 1H), 8.03 (d, J = 6.5 Hz, 1H), 7.91 (d, J = 8.2 Hz, 1H), 7.53 – 7.42 (m, 3H), 7.30 (dd, J =

9.4, 2.6 Hz, 1H), 7.25 (d, *J* = 2.5 Hz, 1H), 7.18 (dd, *J* = 6.0, 3.1 Hz, 2H), 4.65 (s, 2H), 3.99 (s, 3H), 3.79 (t, *J* = 6.9 Hz, 2H), 3.15 (t, *J* = 6.9 Hz, 2H);

^{13}C NMR (151 MHz, MeOD) δ 169.72, 168.02, 155.48, 152.51, 135.20, 132.09, 128.77, 127.95, 127.85, 127.17, 126.96, 126.76, 124.98, 121.90, 119.60, 107.59, 66.73, 51.20, 37.13, 28.36;

HRMS for $[C_{23}H_{22}N_3O_4]^+$ $(M + H)^+$ 404.16048, found 404.16035.

To a solution of **14** (1 mmol) in methanol (4 mL) was added a solution of NaOH (6 mol. L^{-1}, 4 mL) at room temperature. The reaction mixture was stirred for 20 minutes, and HCl (6 mol. L^{-1}) was added at 0 ºC until pH 2 was reached. The solid was filtered and washed with water to give carboxylic acid **15** in 79% yield.

15: White solid; **^{1}H NMR (500 MHz, MeOD)** δ 8.82 (d, *J* = 9.4 Hz, 1H), 8.08 (d, *J* = 7.2 Hz, 1H), 7.91 (d, *J* = 8.1 Hz, 1H), 7.70 – 7.63 (m, *J* = 5.7, 2.9 Hz, 2H), 7.54 – 7.46 (m, 3H), 7.29 (d, *J* = 9.4 Hz, 1H), 7.25 (s, 1H), 4.65 (s, 2H), 3.87 (t, *J* = 5.9 Hz, 2H), 3.44 (t, *J* = 5.9 Hz, 2H);

^{13}C NMR (126 MHz, MeOD) δ 170.34, 169.51, 155.38, 151.68, 135.20, 131.82, 131.07, 128.11, 127.53, 127.16, 125.89, 125.77, 125.08, 119.21, 113.30, 107.78, 66.78, 48.13, 47.96, 47.79, 47.62, 47.45, 47.28, 47.11, 36.41, 27.06;

HRMS for $[C_{22}H_{20}N_3O_4]^+$ $(M + H)^+$ 390.14483, found 390.14464.

16: Method A, followed by method D, 73%, brown solid; **^{1}H NMR (500 MHz, DMSO)** δ 12.29 (s, 1H), 8.45 (t, *J* = 5.7 Hz, 1H), 7.92 (d, *J* = 9.1 Hz, 1H), 7.49 (d, *J* = 32.5 Hz, 2H), 7.14 (dd, *J* = 5.9, 2.9 Hz, 2H), 6.84 (d, *J* = 2.4 Hz, 1H), 6.64 (dd, *J* = 9.1, 2.5 Hz, 1H), 6.54 (d, *J* = 52.2 Hz, 1H), 4.67 (s, 2H), 3.88 (s, 3H), 3.64 (dd, *J* = 13.1, 6.9 Hz, 2H), 3.03 (t, *J* = 7.1 Hz, 2H);

^{13}C NMR (126 MHz, DMSO) δ 167.33, 163.28, 155.32, 153.17, 143.81, 133.10, 128.20, 122.05, 121.28, 118.55, 111.27, 106.76, 101.14, 67.76, 57.17, 40.50, 40.43, 40.34, 40.26, 40.17, 40.09, 40.00, 39.84, 39.67, 39.50, 37.41, 29.12;

HRMS for $[C_{18}H_{19}N_4O_5]^+$ $(M + H)^+$ 371.13500, found 371.13507.

17: Prepared as previously described (Ferreira et al., 2014).

18: Prepared as previously described (Ferreira et al., 2014).

19: Method E, 86%, white solid; **^{1}H NMR (500 MHz, $CDCl_3$)** δ 8.11 (t, *J* = 5.1 Hz, 1H), 7.77 – 7.69 (m, 1H), 7.47 (dd, *J* = 7.9, 1.5 Hz, 1H), 7.42 – 7.36 (m, 1H), 7.32 – 7.24 (m, 2H), 7.21 – 7.15 (m, 3H), 6.90 (t, *J* = 7.4 Hz, 1H), 6.85 (d, *J* = 7.9 Hz, 2H), 6.81 (td, *J* = 7.7, 1.2 Hz, 1H), 6.74 (dd, *J* = 8.3, 1.1 Hz, 1H), 4.59 – 4.52 (m, 2H), 4.47 (s, 2H), 4.29 (t, *J* = 5.0 Hz, 2H), 4.00 (q, *J* = 6.1 Hz, 2H), 3.34 (t, *J* = 6.0 Hz, 2H);

^{13}C NMR (126 MHz, $CDCl_3$) δ 168.36, 157.27, 154.47, 153.54, 142.70, 134.73, 133.68, 129.57, 128.48, 122.83, 122.49, 122.27, 121.77, 119.44, 114.67, 113.10, 112.02, 109.14, 67.28, 66.98, 43.07, 35.66, 27.37;

HRMS for $[C_{25}H_{25}N_3O_3]^+$ $(M + H)^+$ 494.1075, found 494.1050.

20: Method E, 54%, white solid; **^{1}H NMR (600 MHz, $CDCl_3$)** δ 8.03 (s, 1H), 7.74 – 7.68 (m, 1H), 7.41 – 7.34 (m, 1H), 7.30 – 7.25 (m, 2H), 7.22 (dd, *J* = 8.6, 7.5 Hz, 2H), 7.17 (dd, *J* = 8.5, 7.5 Hz, 2H), 6.90 (dt, *J* = 19.4, 7.3 Hz, 2H), 6.83 (d, *J* = 7.9 Hz, 2H), 6.77 (d, *J* = 8.0 Hz, 2H), 4.51 – 4.44 (m, 4H), 4.24 (t, *J* = 5.2 Hz, 2H), 3.99 (q, *J* = 6.1 Hz, 2H), 3.20 (t, *J* = 6.0 Hz, 2H);

^{13}C NMR (151 MHz, $CDCl_3$) δ 168.46, 157.85, 157.21, 153.16, 142.57, 134.93, 129.60, 129.58, 129.55, 122.49, 122.24, 121.78, 121.53, 119.37, 114.61, 114.26, 109.21, 67.24, 65.83, 43.17, 35.79, 27.21;

HRMS for $[C_{25}H_{26}N_3O_3]^+$ $(M + H)^+$ 416.1941, found 416.1941.

21: Method E, 77%, white solid; **^{1}H NMR (250 MHz, $CDCl_3$)** δ 8.02 (t, *J* = 4.8 Hz, 1H), 7.77 – 7.66 (m, 1H), 7.34 – 7.01 (m, 10H), 6.96 – 6.81 (m, 3H), 4.45 (s, 2H), 4.39 (s, 2H), 4.27 (t, *J* = 5.3 Hz, 2H), 3.91 (q, *J* = 6.1 Hz, 2H), 3.73 (t, *J* = 5.3 Hz, 2H), 3.10 (t, *J* = 6.1 Hz, 2H);

^{13}C NMR (151 MHz, $CDCl_3$) δ 168.37, 157.26, 153.34, 142.52, 137.36, 134.95, 129.58, 128.43, 127.82, 127.43, 122.33, 122.08, 121.81, 119.21, 114.65, 109.34, 73.32, 67.93, 67.25, 43.76, 35.77, 27.14.

22: Method E, 87%, white solid; **^{1}H NMR (250 MHz, $CDCl_3$)** δ 8.19 (t, *J* = 5.7 Hz, 1H), 7.76 – 7.62 (m, 1H), 7.46 (dd, *J* = 7.9, 1.6 Hz, 1H), 7.42 – 7.33 (m, 1H), 7.32 – 7.22 (m, 3H), 7.20 – 7.00 (m, 3H), 6.89 – 6.65 (m, 4H), 4.54 (t, *J* = 4.9 Hz, 2H), 4.47 (s, 2H), 4.31 (t, *J* = 4.9 Hz, 2H), 4.06 (dd, *J* = 11.7, 6.0 Hz, 2H), 3.34 (t, *J* = 5.7 Hz, 2H), 2.24 (s, 3H);

^{13}C NMR (126 MHz, $CDCl_3$) δ 168.34, 155.28, 154.45, 153.70, 142.66, 134.73, 133.68, 130.87, 128.46, 126.83, 126.72, 122.83, 122.46, 122.19, 121.31, 119.50, 113.06, 112.01, 110.74, 109.06, 66.98, 66.91, 43.06, 35.28, 27.54, 16.58;

HRMS for $[C_{26}H_{27}BrN_3O_3]^+$ $(M + H)^+$ 508.1232, found 508.1197.

23: Method E, 85%, white solid; **^{1}H NMR (500 MHz, $CDCl_3$)** δ 8.05 (t, *J* = 5.8 Hz, 1H), 7.76 – 7.69 (m, 1H), 7.45 (dd, *J* = 7.9, 1.5 Hz, 1H), 7.42 (dd, *J* = 7.9, 1.5 Hz, 1H), 7.40 – 7.35 (m, 1H), 7.29 – 7.24 (m, 2H), 7.19 – 7.11 (m, 2H), 6.82 – 6.70 (m, 4H), 4.56 (t, *J* = 5.0 Hz, 2H), 4.48 (s, 2H), 4.30 (t, *J* = 5.0 Hz, 2H), 4.09 (q, *J* = 6.1 Hz, 2H), 3.36 (t, *J* = 5.9 Hz, 2H);

^{13}C NMR (126 MHz, $CDCl_3$) δ 167.57, 154.46, 153.53, 153.35, 142.73, 134.81, 133.62, 133.35, 128.50, 128.45, 122.85, 122.80, 122.46, 122.24, 119.65, 113.16, 113.02, 112.20, 112.04, 109.09, 67.75, 67.03, 43.08, 35.67, 27.61;

HRMS for $[C_{25}H_{24}Br_2N_3O_3]^+$ $(M + H)^+$ 572.0181, found 572.0139.

24: Method E, 83%, white solid; **^{1}H NMR (600 MHz, $CDCl_3$)** δ 7.97 (s, 1H), 7.71 (d, *J* = 7.8 Hz, 1H), 7.34 (d, *J* = 7.8 Hz, 1H), 7.32 – 7.16 (m, 7H), 6.97 (t, *J* = 7.3 Hz, 1H), 6.92 (t, *J* = 7.3 Hz, 1H), 6.85 (dd, *J* = 11.2, 8.3 Hz, 4H), 4.45 (s, 2H), 4.33 (t, *J* = 6.8 Hz, 2H), 3.91 – 3.85 (m, 4H), 3.05 (t, *J* = 6.0 Hz, 2H), 2.31 – 2.21 (m, 2H);

^{13}C NMR (151 MHz, $CDCl_3$) δ 168.34, 158.21, 157.22, 152.64, 142.59, 134.97, 129.64, 129.58, 122.46, 122.12, 121.82, 121.24, 119.27, 114.62, 114.38, 109.39, 67.22, 63.58, 40.09, 35.76, 29.20, 26.89;

HRMS for $[C_{26}H_{28}N_3O_3]^+$ $(M + H)^+$ 430.2127, found 430.2095.

25: Method E, 69%, white solid; **^{1}H NMR (500 MHz, $CDCl_3$)** δ 7.94 (s, 1H), 7.75 – 7.66 (m, 1H), 7.31 – 7.23 (m, 3H), 7.19 (t, *J* = 8.0 Hz, 2H), 6.91 (t, *J* = 7.4 Hz, 1H), 6.83 (d, *J* = 8.0 Hz, 2H), 4.47 (s, 2H), 3.94 (q, *J* = 6.1 Hz, 2H), 3.66 (s, 3H), 3.05 (t, *J* = 6.1 Hz, 2H);

^{13}C NMR (126 MHz, $CDCl_3$) δ 168.50, 157.20, 152.62, 142.40, 135.73, 129.58, 122.37, 122.06, 121.85, 119.14, 114.61, 109.07, 67.24, 35.73, 29.61, 27.14;

HRMS for $[C_{18}H_{20}N_3O_2]^+$ $(M + H)^+$ 310.1550, found 310.1527.

26: Method E, 81%, white solid; **^{1}H NMR (500 MHz, $CDCl_3$)** δ 7.95 (s, 1H), 7.75 (d, *J* = 7.8 Hz, 1H), 7.32 – 7.16 (m, 8H), 7.05 – 7.00 (m, 2H), 6.93 (t, *J* = 7.3 Hz, 1H), 6.85 (d, *J* = 8.2 Hz, 2H), 5.29 (s, 2H), 4.44 (s, 2H), 3.90 (q, *J* = 6.1 Hz, 2H), 3.00 (t, *J* = 6.0 Hz, 2H);

^{13}C NMR (126 MHz, $CDCl_3$) δ 168.44, 157.22, 152.81, 142.51, 135.68, 135.45, 129.61, 129.09, 128.05, 126.26, 122.70, 122.27, 121.87, 119.33, 114.65, 109.58, 67.23, 46.86, 35.75, 27.25;

HRMS for $[C_{24}H_{24}N_3O_2]^+$ $(M + H)^+$ 386.1863, found 3386.1841.

27: Method E, 48%, white solid; **^{1}H NMR (250 MHz, $CDCl_3$)** δ 7.99 (s, 1H), 7.78 – 7.66 (m, 1H), 7.36 – 7.13 (m, 6H), 6.98 – 6.78 (m, 3H), 5.84 – 5.64 (m, 1H), 5.11 – 4.97 (m, 2H), 4.47 (s, 2H), 4.13 (t, *J* = 7.3 Hz, 2H), 3.96 (q, *J* = 6.1 Hz, 2H), 3.05 (t, *J* = 6.1 Hz, 2H), 2.50 (q, *J* = 7.1 Hz, 2H);

^{13}C NMR (63 MHz, $CDCl_3$) δ 168.43, 157.19, 152.35, 142.57, 134.72, 133.44, 129.55, 122.32, 122.02, 121.81, 119.22, 118.46, 114.60, 109.37, 67.22, 43.06, 35.79, 33.82, 27.12;

HRMS for $[C_{21}H_{24}N_3O_2]^+$ $(M + H)^+$ 350.1863, found 350.1843.

28: Method E, 19%, white solid; **^{1}H NMR (250 MHz, DMSO)** δ 8.37 (t, *J* = 5.6 Hz, 1H), 7.77 (s, 1H), 7.63 – 7.50 (m, 1H), 7.44 – 7.10 (m, 6H), 7.02 – 6.88 (m, 3H), 4.84 (s, 2H), 4.47 (s, 2H), 3.64 (q, *J* = 6.8 Hz, 2H), 2.99 (t, *J* = 7.1 Hz, 2H);

^{13}C NMR (63 MHz, DMSO) δ 169.00, 168.25, 157.99, 153.99, 142.46, 135.94, 129.95, 122.24, 121.89, 121.71, 118.81, 115.15, 110.09, 67.33, 45.72, 36.64, 27.08;

HRMS for $[C_{19}H_{21}N_4O_3]^+$ $(M + H)^+$ 353.1608, found 353.1575.

29: Method E, 82%, white solid; **^{1}H NMR (500 MHz, $CDCl_3$)** δ 8.08 (s, 1H), 7.79 – 7.69 (m, 1H), 7.42 – 7.18 (m, 5H), 6.95 (t, *J* = 7.4 Hz, 1H), 6.89 (d, *J* = 7.9 Hz, 2H), 4.51 (s, 2H), 4.28 (t, *J* = 5.3 Hz, 2H), 3.98 (q, *J* = 6.1 Hz, 2H), 3.68 (t, *J* = 5.3 Hz, 2H), 3.28 (s, 3H), 3.15 (t, *J* = 6.1 Hz, 2H);

^{13}C NMR (126 MHz, $CDCl_3$) δ 168.38, 157.29, 153.36, 142.57, 134.98, 129.58, 122.33, 122.08, 121.81, 119.25, 114.67, 109.26, 70.68, 67.31, 59.13, 43.73, 35.83, 27.10;

HRMS for $[C_{20}H_{24}N_3O_3]^+$ $(M + H)^+$ 354.1812, found 354.1793.

To a solution of 3-(2-phenoxyacetamido)propanoic acid (0.9 mmol, 1.0 equiv.) in CH_2Cl_2 (15 mL) at 0 °C was added DMF (1 drop) and oxalyl chloride (1.07 mmol, 1.2 equiv.), and the suspension was warmed to 25 °C over 45 min and stirred at 25 °C for 30 min. The mixture was then concentrated, and the residue was dissolved in *n*-butanol (5 mL), N^1-phenylbenzene-1,2-diamine (0.9 mmol, 1.0 equiv.) was added, and the mixture was heated to 110 °C. After 18 h, the reaction mixture was cooled and poured over water (10 mL), extracted with CH_2Cl_2 (3 × 10 mL), dried over $MgSO_4$, and concentrated to a brown oil. Purification by column chromatography (20:1 $CHCl_3$/MeOH) gave **30** in 51% yield.

30: Yellow oil; **^{1}H NMR (500 MHz, $CDCl_3$)** δ 8.03 (s, 1H), 7.81 (d, *J* = 8.0 Hz, 1H), 7.64 – 7.53 (m, 3H), 7.38 – 7.31 (m, 3H), 7.26 (t, *J* = 8.1 Hz, 3H), 7.16 (d, *J* = 8.0 Hz, 1H), 6.97 (t, *J* = 7.4 Hz, 1H), 6.92 (d, *J* = 8.0 Hz, 2H), 4.50 (s, 2H), 3.90 (q, *J* = 6.1 Hz, 2H), 3.00 (t, *J* = 6.0 Hz, 2H);

^{13}C NMR (126 MHz, $CDCl_3$) δ 168.33, 157.27, 152.63, 142.40, 136.45, 135.47, 130.08, 129.64, 129.13, 127.20, 123.00, 122.62, 121.88, 119.17, 114.68, 110.17, 67.24, 35.96, 27.59;

HRMS for $[C_{23}H_{22}N_3O_2]^+$ $(M + H)^+$ 372.1706, found 372.1679.
